# Photocatalytic Reduction of CO_2_ into CO with Cyclometalated Pt(II) Complexes of N^C^N Pincer Dipyridylbenzene Ligands: A DFT Study

**DOI:** 10.3390/molecules29020403

**Published:** 2024-01-14

**Authors:** Antonia Sarantou, Athanassios Tsipis

**Affiliations:** Laboratory of Inorganic Chemistry, Department of Chemistry, University of Ioannina, 45110 Ioannina, Greece; a.sarantou@uoi.gr

**Keywords:** photocatalytic CO_2_ reduction, DFT/TDDFT, Pt(II) pincer complexes, T_1_ excited state electrochemistry, CO_2_ fixation/activation by Pt

## Abstract

In this work, density functional theory (DFT) calculations were employed to study the photocatalytic reduction of CO_2_ into CO using a series of Pt(II) square planar complexes with the general formula [Pt(5-R-dpb)Cl] (dpb = 1,3-di(2-pyridyl)benzene anion, R = H, *N*,*N*-dimethylaniline,T thiophene, diazaborinine). The CO_2_-into-CO conversion process is thought to proceed via two main steps, namely the photocatalytic/reduction step and the main catalytic step. The simulated absorption spectra exhibit strong bands in the range 280–460 nm of the UV-Vis region. Reductive quenching of the T_1_ state of the complexes under study is expected to be favorable since the calculated excited state redox potentials for the reaction with sacrificial electron donors are highly positive. The redox potentials reveal that the reductive quenching of the T_1_ state, important to the overall process, could be modulated by suitable changes in the N^C^N pincer ligands. The CO_2_ fixation and activation by the three coordinated Pt(II) catalytically active species are predicted to be favorable, with the Pt–CO_2_ bond dissociation energies *D*_0_ in the range of −36.9–−10.3 kcal/mol. The nature of the Pt–CO_2_ bond of the Pt(II) square planar intermediates is complex, with covalent, hyperconjugative and H-bonding interactions prevailing over the repulsive electrostatic interactions. The main catalytic cycle is estimated to be a favorable exergonic process.

## 1. Introduction

The problem of global overheating dates back long before the industrial revolution. The excessive use of fossil fuels and the massive release of greenhouse gases into the atmosphere have turned the term “global warming” into an uncomfortable reality of modern life. CO_2_, although not a primary greenhouse gas, nevertheless has a significant contribution to global warming. In 2012, global CO_2_ emissions, which come mainly from the burning of fossil fuels, reached a total of 34.5 billion tons [1].

The steadily increasing concentration of CO_2_ in the atmosphere is now a real problem. The CO_2_ emissions from the massive consumption of fossil fuels are largely responsible for global climate change: an increase in the concentration of CO_2_ in the atmosphere causes global warming, as well as ocean acidification from the uptake of atmospheric CO_2_ [2].

Man’s growing needs for energy lead to the ominous conclusion that CO_2_ emissions will not remain stagnant but will increase even more. This results in the scientific community focusing on how the abundance of CO_2_ will be used to produce materials of commercial interest. Only 1‰ of the total abundance of CO_2_ is currently used for chemical synthesis, which is mainly due to its chemical inertness (it is highly oxidized in nature) but also to the fact that capturing and storing CO_2_ is an expensive process [3].

Many studies have focused on how to deal with the problem of the continuous increase in atmospheric CO_2_ while, at the same time, there are ongoing efforts and studies not only to capture CO_2_ but also to catalytically convert it into “fine chemicals”, as well as CO, CH_3_OH and HCOOH [3]. A recent study by Audisio et al. demonstrated that CO, produced via a very fast photocatalytic CO_2_-into-CO conversion (<10 min), could be valorized to produce radiopharmaceuticals [4].

Among various strategies to achieve this goal, the greatest and most attractive challenge is the efficient conversion of CO_2_ into useful compounds using sunlight as an energy source [5]. Studies on this issue have shown the photochemical and electrochemical reduction of CO_2_ into CO, into CH_3_OH and also into HCOOH, using transition metal electrodes, metal complexes, semiconductors, as well as some organic molecules [6,7,8,9].

Much of the research has focused on how to sequester CO_2_ from coal plant emissions, which account for 76% of all global emissions [10]. Recent advances in catalyst design combined with the reduced cost of clean energy has made the use of CO_2_ more attractive as a feedstock for chemical production. Particular interest has gathered in the conversion of CO_2_ into CO as a step in the petrochemical production process [11].

Mainly, there are two approaches to CO_2_ reduction, namely either via heterogeneous or homogeneous catalysis. Both approaches can be achieved either electrocatalytically or photocatalytically. Heterogeneous electrocatalytic CO_2_ reduction is carried out using electrolysis with the aid, for example, of nanostructured materials [12], while heterogeneous photocatalytic CO_2_ reduction mainly utilizes various types of semiconductors as photocatalysts [13]. On the other hand, homogeneous electrochemical/photochemical CO_2_ reduction is mainly based on transition metal complexes [14]. So far, coordination complexes of numerous metals have been studied as catalysts, such as the first row Mn, Fe, Co, Ni and Cu, also the second row Ru, Rh, Pd, as well as the third row W, Re, Os, Ir [14].

Since 1970, a great number of works have appeared in the literature concerning electrochemical/photochemical CO_2_ reduction using aza macrocyclic and polypyridine complexes [15]. Along these lines, complexes of the earth-abundant Fe, Co and Ni transition metals with porphyrin-like ligands have attracted much interest. Thus, for example, a porphyrin-like aminopyridyl macrocyclic Co complex was found to be highly selective (98%) for CO production [16,17]. Fe tetraphenyl porphyrins have been demonstrated to be very active for the conversion of CO_2_ into CO [15]. Water-soluble Fe-porphyrins showed very high activity (close to 100%) for CO production from CO_2_ during electrolysis under low overpotential [18]. Phthalocyanine complexes of another earth-abundant, first-row Group VIII metal, namely Ni, were shown to very efficient for CO production, with nearly 100%, just like similar Co complexes [19]. In addition, other aza macrocyclic and polypyridine complexes have shown great potential for CO_2_ conversion. Lioret-Fillol et al., in a combined experimental/theoretical work, studied extensively the mechanism of CO_2_ reduction using a very promising Co aminopyridine catalyst [20]. Iron and cobalt quaterpyridine complexes proved to be highly efficient and selective for photocatalytic CO_2_ reduction [21]. Complexes of Ni and Co with the aza-crown ether cyclam ligand have been extensively studied for CO_2_ conversion [15]. In a pioneering study, Tinnemans et al. employed Ni as well as Co cyclams to photocatalytically reduce CO_2_ [22]. Since then, numerous studies on cyclam complexes have appeared. A simple Ni cyclam complex exhibited high catalytic activity for CO_2_ reduction, and this was attributed to the complex stabilization due to the presence of a cyclam ring, as well as to an acidic N-H proton [23,24,25]. Finally, studies have demonstrated that complexes of Mn with electron-rich ligands such as bipyridine are effective in CO_2_ reduction [26].

Up to now, Ru and Re complexes have received the major focus and are the most widely studied. Probably, the interest in these complexes stems from the inspiring seminal works of Lehn et al. [27,28,29], who studied electro/photocatalytic CO_2_ reduction using Re(I) and Ru(II) complexes. Since then, many other Re(I) and Ru(II) complexes have been studied for electro/photocatalytic CO_2_ reduction [2,30,31].

The reduction of CO_2_ into either CO or HCOOH proceeds via the following chemical equations:

CO_2_ + 2e^−^ + 2H^+^ → CO + H_2_O (1)

CO_2_ + 2e^−^ + 2H^+^ → HCOOH (2)

The reduction potentials of the above 2e^−^/2H^+^ reductions are −0.52 and −0.61 V vs. SHE [31] and the e^-^ is provided either by the electrodes in the case of electrocatalysis or by a “sacrificial donor”, e.g., TEOA in the case of photocatalysis. At least for Re(I) and Ru(II) complexes, the proposed mechanism involves, in almost all cases, the formation of a coordinatively unsaturated, 17e^−^, very reactive intermediate [2]. Thus, for example, in the reduction of CO_2_ using *cis*-[Ru(bpy)_2_(CO)X]^n+^, a mixture of CO/HCOO^−^ is obtained [32]. Two mechanisms are proposed to operate in conjunction, having in common the very reactive, five-coordinated *cis*-[Ru(bpy)_2_(CO)]^0^ intermediate. The product selectivity depends upon the reactivity of this intermediate towards CO_2_ or H^+^ [2]. The so-called *η^1^–CO_2_ complex mechanism* operates upon CO_2_ capture through the *cis*-[Ru(bpy)_2_(CO)]^0^ intermediate, forming the *cis*-[Ru(bpy)_2_(CO)(*η*^1^-CO_2_)] adduct, which, upon protonations, yields CO. On the other hand, in the so-called *hydride mechanism*, operating simultaneously with the *η*^1^–CO_2_ complex mechanism, the *cis*-[Ru(bpy)_2_(CO)]^0^ intermediate captures H^+^, forming a hydrido intermediate, which upon reduction and CO_2_ insertion into the Ru–H bond, yields HCOO^−^. However, it should be noticed that the unsaturated five-coordinated [Ru(bpy)_2_(CO)]^0^ intermediate has not yet been detected [2]. Accordingly, there have been proposed two other mechanisms for the reduction of CO_2_ [29].

Ishitani et al. [33], using various spectroscopic techniques, demonstrated the existence of the *fac*-[Re^I^(bpy)(CO)_3_(OCH_2_CH_2_NR_2_)] formed upon the coordination of the TEOA^−^ anion to the metal center of the 17e^−^ five-coordinated *fac*-[Re^I^(bpy)(CO)_3_]^+^ intermediate. They also demonstrated the existence of the *fac*-[Re^I^(bpy)(CO)_3_(O(CO)OCH_2_CH_2_NR_2_)] produced upon the insertion of CO_2_ into the Re–O bond of the *fac*-[Re^I^(bpy)(CO)_3_(OCH_2_CH_2_NR_2_)] intermediate. A similar CO_2_ insertion product has been observed also for the [Ru(dmb)_2_(CO)_2_]^2+^ complex in DFM-TEOA solution [34]. The CO_2_-into-CO reduction upon the coordination of the TEOA^−^ anion and subsequent CO_2_ insertion has also been scrutinized for Re(I) complexes with the aid of DFT calculations [35].

Finally, based upon both experimental [36,37] as well as theoretical [38] studies, there is also another proposed mechanism for CO_2_ reduction using transition metal complexes, thought to operate via the dimerization of the five-coordinated, very reactive intermediate. Hence, Muckerman et al. [38], employing DFT electronic structure calculations, showed that CO_2_ is inserted between two five-coordinated [Re(dmb)(CO)_3_]· (dmb = 4,4′-dimethyl-bpy) radical intermediates, bridging their metal centers. Through a second CO_2_ addition to this dimeric species, and via a transition state, CO is produced.

Although, so far, complexes of a large number of transition metals have been studied for electro/photocatalytic CO_2_ reduction, Pt complexes have attracted very little attention. Ceballos et al. [39] demonstrated electrocatalytic CO_2_ reduction into formate using the [Pt(dmpe)_2_](PF_6_)_2_ complex with high Faradaic efficiency and low overpotential. The vast majority of studies on CO_2_ reduction involving Pt metals have been devoted to heterogeneous catalysis. For example, Zhang et al. [40] dealt with the photocatalytic reduction of CO_2_ into CH_4_ under the influence of ultraviolet radiation, with water as solvent on a TiO_2_ catalyst with Pt. Quite numerous other studies have also appeared in the literature [41,42,43,44,45,46,47] concerning heterogeneous CO_2_ transformation. Finally, an enormous amount of work has been devoted to the ubiquitous photocatalytic water splitting for H_2_ production using Pt nanoparticles [48,49].

To the best of our knowledge, neither experimental nor theoretical studies have been undertaken so far on photocatalytic CO_2_-into-CO reduction employing Pt(II) square planar complexes as the catalysts. Accordingly, we instigated scrutinizing, by means of electronic structure calculations, the photocatalytic reduction of CO_2_ into CO using square planar Pt(II) cyclometalated complexes bearing N^C^N-coordinating substituted dipyridylbenzene ligands of the general formula [Pt(5-R-dpb)Cl] (dpb = 1,3-di(2-pyridyl)benzene anion, R = H, *N*,*N*-dimethylaniline, thiophene, bodipy). Our targets are (1) to study the photophysical properties of these complexes in order to investigate whether they could be used as both photosensitizers as well as catalysts, (2) to explore the mechanism of the catalytic reaction and (3) to probe how the nature of the N^C^N-substituted bipyridyl ligand affects both the photophysical properties and mechanism of action of the Pt(II) complexes under study.

## 2. Results and Discussion

**Scheme 1:**
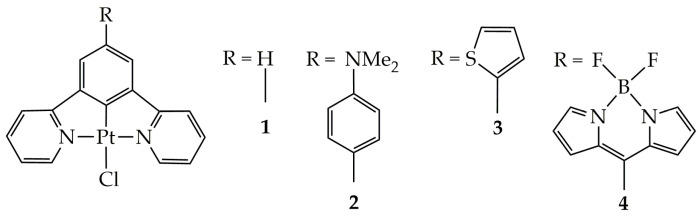
The molecular structures of Pt(II) complexes under study.

The molecular structure of the square planar Pt(II) complexes employed for the CO_2_-into-CO photocatalytic reduction in this study is depicted in Scheme 1.

**Figure 1:**
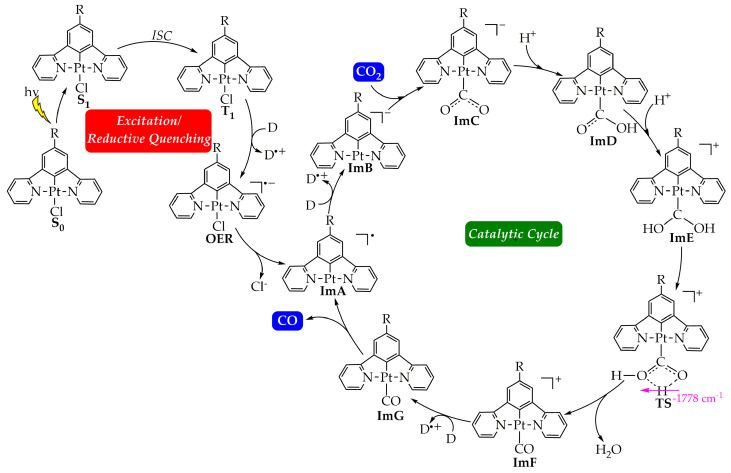
Proposed calculated mechanism for the CO_2_-into-CO photocatalytic reduction by the [Pt(5-R-dpb)Cl], **1**–**5** Pt(II) complexes.

The CO_2_-into-CO photocatalytic reduction is thought to proceed via two steps: (1) the photoexcitation/reductive quenching step and (2) the actual catalytic cycle. The two steps are depicted in Figure 1 along with the molecular structures of all the species participating in the overall process. Let us start by examining first the initial step, which henceforth we shall call the photoexcitation/reductive quenching step, and next we will deal with the second step, which we shall call the catalytic cycle step.

### 2.1. Photoexcitation/Reductive Quenching Step

**Figure 2:**
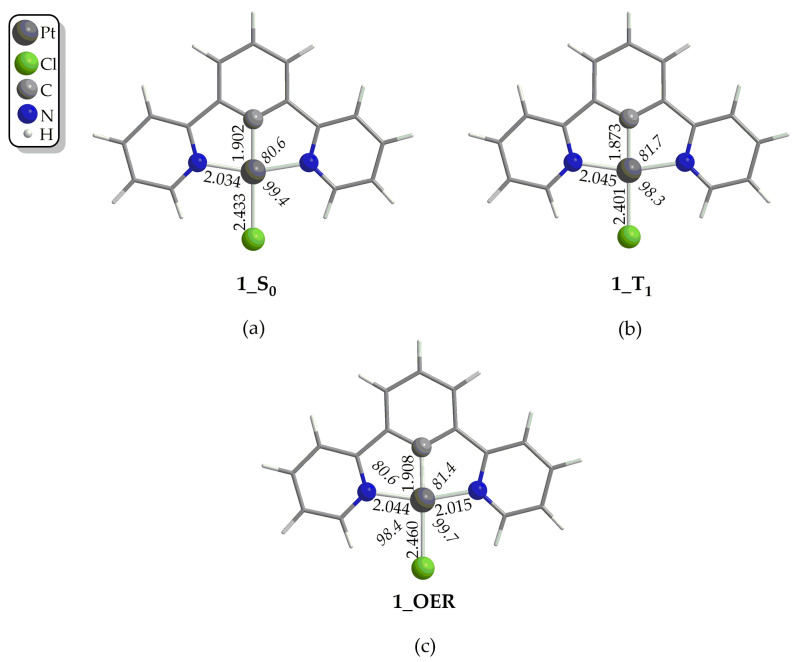
Optimized geometries of **1** in (**a**) S_0_ ground state, (**b**) in T_1_ excited state and (**c**) of One-Electron-Reduced form, **1_OER**, calculated at the PBE0-GD3BJ/Def2-TZVP level, in DCM solvent.

In the first step, the Pt(II) catalyst, upon irradiation, is excited to its first singlet excited state, S_1_. For a third-row transition metal complex like those under study, it is expected for the transition to occur from the S_1_ state to the first triplet excited state T_1_ via Intersystem Crossing (*ISC*) due to Spin–Orbit Coupling (*SOC*). Then, if the lifetime *τ* of the T_1_ state of the complex is large enough, reductive quenching occurs, where the complex, upon accepting an e^−^ from a donor D, e.g., TEOA or TEA, yields the One-Electron-Reduced (OER) complex (Figure 1). Notice that the experimentally determined *τ* of the T_1_ state of **1** has been found to be 7.2 μs in a CH_2_Cl_2_ solvent at 298 °C [50], which is large enough to permit reductive quenching. The optimized geometries of **1**–**4** in their S_0_ and T_1_ states, as well the geometries of the respective OER species, along with the selected structural parameters calculated at the PBE0-GD3BJ/Def2-TZVP level, in the DCM solvent are given in Figure 2, as well as in Figures S1–S3 of the Supplementary Materials. The calculated structural parameters of **1** in its S_0_ ground state are in excellent agreement with the respective structural parameters derived from the X-ray structural analysis of this complex [51]. Thus, for example, the difference between the calculated and X-ray derived bond lengths around the coordination sphere is in the range of only 0.001–0.016 Å, while the calculated bond angles differ by only 0.3–0.5° from the respective X-ray experimental values. Upon excitation to the T_1_ excited state, no significant differences in the structural parameters could be observed (Figure 2b and Figures S1–S3). Accordingly, the changes in the Pt–Cl and Pt–C coordination bonds are in the ranges 0.03–0.001 Å and 0.009–0.019 Å, respectively, while the changes in the Pt–N coordination bond lie within the range 0.0–0.015 Å. On the other hand, the bond angles around the coordination sphere change slightly by about 1° in all cases, **1**–**4**. Finally, the same holds also for the organic framework of the N^C^N pincer ligand, where the structural changes upon S_0_ → T_1_ excitation are negligible. Next, the one-electron reduction of the T_1_ state causes more significant structural changes. Thus, there are more obvious structural differences between the optimized geometries of the T_1_ state and the OER species (Figure 2c and Figures S1–S3). The most striking structural change is observed in the Pt–Cl coordination bond, which is elongated by 0.007–0.032 Å in the **OER** species as compared to the T_1_ state species. The same holds also if we compare the **OER** species with the S_0_ state species. Also, the Pt–C coordination bond is elongated by 0.035 Å in contrast to the two Pt–N coordination bonds, which are shortened.

Finally, the bond angles around the coordination sphere do not change significantly upon one-electron reduction.

#### 2.1.1. Absorption Spectra

**Figure 3:**
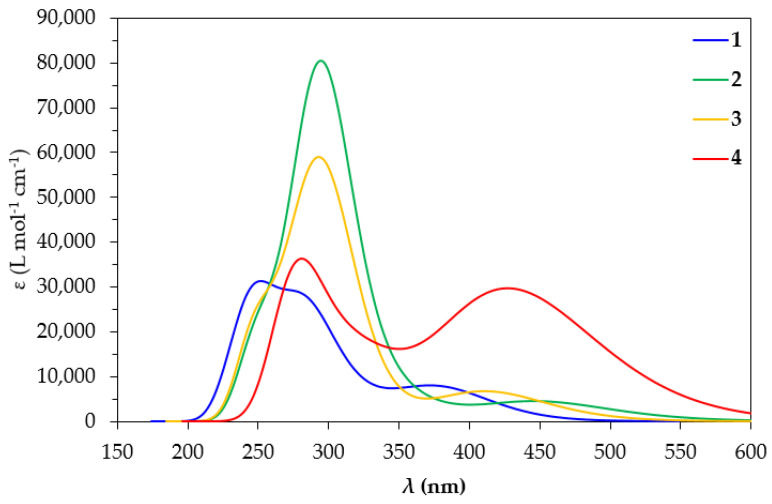
Simulated absorption spectra of **1**–**4** in DCM at the TDDFT/PBE0/Def2-TZVP level.

Since the excitation of the precursor complexes **1**–**4** is a prerequisite in photocatalytic CO_2_ conversion, we set out to study their absorption spectra in DCM by means of TDDFT calculations. Accordingly, we employed the two most popular functionals (vide infra), for the simulation of the absorption spectra in DCM of the complexes under study, namely PBE0 and CAM-B3LYP, using the Def2-TZVP basis set in both cases. The simulated absorption spectra of **1**–**4** calculated using the TDDFT/PBE0/Def2-TZVP computational protocol are depicted in Figure 3, while those obtained using the TDDFT/CAM-B3LYP/Def2-TZVP computational protocol are given in Figure S4 of the Supplementary Materials.

The simulated absorption spectrum of **1** calculated at the PBE0/Def2-TZVP level in DCM is in agreement with the experimental absorption spectrum derived by Williams et al. for **1** in DCM [50]. Thus, the simulated spectrum of **1** exhibits a band at 380 nm, reproducing excellently the experimental maximum found at 380 nm in the experimental spectrum as well. On the other hand, the simulated absorption spectrum of **1** at the CAM-B3LYP/Def2-TZVP level (Figure S4) shows a maximum at 344 nm, underestimating the experimental maximum by about 36 nm. The simulated absorption spectra using both computational protocols employed in this study exhibit also higher energy maxima below 300 nm, underestimating the respective maxima found in the experimental spectra at 330 nm and below. It should be noticed that overall, the simulated absorption spectra of **1**–**4** at the CAM-B3LYP/Def2-TZVP level are blue-shifted by 30–40 nm compared to those calculated at the PBE0/Def2-TZVP level in DCM. Let us further analyze the spectra calculated at the PBE0/Def2-TZVP level due to their better agreement with the experiment. Perusal of Figure 3 reveals that the simulated absorption spectra at the PBE0/Def2-TZVP level in DCM of **1**, **2**, **3** and **4** exhibit low-energy bands at 380, 460, 420 and 428 nm, respectively. In addition, the simulated absorption spectra of all complexes exhibit high-energy bands in the region of 250–300 nm.

**Figure 4:**
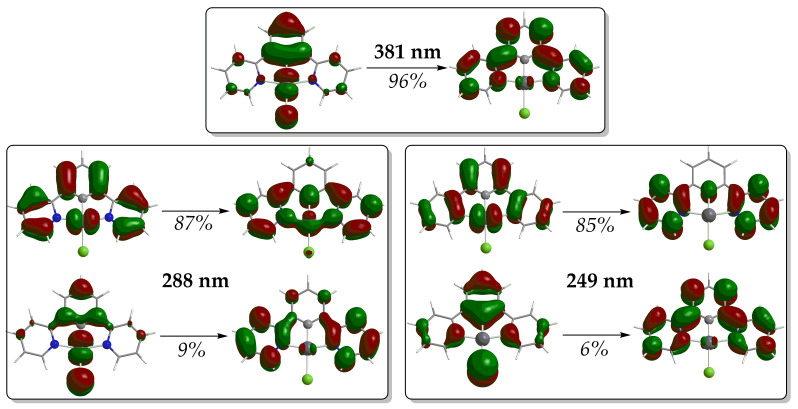
NTO pairs for the most significant electronic transitions of the simulated absorption spectra of **1** (hole *h*^+^ on the left, electron *e^−^* on the right).

In terms of Natural Transition Orbitals (NTOs), the band peaking at 380 nm arises mainly from an electronic transition at 381 nm, which is due to the electron excitation between the NTO pair depicted in Figure 4. Based upon the shape of these NTOs, the band shows a mixed assignment comprising of Metal-to-Ligand Charge Transfer (MLCT), Intra-Ligand (IL) and Ligand-to-Ligand Charge Transfer (LL’CT) characters (MLCT/IL/L L′CT). The same assignment holds also true for the low-energy bands in the region 420.

The calculated absorption spectra of **2** and **3** exhibit a band around 460 nm. The former exhibits a band around 460 nm while the latter exhibits a band around 420 nm, arising from electronic transitions at 452 and 414 nm, respectively. Based upon the shapes of the NTO pairs (Figures S5 and S6 of Supplementary Materials) participating in these electronic transitions, these low-energy bands are assigned as MLCT/IL/LL′CT. The exception is complex **4**, where the band around 428 nm arises mainly from an electronic transition at 420 nm, assigned as MLCT/IL.

Next, the simulated absorption spectra exhibit high-energy bands in the UV region (Figure 3). The spectrum of complex **1** shows two such high-energy bands around 250 and 280 nm. The latter arises mainly from an electronic transition at 288 nm while the former is mainly due to an electronic transition at 249 nm. The shape of the NTOs involved in these transitions indicates that the high-energy bands in the UV are of MLCT/IL/LL′CT character. On the other hand, the absorption spectra of complexes **2**–**4** exhibit just one distinct high-energy band in the UV region. This high-energy band appears around 295 nm for **2** and **3** and around 284 nm for **4**. For complexes **2** and **3**, these bands are also of the highest intensity in their absorption spectra. For **2**, the most intense high-energy band arises mainly from an electronic transition at 298 nm, involving almost solely an excitation between a pair of NTOs (Figure S5). Based upon the shape of these NTOs, the band at 285 nm for **2** is assigned as MLCT/IL. Conversely, for **3**, the high-energy band arises mainly from an electronic transition at 279 nm, which is, however, due to excitations involving two NTO pairs (Figure S6). The band around 295 nm for **3** is assigned as MLCT/IL/LL′CT in accordance with the shape of the NTOs involved.

#### 2.1.2. Excited State Electrochemistry

**Scheme 2:**
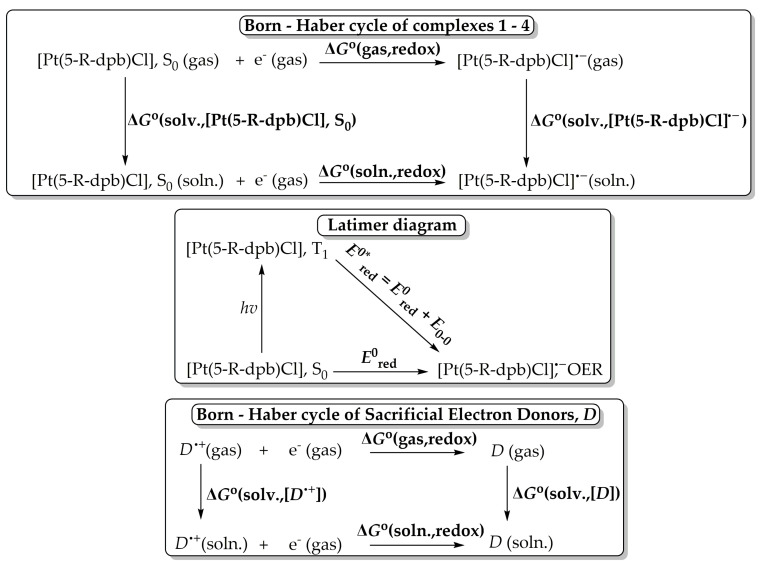
Born–Haber cycle for calculation of ground-state reduction potentials *E*^0^_red_ (**above**), Latimer diagram for the calculation of T_1_ excited state reduction potentials *E*^0*^_red_ (**middle**) and Born–Haber cycle for calculation of sacrificial electron donor *D* (=TEOA or TEA) reduction potentials (**below**).

One of the most important steps in photocatalytic CO_2_ conversion using transition metal catalysts is the reductive quenching of their T_1_ state upon receiving an e from a sacrificial donor, e.g., TEOA. Therefore, we instigated studying the T_1_ excited state reduction potentials using DFT calculations. The ground-state redox potentials could be calculated by employing the Born–Haber cycle [52] depicted schematically in Scheme 2.

The standard absolute ground-state reduction potential *E*^0^_red_ is calculated using the following equation:

*E*^0^_red_ = −Δ*G*°(soln. redox)/*ZF*, (3)

where *F* is the Faraday constant (23.061 kcal per volt gram equivalent) and *Z* is the unity for the one-electron redox processes. Δ*G*°(soln., redox) is obtained from the following equation:

Δ*G*°(soln., redox) = Δ*G*°(gas, redox) + Δ*G*°(solv.,[Pt(5-R-dpb)Cl]^•−^) − Δ*G*°(solv.,[Pt(5-R-dpb)Cl], S_0_) (4)

In Table 1 are tabulated the values of the reduction potentials calculated for complexes **1**–**4** calculated at the PBE0/def2-TZVP level. In Table 1 are given also the reduction potentials of the sacrificial electron donors *D* TEOA and TEA at the same level of theory.

Inspection of Table 1 reveals that the oxidizing ability of the Pt(II) complexes in their T_1_ state follows the series **2** > **1** > **3** > **4**, i.e., **2** is anticipated to accept an e^−^ in the T_1_ state and be the most easily reduced amongst all the complexes under study. In contrast, the oxidizing ability of the Pt(II) complexes in their S_0_ ground state follows exactly the same order as that observed for the T_1_ state, following the order **2** > **1** > **3** > **4**. We could draw the same conclusion based also upon the values of the overall redox potential *Ε*^0^ calculated for the redox reaction of **1**–**4** in the T_1_ state with TEOA or TEA, which is favorable since it is positive in all cases. The most favorable redox reaction will be with **2**, followed by **1**, **3** and **4**.

### 2.2. Catalytic Cycle Step

The initial photoexcitation/reductive quenching step ends with the formation of the **OER** species. The latter, upon losing a chloride ligand, yields a three-coordinated intermediate **ImA**, which starts the actual catalytic cycle (Figure 1). **ImA** could be considered the “true” catalyst while the initial Pt(II) complex could be considered a “precatalyst”. Based upon earlier studies [2], the catalytic cycle step could proceed upon the one-electron reduction of **ImA** with the aid of an electron donor D like, for example, TEOA, yielding **ImB**. Next, there is one of the most important steps, i.e., the CO_2_ addition to the metal center of **ImB**. After two subsequent protonations (e.g., with TEOA, which can act as proton source as well [2]), we arrive, through the transition state **TS**, to the carbonyl intermediate **ImF**. A second electron reduction of the latter yields **ImG**, from which we obtain CO and the initial “true” catalyst **ImA**.

#### 2.2.1. The η^1^–CO_2_ Complex

Since the CO_2_ capture/activation step, upon coordination to the metal center of the catalyst, is considered to be of paramount importance in electro/photocatalytic CO_2_ conversion [53,54], we set out to study in depth the structural, bonding and electronic properties of **ImC**.

##### Structural Properties

**Figure 5:**
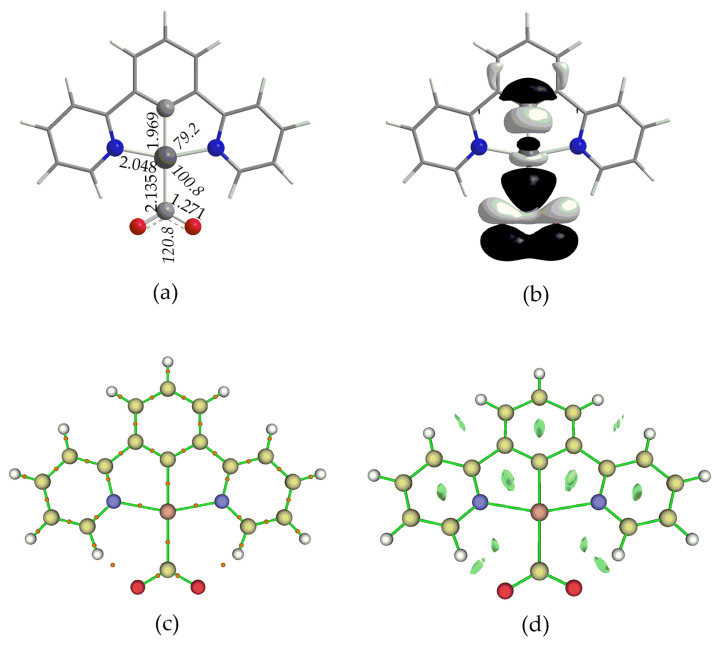
(**a**) Optimized geometry of **1_ImC**, (**b**) 3D surface plot of HOMO, (**c**) BCPs (orange spheres) and (**d**) 3D surface of RDG function.

The optimized geometry of **1_ImC** calculated at the PBE0-D3/Def2-TZVP level, in DCM solvent, is depicted in Figure 5a, while those of **2_ImC**, **3_ImC** and **4_ImC** are given in Figures S5–S7 of the Supplementary Material.

Perusal of Figure 5a and Figures S5–S7 reveals that, in all complexes, CO_2_ is coordinated to the Pt metal center via the C atom in an *η*^1^-bonding mode. The complex formed via the *η*^1^–CO_2_ bonding mode is considered to be of pivotal importance to the majority of the electron transfer reactions for photo/electrocatalytic CO_2_ conversion [2,53]. The Pt–CO_2_ bond length is estimated to be 2.132–2.135 Å, indicative of a bonding interaction. Upon coordination to the Pt metal center, the CO_2_ molecule is activated and changes from linear to bended, with a <O–C–O bond angle equal to 120.8°, while the C–O bonds are equal to 1.270 Å, elongated by about 0.110 Å as compared to the “free” CO_2_ molecule (1.160 Å).

##### Bonding and Electronic Properties

The covalent nature of the Pt–CO_2_ bond is reflected in the shape of HOMO calculated for **1_ImC** and depicted schematically in Figure 5b (the HOMOs of **2_ImC**, **3_ImC** and **4_ImC**). The HOMO is a bonding MO mainly constructed by the in-phase combination of the Pt d_z2_ AO with the π* MO of CO_2_, in line with previous studies [51]. The bond dissociation energy *D*_0_ of the Pt–CO_2_ bond was found to be −36.8, −36.9, −35.1 and −10.3 kcal/mol for **1_ImC**, **2_ImC**, **3_ImC** and **4_ImC**, respectively. Obviously, substitution into position 5 of the N^C^N pincer ligand has no significant impact on *D*_0_(Pt–CO_2_). The exception is complex **4**, where the introduction of the diazaborinine group reduces *D*_0_(Pt–CO_2_) by a third as compared to the rest of the complexes. Nevertheless, based upon the magnitude of *D*_0_(Pt-CO_2_), the interaction between **ImB** and CO_2_ is expected to be a relatively favorable interaction.

To further analyze the Pt–CO_2_ bond in **ImC**, we employed the atoms in molecules (AIM) method. In Figure 5c are depicted the bond-critical points (BCPs) found using the AIM method for **1_ImC** (similar BCPs are detected also for the intermediate **ImC** formed by complexes **2**–**4**). According to Bader’s [55,56] theory, the presence of a BCP between two atoms indicates bond formation. Inspection of Figure 5c reveals the existence of a BCP between the Pt metal center and C atom of the CO_2_ ligand, indicating a Pt–CO_2_ bonding interaction. It has been proposed [57,58], based upon the values of certain properties of BCPs, that the bonding interactions could be classified into three categories: (a) pure closed-shell interactions (e.g., ionic bonds, hydrogen bonds and van der Waals interactions) characterized by |*V*_BCP_|/*G*_BCP_ < 1 (∇^2^*ρ*_BCP_ > 0 and *H*_BCP_ > 0); (b) pure open-shell (covalent) interactions characterized by |*V*_BCP_|/*G*_BCP_ > 2 (∇^2^*ρ*_BCP_ < 0 and *H*_BCP_ < 0) and (c) intermediate bonds with 1 < |*V*_BCP_|/*G*_BCP_ < 2 (i.e., ∇^2^*ρ*_BCP_ > 0 and *H*_BCP_ < 0) where *V*_BCP_ is the potential energy density at the BCP, *G*_BCP_ is the kinetic energy density at the BCP, ∇^2^*ρ*_BCP_ is the Laplacian of electron density *ρ* at the BCP and finally *H*_BCP_ is the energy density at the BCP. Accordingly, the calculated values of these properties for the BCP found between Pt and CO_2_ in intermediates **1_ImC**–**4_ImC** are given in Table 2.

Inspection of Table 1 reveals that the AIM parameters calculated for the Pt–CO_2_ BCP do not vary significantly between the **ImC** intermediates of **1**–**4**. Based upon the values of AIM parameters given in Table 1, we can assume that Pt–CO_2_ interaction falls into the third category, i.e., intermediate nature arising from an interplay of covalent, electrostatic, charge transfer and probably weak dispersion force components. However, the latter should be excluded for the Pt–CO_2_ bond, in terms of the Reduced Density Gradient (RDG) defined using the following equation [59]:

RDG(r) = |∇*ρ*(r)|/2(3π^2^)^1/3^*ρ*(r)^4/3^ (5)

where *ρ*(r) is the electron density *ρ* at point r. The 3D isosurface map of the RDG, depicted in Figure 5d, reveals the absence of any weak interactions between the Pt metal center and CO_2_ (notice that the green isosurface represents weak interactions regions). Similar RDG maps are observed for complexes **2**–**4** as well. In addition, inspection of Figure 5c reveals the existence of two BCPs located between the O atoms of CO_2_ and the H atoms of the N^C^N pincer ligand. Also, the RDG function (Figure 5d) reveals non-covalent interactions between those atoms. Therefore, these H bond interactions further contribute to the overall interaction of CO_2_ with the **Im_B** intermediate.

Another useful method for analyzing the chemical bond is the NBO method, which is a partitioning scheme for the electronic charge distribution in a molecule. Thus, we employed NBO calculations to analyze the Pt–CO_2_ bond from this point of view. The results of the NBO analysis are given in Table 3.

**Figure 6:**
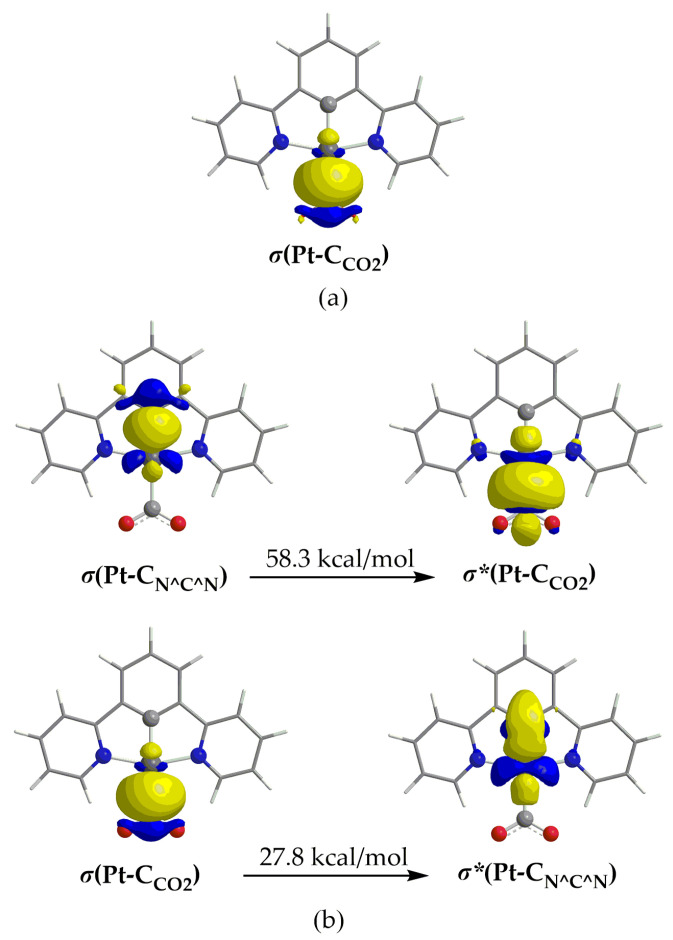
3D isosurface plots of (**a**) *σ*(Pt–C) BD NBO and (**b**) of donor and acceptor NBOs participating in the Pt–C hyperconjugative interactions.

The natural charges obtained using NBO analysis are found to be 0.235–0.260 for Pt and 0.494–0.497 for the C atom of the CO_2_ ligand. This results in a repulsive electrostatic interaction between the Pt and CO_2_ in line with previous findings [53,60]. The NBO analysis reveals also the existence of a bonding BD *σ* NBO, which is depicted in Figure 6a for **1_ImC** (similar BD NBOs are observed also for the rest of the respective intermediates).

The linear combinations of the BD[*σ*(Pt–CO_2_)] NBOs found for **1_ImC**–**4_ImC** are given in Table 2. Thus, for example, the *σ*(Pt–C) bonding NBO of **1_ImC** has an occupation number equal to 1.857 |e|, arising from the interaction of the sp^2.23^d^1.08^ hybrid orbital of Pt (23.15% s, 51.66% p and 25.03% d character) with the sp^1.88^ hybrid orbital of the C donor atom of CO_2_ (34.72% s and 65.25% p character), and is described as *σ*(Pt–C) = 0.558*h*_Pt_ + 0.830*h*_C_. In Figure 6b are also depicted the shapes of the NBOs participating in the donor– acceptor hyperconjugative interactions related to the Pt–CO_2_ bond in **1_ImC**. According to NBO analysis [61], there is a stabilization of a system due to the charge transfer interactions between specific donor-acceptor NBOs. The stabilization energy, Δ*E*(2), arising from these hyperconjugative (stereoelectronic) interactions is given using the following equation:

Δ*E*(2) = *q*_i_*F*_ij_/(*ε*_i_ − *ε*_j_) (6)

where *q*_i_ is the occupancy of the donor NBO, *F*_ij_ is the off-diagonal Fock matrix elements and *ε*_i_ and *ε*_j_ are the energy of the donor and acceptor NBOs, respectively. The two hyperconjugative interactions related to the Pt–CO_2_ bond given in Figure 6b involve *σ*(Pt–C) and σ*(Pt–C) NBOs and stabilize the system overall by 86.1 kcal/mol. The overall stabilization due to hyperconjugative interactions for **2_ImC**, **3_ImC** and **4_ImC** is 84.5, 85.5 and 80.5 kcal/mol, respectively.

**Scheme 3:**
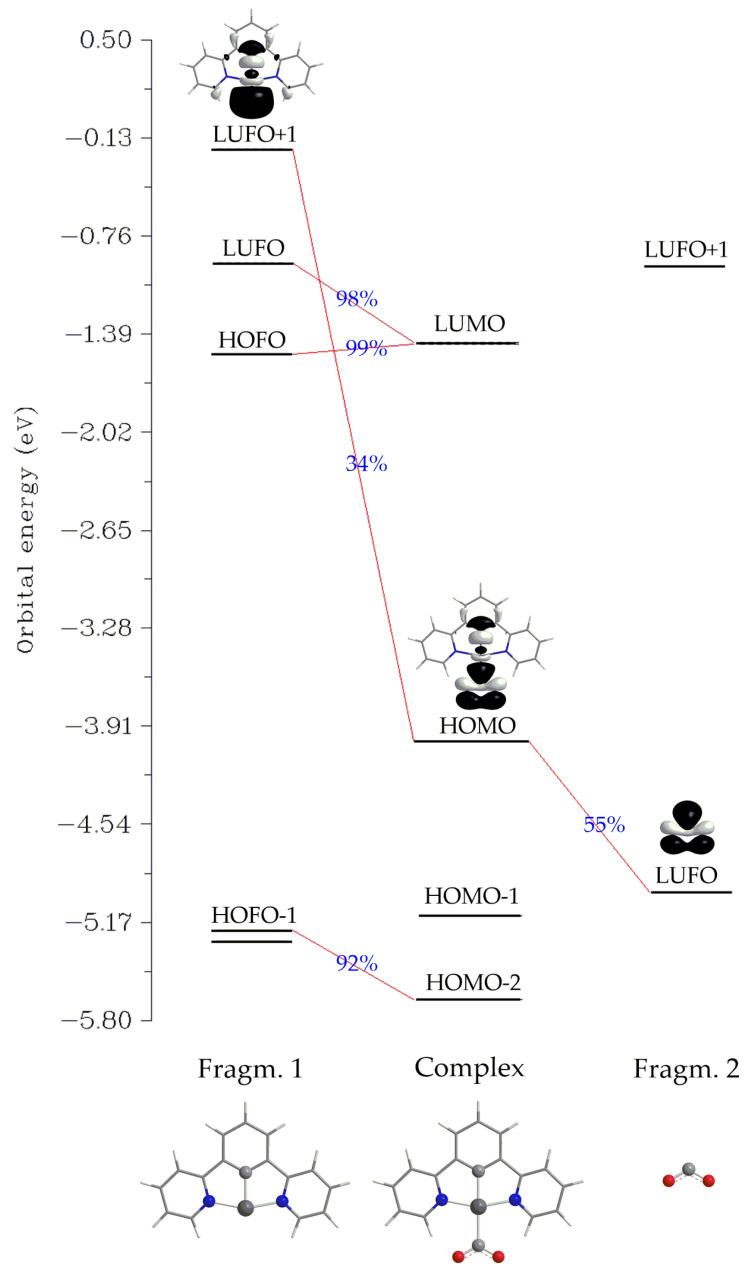
Orbital interaction diagram for the formation of **1_ImC** (Complex) from fragments [Pt(dpb)]^−^ (Fragm. 1) and CO_2_ (Fragm. 2).

Another method for analyzing a chemical bond is Charge Decomposition Analysis (CDA) [62]. We employed the latter to study the Pt–CO_2_ interaction for **1_ImC** as a representative case. In contrast to the Co–CO_2_ interaction in Co complexes, where charge transfer has been proposed [63,64], the net charge transfer from the Pt fragment to the CO_2_ is calculated to be marginal, amounting to only 0.048 |e| (the donation *d* and backdonation *b* terms were found to be −0.023 and −0.071 |e|, respectively, and are somewhat balanced). Finally, the charge polarization term *r* has a negative value equal to −0.350, indicating electronic charge removal from the overlapping into the non-overlapping regions upon the formation of **1_ImC**. An orbital interaction diagram for the formation of **1_ImC** from the fragments [Pt(dpb)]^−^ (Fragm. 1) and CO_2_ (Fragm. 2) is depicted in Scheme 3.

Perusal of Scheme 3 reveals that the bonding HOMO of the complex **1_ImC**, reflecting the covalent Pt-CO_2_ interaction, is composed of 34% of LUFO+1 of Fragm. 1 and 55% of LUFO of Fragm. 2. In other words, the HOMO of **1_ImC** is constructed by the in-phase interaction of the, mainly of the Pt d_z2_ character, LUFO+1 of the [Pt(dpb)]^−^ fragment, with a π* MO of the CO_2_ fragment.

#### 2.2.2. Energetic Reaction Profiles

**Scheme 4:**
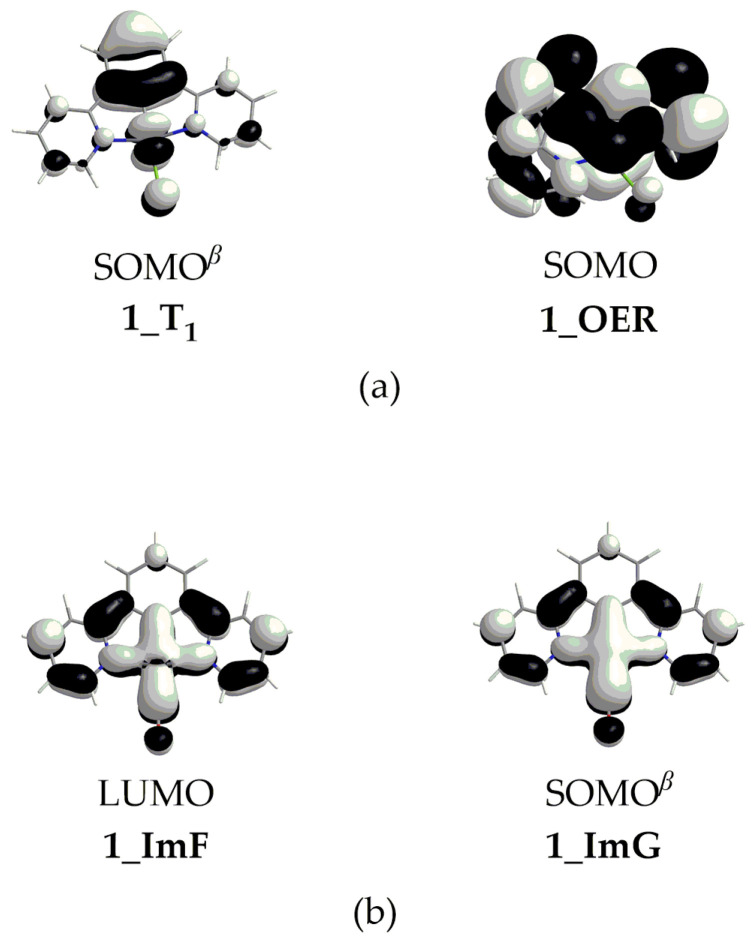
(**a**) SOMO*^β^* of **1_T_1_** species (**left**) and SOMO of **1_OER** (**right**) and (**b**) LUMO of **1_ImF** (**left**) and SOMO*^β^* of **1_G** (**right**).

**Figure 7:**
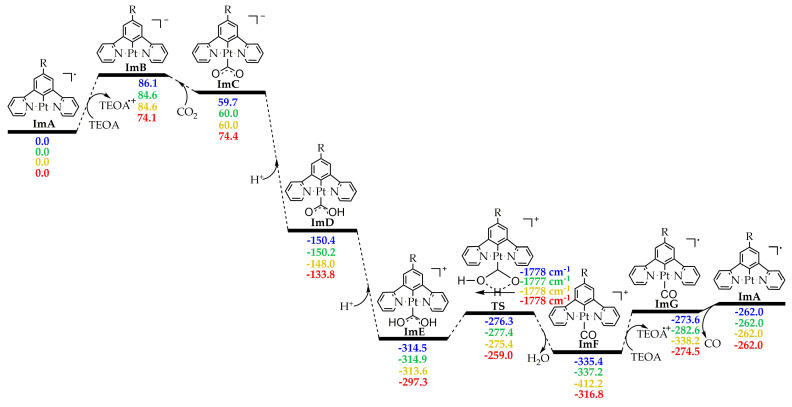
Free energy, Δ*G* (in kcal/mol), reaction profiles of **1**–**4** in DCM solvent calculated at the PBE0-GD3BJ/Def2-TZVP level (numbers in blue for **1**, in green for **2**, in orange for **3** and in red for **4**).

The free energy reaction profiles calculated for **1**–**4**, corresponding to the respective catalytic cycle step (Figure 1), are given in Figure 7. The catalytic cycle starts with **ImA**, which is obtained from **OER** upon the loss of the chloride ligand (vide supra). Experimental studies have already proposed the loss of the halide ligand from the Re and Ru complexes used in catalytic CO_2_ conversion [2]. Our study indicates that this should also hold for the Pt complexes under investigation. Therefore, the latter, upon reduction in their T_1_ excited state, accept an electron in the SOMO*^β^* depicted in Scheme 4. Putting an electron in this antibonding MO would weaken the Pt–Cl bond, thus facilitating its dissociation. This is further corroborated by the fact that the single electron in the OER species resides on the antibonding SOMO (Scheme 4), destabilizing the Pt–Cl bond and favoring its dissociation.

Next, inspection of the energetic profiles reveals that the CO_2_-into-CO conversion by the catalytically active species **ImA** is a strongly exergonic reaction. Upon reduction, **ImA** yields the reactive intermediate **ImB**, which subsequently captures a CO_2_ molecule to yield the *η*^1^–CO_2_ complex **ImC**. Successive protonation of the latter results in the formation of the immensely stabilized intermediate **ImE**. Next, **ImE** is converted into the carbonyl intermediate **ImF** through a concerted mechanism and via a transition state, **TS**, surmounting an activation barrier of around 38 kcal/mol. Finally, upon a second electron reduction, we obtain CO while regenerating the catalytically active species **ImA**.

It should be noticed that the dissociation of the Pt–CO upon the reduction of the **ImF** intermediate should be favorable since it accepts an electron in its antibonding LUMO (Scheme 4). Also, the single electron in the **ImG** intermediate resides on the antibonding SOMO*^β^* (Scheme 4), destabilizing the Pt–CO bond. These findings are in line with experimental studies proposing CO dissociation upon the reduction of various complexes acting as CO_2_ reduction catalysts [2].

The optimized geometries, along with selected structural parameters, of all the intermediates and TSs involved in the catalytic cycle are given in Figures S7–S10. Perusal of the latter reveals that the substitution of one H atom of **1** by an R substituent (Scheme 1) has no significant impact on the structural parameters of the [Pt(5-R-dpb)Cl] complexes.

## 3. Computational Methods

Full geometry optimization has been performed for all the species under study, without symmetry constraints, using the 1997 hybrid functional of Perdew, Burke and Ernzerhof [65,66,67,68,69,70], as implemented in the program Gaussian16W [71]. This functional uses 25% exchange and 75% weighting correlation and is denoted as PBE0. Dispersion interactions were accounted for by using the D3 version of Grimme dispersion with Becke−Johnson damping [72]. The Def2-TZVP basis set for all atoms was used for the geometry optimizations. The computational protocol will be hereafter denoted as PBE0-GD3BJ/Def2-TZVP. All stationary points have been identified as minima (number of imaginary frequencies, NImag = 0). Natural bond orbital (NBO) population analysis was carried out, employing the methodology by Weinhold [63] as implemented in the G16W software, version C.01. The atoms in molecules (AIM) method of Bader [57], the reduced gradient density (RDG) method [73] and the CDA method [62] were used as implemented in the Multiwfn software, version 3.8 [74]. The Gibbs free energy was calculated to be 298.15 K and 1 atm pressure. Solvent effects were calculated using the Polarizable Continuum Model (PCM) using the integral equation formalism variant (IEF-PCM), which is the default method of G16W (self-consistent reaction field (SCRF)) [75], while Dichloromethane (DCM) was used as the solvent. Time-dependent density functional theory (TD-DFT) calculations [76,77,78] were performed on the ground-state S_0_ equilibrium geometries in the DCM solvent employing two computational protocols, namely PBE0/Def2-TZVP and CAM-B3LYP/Def2-TZVP, taking into account the first 30 excited states. Both PBE0 and CAM-B3LYP are among the most popular functionals for the calculation of absorption spectra [79]. Also, the def2-TZVP basis set has been successfully employed for the calculation of the absorption spectra of the Pt(II) square planar complexes [80]. Finally, Natural Transition Orbitals were used, offering a greatly simplified description of the electronic transitions. [81].

## 4. Conclusions

The photocatalytic reduction of CO_2_ into CO by [Pt(5-R-dpb)Cl] (dpb = 1,3-di(2-pyridyl)benzene anion, R = H, *N*,*N*-dimethylaniline, thiophene, bodipy) Pt(II) square planar complexes has been studied using DFT electronic structure calculations. The overall process is thought to proceed via two main steps: (i) photoexcitation/reduction of the initial complexes and (ii) the main catalytic cycle, producing CO. The TDDFT-simulated absorption spectra showed that these complexes exhibit strong absorptions in the range of 280–380 of the UV region, as well as weak absorptions in the range of 410–460 nm. The exception is complex **4**, with a diazaborinine-substituted N^C^N pincer ligand, where this phenomenon is reversed, with the strongest absorption appearing around 420 nm and the weakest around 280 nm. Thus, it is anticipated that changing the pincer ligand could provide complexes that absorb in the visible region, making them ideal for use as photocatalysts for CO_2_-into-CO conversion. Next, the excited state reduction potentials, *E*^0*^_red_, dictating the reductive quenching of the Pt(II) complexes in their T_1_ state have been calculated using DFT. Based upon the *E*^0*^_red_ values, we conclude that the oxidizing ability of the Pt(II) complexes in their T_1_ state follows the series **2** > **1** > **3** > **4**, meaning that **2** is expected to be more easily reduced amongst all the complexes under study. In general, based upon the calculated redox potentials *E*^0*^_redox_ in the range of 6–8 V, all the complexes are expected to be easily reduced with a sacrificial electron donor such as TEOA or TEA. During reduction, the complexes **1**–**4** in the T_1_ state accept an e^−^ in their SOMO, being antibonding in the region of the Pt–Cl bond. In the **OER** product species, resulting from the reduction of the T_1_ state complexes, the single e^−^ resides also in an antibonding SOMO, destabilizing and facilitating the Pt–Cl bond dissociation process.

The second step of photocatalytic CO_2_-into-CO conversion, namely the catalytic cycle, starts with a three-coordinated Pt(II) **ImA** intermediate, obtained from **OER** upon Pt–Cl dissociation. Upon one-e^−^ reduction, ImA captures CO_2_, and after successive protonations and reductions, yields CO. The latter is obtained from **ImF**, which accepts an e^−^ in its LUMO, being antibonding in the region of the Pt–CO bond, while this e^−^ resides in a similar antibonding SOMO of the reduced **ImG** intermediate. Consequently, the Pt–CO bond is expected to be broken in the final stage of the catalytic cycle.

The Pt–CO_2_ bond of the **ImC** intermediate, crucial to the overall process, has been scrutinized. It is revealed that this bond is relatively strong with *D*_0_ in the range of −36.9–−10.3 kcal/mol, exhibiting a complex nature comprising mainly covalent and hyperconjugative interactions, which compensate for the repulsive electrostatic interactions. The Pt–CO_2_ bond involves also H bonding with the N^C^N pincer ligand. Overall, the catalytic cycle is estimated to be a strongly exergonic process. Taking into account that **1** exhibits a very high T_1_ excited state lifetime *τ*, a whole new series of Pt(II) complexes could be synthesized bearing suitable pincer ligands in order to absorb in the visible range, making them ideal for CO_2_-into-CO conversion using sunlight.

## Figures and Tables

**Table 1:** Reduction potentials (in Volts) of **1**–**4** in S_0_ state, *E*^0^_red_, in T_1_ state, *E*^0*^_red_, along with *E*^0^_red_ of TEOA and TEA and the respective values vs. SHE calculated at the PBE0/Def2-TZVP level.

Complex	*E* ^0^_red_	*E* ^0*^_red_	*E*^0^_red_ vs. SHE	*E*^0*^_red_ vs. SHE ^1^	*E*^0*^_redox_ ^2^	
					vs. TEOA	vs. TEA
**1**	−2.48	1.80	0.32	4.60	7.43	7.19
**2**	−2.46	1.82	−0.36	3.92	7.45	7.21
**3**	−2.54	1.74	−0.34	3.94	7.37	7.13
**4**	−3.48	0.80	−1.88	2.40	6.43	6.19
TEOA	−5.63		−1.35			
TEA	−5.39		−1.11			

^1^ SHE = Standard Hydrogen Electrode. ^2^ Taken as *E*^0^_red_(complex) + *E*^0^_ox_(*D*) where *E*^0^_ox_(*D*) = −*E*^0^_red_(*D*).

**Table 2:** Topological and energetic properties of *ρ*(r) calculated at the (3, −1) bond-critical point (BCP) located at the Pt-CO_2_ bond of intermediates **1_ImC**–**4_ImC**.

Species	*ρ*_BCP_ ^1^	∇*ρ*_BCP_ ^2^	*G*_BCP_ ^3^	*V*_BCP_ ^3^	|*V*_BCP_|/*G*_BCP_	*H*_BCP_ ^3^	*G*_BCP_/*ρ*_BCP_ ^4^
**1_ImC**	0.124	0.137	0.089	−0.145	1.629	−0.056	0.718
**2_ImC**	0.125	0.138	0.090	−0.147	1.633	−0.057	0.720
**3_ImC**	0.125	0.135	0.090	−0.147	1.633	−0.057	0.720
**4_ImC**	0.125	0.130	0.088	−0.145	1.648	−0.057	0.704

^1^ in eÅ^−3^. ^2^ in eÅ^−5^. ^3^ in kJ mol^−1^ (atomic unit volume)^−1^. ^4^ in kJ mol^−1^ electron^−1^. The bond degree parameter *H*_BCP_/*ρ*_BCP_ represents either the covalence (*H*_BCP_ < 0) or the softening (*H*_BCP_ > 0) degree of the interaction.

**Table 3:** Natural charges *Q*, bonding NBOs *BD* and hyperconjugative interaction stabilization energy Δ*E*(2) relevant to the Pt–CO_2_ in **1_ImC**–**4_ImC**.

Species	*Q*_Pt_	*Q*_C_ ^1^	BD[*σ*(Pt-CO_2_)]	Δ*E*(2)	
				*σ*(Pt-C_N^C^N_) → σ*(Pt-CO_2_)	*σ*(Pt-CO_2_) → σ*(Pt-C_N^C^N_)
**1_ImC**	0.235	0.495	0.558*h*_Pt_ + 0.830*h*_C_	58.3	27.8
**2_ImC**	0.237	0.494	0.540*h*_Pt_ + 0.842*h*_C_	58.1	26.4
**3_ImC**	0.244	0.495	0.538*h*_Pt_ + 0.843*h*_C_	58.3	27.2
**4_ImC**	0.260	0.497	0.535*h*_Pt_ + 0.845*h*_C_	56.9	23.6

^1^ Referring to the CO_2_ carbon atom.

## Data Availability

The data presented in this study are available in article and Supplementary Materials.

## Supplementary Materials

The following supporting information can be downloaded at: https://www.mdpi.com/article/10.3390/molecules29020403/s1, Figure S1: Optimized geometries of **2** in S_0_ ground state **2_S_0_**, in T_1_ excited state **2_T_1_** and of One-Electron-Reduced form **2_OER**, calculated at the PBE0-GD3BJ/Def2-TZVP levels in DCM solvent. Figure S2: Optimized geometries of **3** in S_0_ ground state **3_S_0_**, in T_1_ excited state **3_T_1_** and of One-Electron-Reduced form **3_OER**, calculated at the PBE0-GD3BJ/Def2-TZVP levels in DCM solvent. Figure S3: Optimized geometries of **4** in S_0_ ground state **4_S_0_**, in T_1_ excited state **4_T_1_** and of One-Electron-Reduced form **4_OER**, calculated at the PBE0-GD3BJ/Def2-TZVP levels in DCM solvent. Figure S4: Simulated absorption spectra of **1**–**4** in DCM at the TDDFT/CAM-B3LYP/Def2-TZVP levels. Figure S5: NTO pairs for the most significant electronic transitions of the simulated absorption spectra of **2** calculated at TDDFT/PBE0/Def2-TZVP levels (hole *h*^+^ on the left, electron *e*^−^ on the right). Figure S6: NTO pairs for the most significant electronic transitions of the simulated absorption spectra of **3** calculated at TDDFT/PBE0/Def2-TZVP levels (hole *h*^+^ on the left, electron *e*^−^ on the right). Figure S7: NTO pairs for the most significant electronic transitions of the simulated absorption spectra of **4** calculated at TDDFT/PBE0/Def2-TZVP levels (hole *h*^+^ on the left, electron *e*^−^ on the right). Figure S8: Optimized geometries of all species involved in the catalytic cycle of CO_2_-into-CO conversion by **1** calculated at the PBE0-GD3BJ/Def2-TZVP levels in DCM solvent. Figure S9: Optimized geometries of all species involved in the catalytic cycle of CO_2_-into-CO conversion by **2** calculated at the PBE0-GD3BJ/Def2-TZVP levels in DCM solvent. Figure S10: Optimized geometries of all species involved in the catalytic cycle of CO_2_-into-CO conversion by **3** calculated at the PBE0-GD3BJ/Def2-TZVP levels in DCM solvent. Figure S11: Optimized geometries of all species involved in the catalytic cycle of CO_2_-into-CO conversion by **4** calculated at the PBE0-GD3BJ/Def2-TZVP levels in DCM solvent. Table S1: Cartesian coordinates and energetic data of the optimized geometries of all species calculated at the PBE0-GD3BJ/Def2-TZVP levels in DCM solvent.
