# Supplementary material for: Photocatalytic Reduction of CO2 into CO with Cyclometalated Pt(II) Complexes of N^C^N Pincer Dipyridylbenzene Ligands: A DFT Study
Source: Molecules. 2024 Jan 14;29(2):403. doi: 10.3390/molecules29020403 (PMC10820273; doi:10.3390/molecules29020403)
Supplement: Supplementary file 1 [file molecules-29-00403-s001.zip › molecules-2815125-supplementary.pdf]

**Table of Contents**

*Figure S1*: Optimized geometries of **2** in $S_0$ ground state, **2_S$_0$** in $T_1$ excited state, **2_T$_1$** and of One Electron Reduced form, **2_OER**, calculated at the PBE0-GD3BJ/Def2-TZVP level, in DCM solvent. 2

*Figure S2*: Optimized geometries of **3** in $S_0$ ground state, **3_S$_0$** in $T_1$ excited state, **3_T$_1$** and of One Electron Reduced form, **3_OER**, calculated at the PBE0-GD3BJ/Def2-TZVP level, in DCM solvent. 3

*Figure S3*: Optimized geometries of **4** in $S_0$ ground state, **4_S$_0$** in $T_1$ excited state, **4_T$_1$** and of One Electron Reduced form, **4_OER**, calculated at the PBE0-GD3BJ/Def2-TZVP level, in DCM solvent. 4

*Figure S4*: Simulated absorption spectra of **1 – 4** in DCM at the TDDFT/CAM-B3LYP/Def2-TZVP level. 5

*Figure S5*: NTO pairs for the most significant electronic transitions of the simulated absorption spectra of **2** calculated at TDDFT/PBE0/Def2-TZVP level (hole, $h^+$ at the left, electron, $e^-$ at the right). 6

*Figure S6*: NTO pairs for the most significant electronic transitions of the simulated absorption spectra of **3** calculated at TDDFT/PBE0/Def2-TZVP level (hole, $h^+$ at the left, electron, $e^-$ at the right). 7

*Figure S7*: NTO pairs for the most significant electronic transitions of the simulated absorption spectra of **4** calculated at TDDFT/PBE0/Def2-TZVP level (hole, $h^+$ at the left, electron, $e^-$ at the right). 8

*Figure S8*: Optimized geometries of all species involved in the catalytic cycle of $CO_2$ to CO conversion by **1** calculated at the PBE0-GD3BJ/Def2-TZVP level, in DCM solvent. 9

*Figure S9*: Optimized geometries of all species involved in the catalytic cycle of $CO_2$ to CO conversion by **2** calculated at the PBE0-GD3BJ/Def2-TZVP level, in DCM solvent. 10

*Figure S10*: Optimized geometries of all species involved in the catalytic cycle of $CO_2$ to CO conversion by **3** calculated at the PBE0-GD3BJ/Def2-TZVP level, in DCM solvent. 11

*Figure S11*: Optimized geometries of all species involved in the catalytic cycle of $CO_2$ to CO conversion by **4** calculated at the PBE0-GD3BJ/Def2-TZVP level, in DCM solvent. 12

*Table S1*: Cartesian Coordinates and Energetic Data of the optimized geometries of all species calculated at the PBE0-GD3BJ/Def2-TZVP level, in DCM solvent. 13

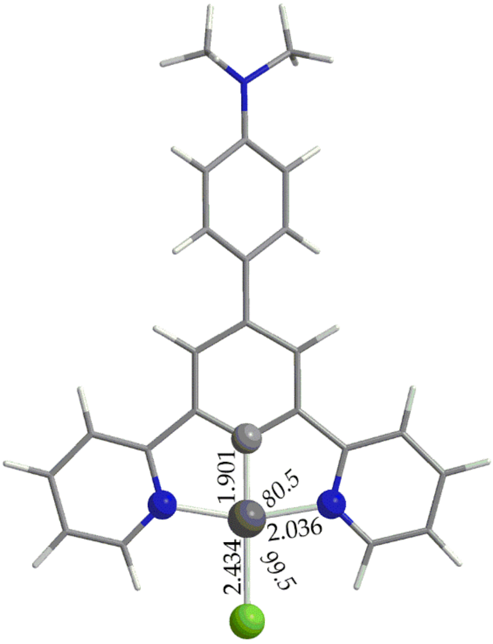


**2_S$_0$**

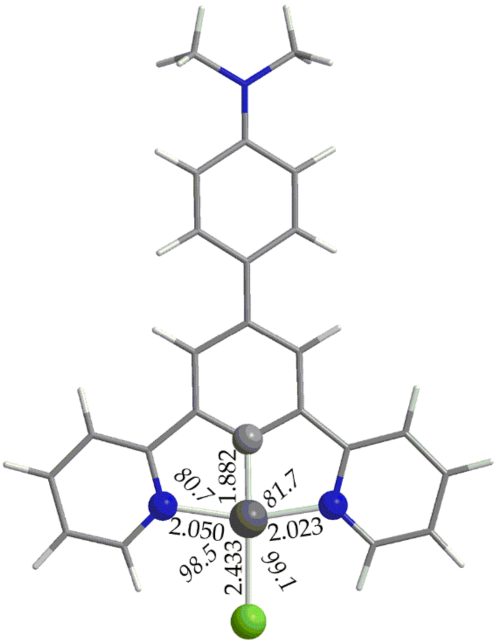


**2_T$_1$**

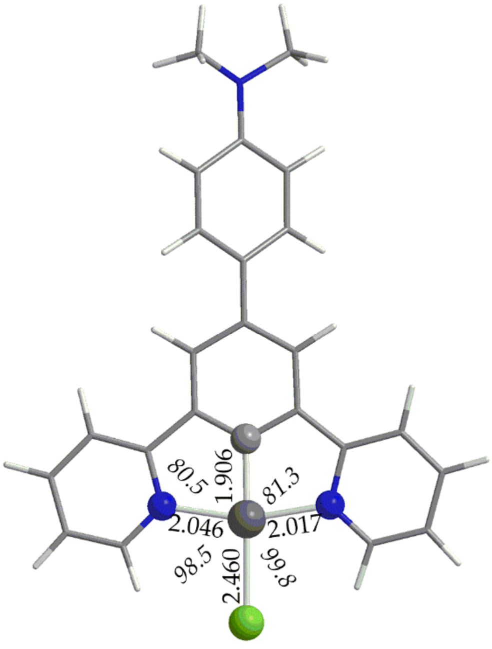


**2_OER**

*Figure S1*: Optimized geometries of **2** in $S_0$ ground state, **2_S$_0$** in $T_1$ excited state, **2_T$_1$** and of One Electron Reduced form, **2_OER**, calculated at the PBE0-GD3BJ/Def2-TZVP level, in DCM solvent.

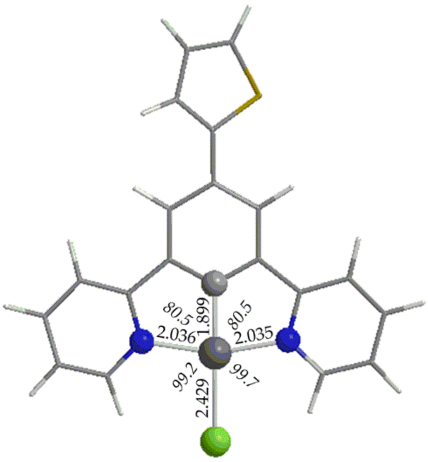


**3_S$_0$**

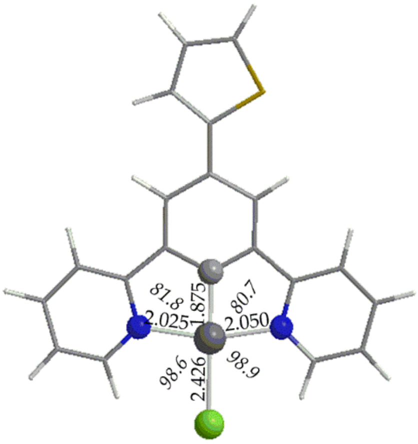


**3_T$_1$**

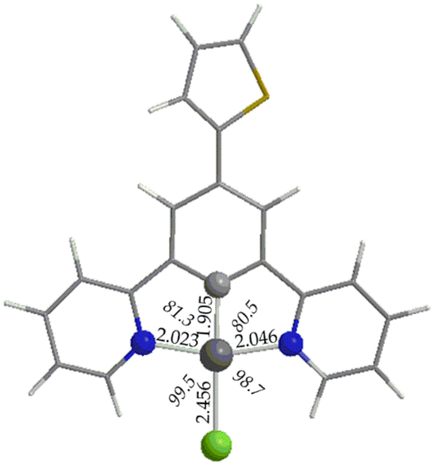


**3_OER**

*Figure S2*: Optimized geometries of **3** in $S_0$ ground state, **3_S$_0$** in $T_1$ excited state, **3_T$_1$** and of One Electron Reduced form, **3_OER**, calculated at the PBE0-GD3BJ/Def2-TZVP level, in DCM solvent.

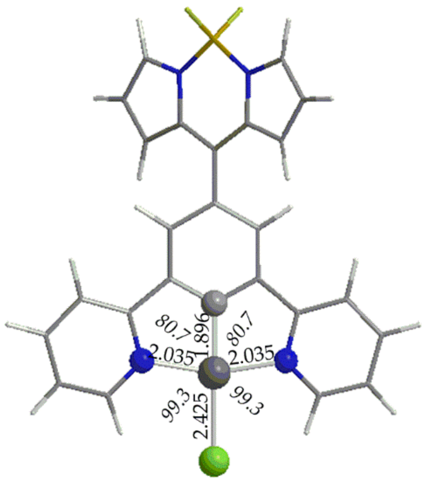


**4_S$_0$**

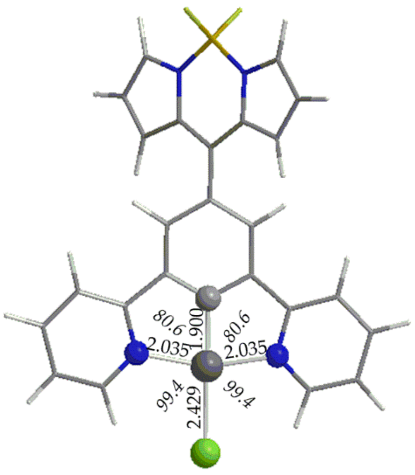


**4_T$_1$**

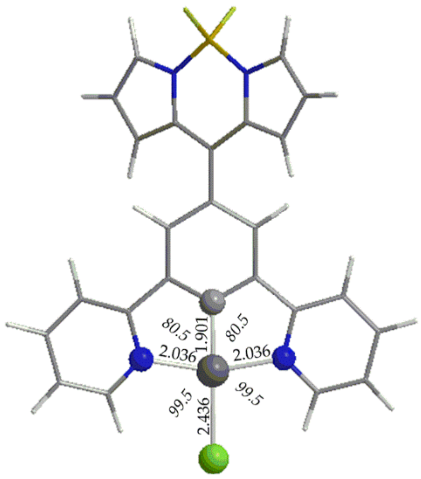


**4_OER**

*Figure S3*: Optimized geometries of **4** in $S_0$ ground state, **4_S$_0$** in $T_1$ excited state, **4_T$_1$** and of One Electron Reduced form, **4_OER**, calculated at the PBE0-GD3BJ/Def2-TZVP level, in DCM solvent.

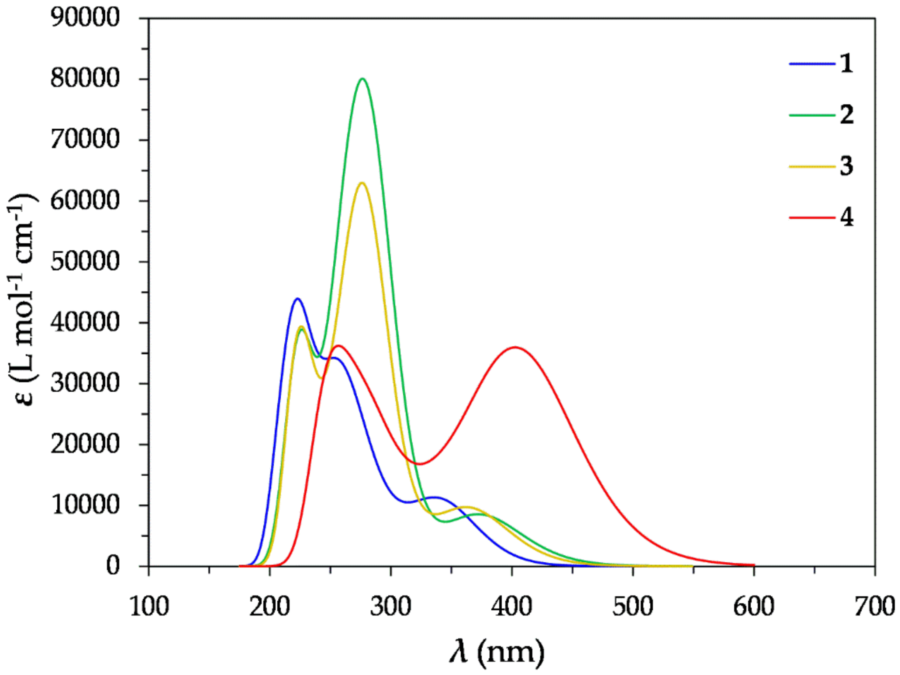


*Figure S4*: Simulated absorption spectra of **1 – 4** in DCM at the TDDFT/CAM-B3LYP/Def2-TZVP level.

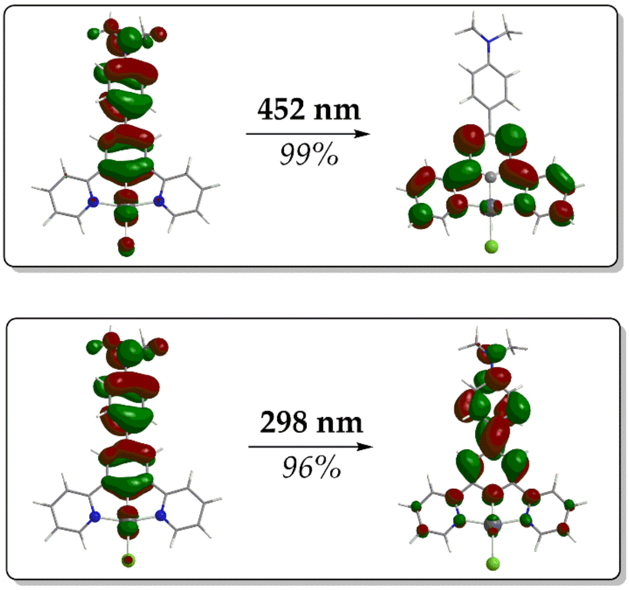


*Figure S5*: NTO pairs for the most significant electronic transitions of the simulated absorption spectra of **2** calculated at TDDFT/PBE0/Def2-TZVP level (hole, $h^+$ at the left, electron, $e^-$ at the right).

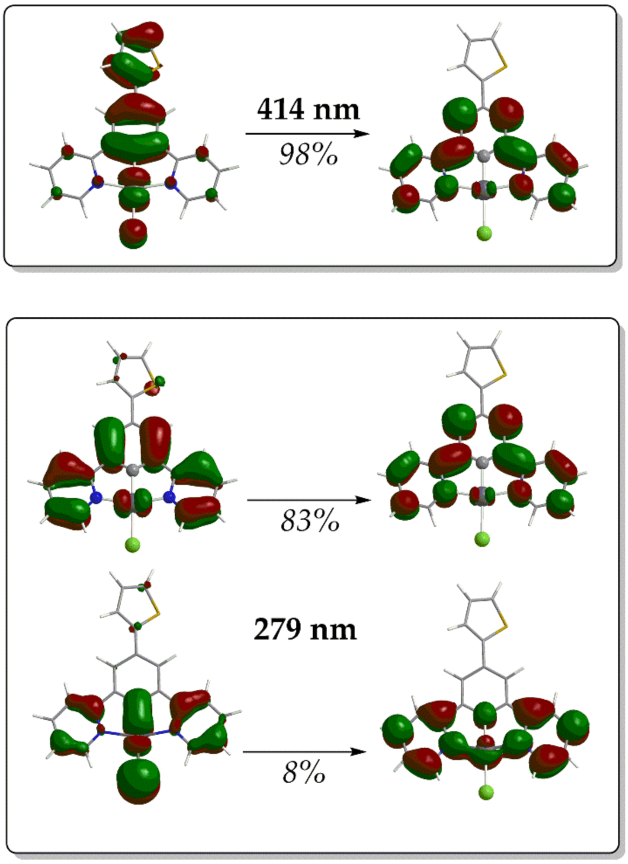


*Figure S6*: NTO pairs for the most significant electronic transitions of the simulated absorption spectra of **3** calculated at TDDFT/PBE0/Def2-TZVP level (hole, $h^+$ at the left, electron, $e^-$ at the right).

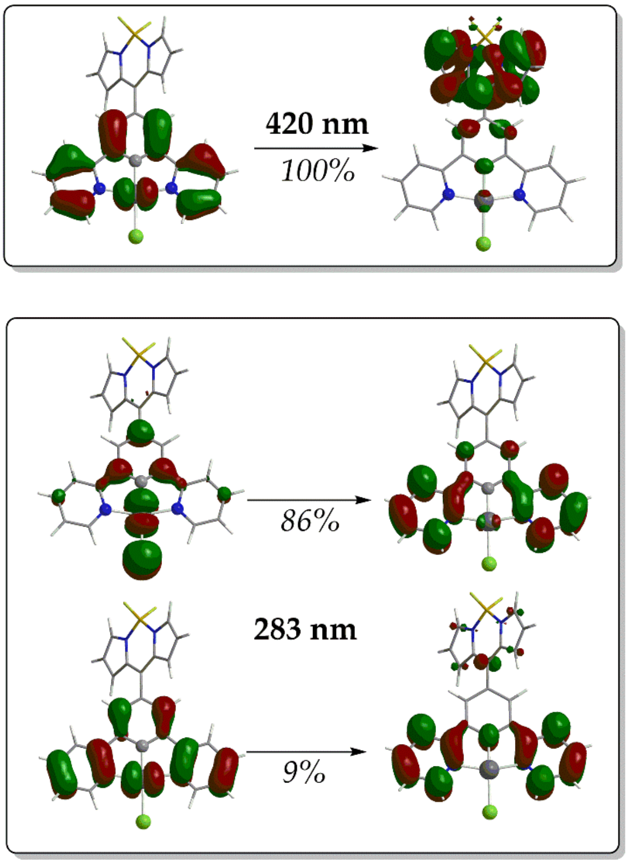


*Figure S7*: NTO pairs for the most significant electronic transitions of the simulated absorption spectra of **4** calculated at TDDFT/PBE0/Def2-TZVP level (hole, $h^{+}$ at the left, electron, $e^{-}$ at the right).

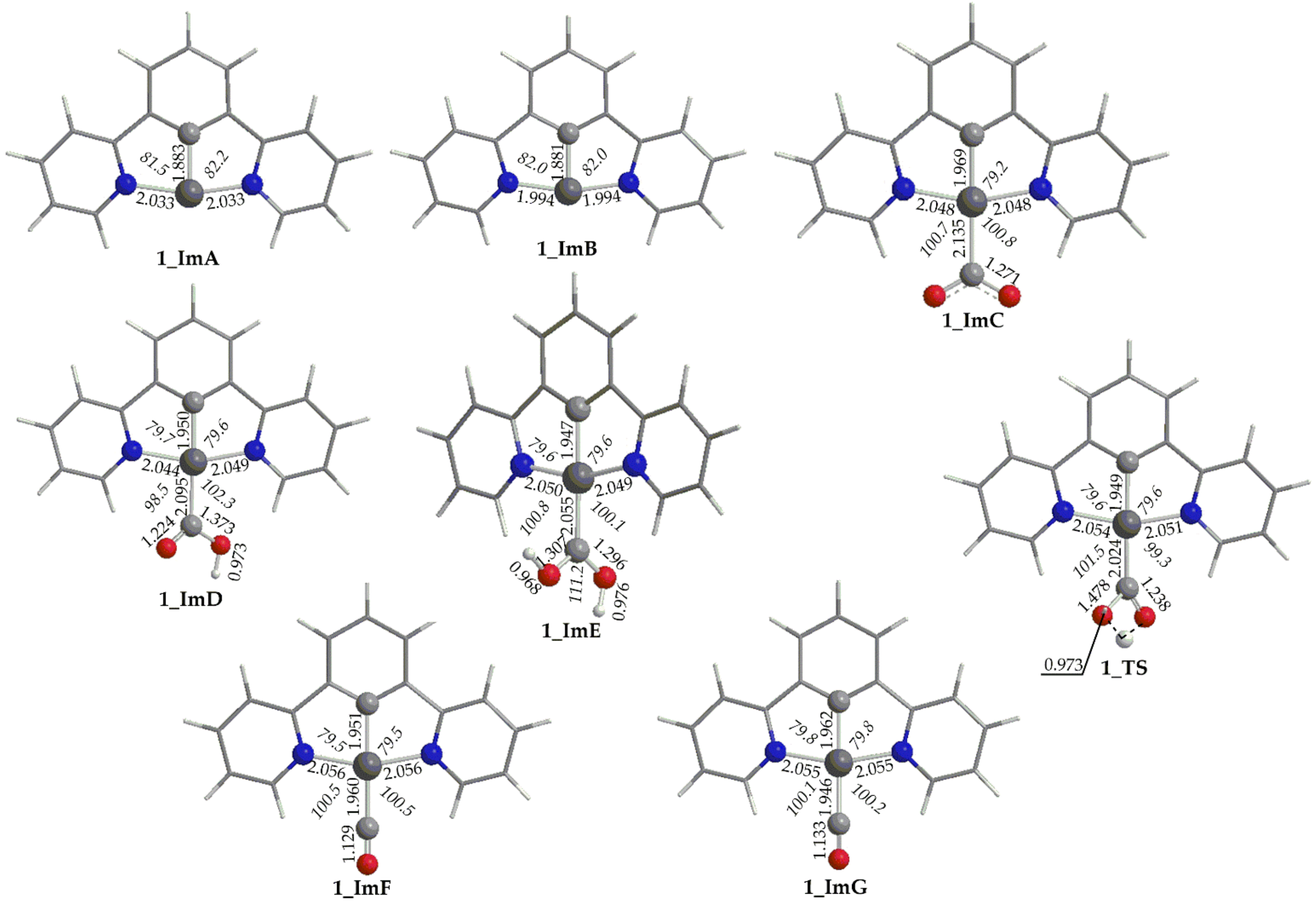


*Figure S8*: Optimized geometries of all species involved in the catalytic cycle of $CO_2$ to CO conversion by **1** calculated at the PBE0-GD3BJ/Def2-TZVP level, in DCM solvent.

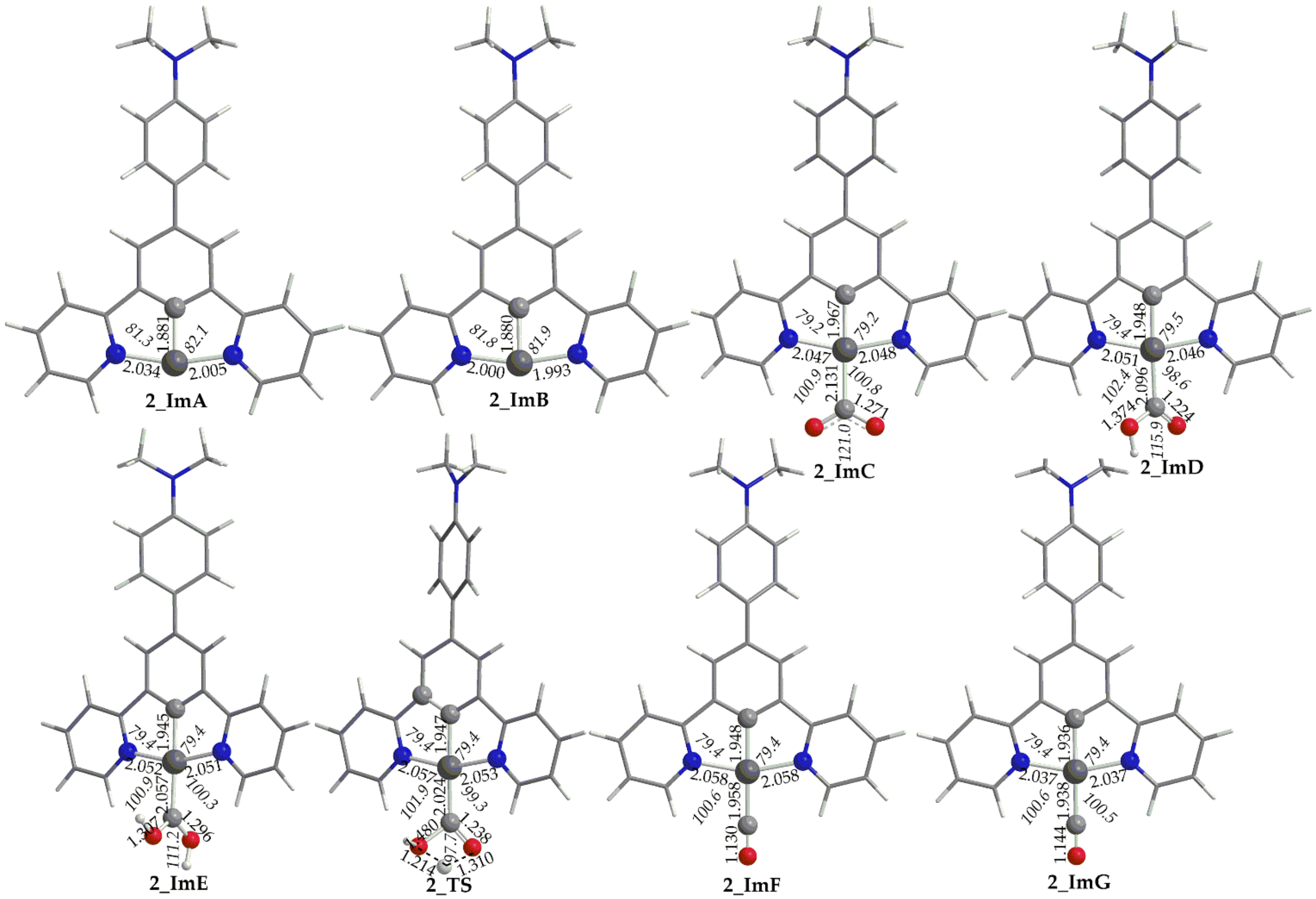


*Figure S9*: Optimized geometries of all species involved in the catalytic cycle of $CO_2$ to CO conversion by **2** calculated at the PBE0-GD3BJ/Def2-TZVP level, in DCM solvent.

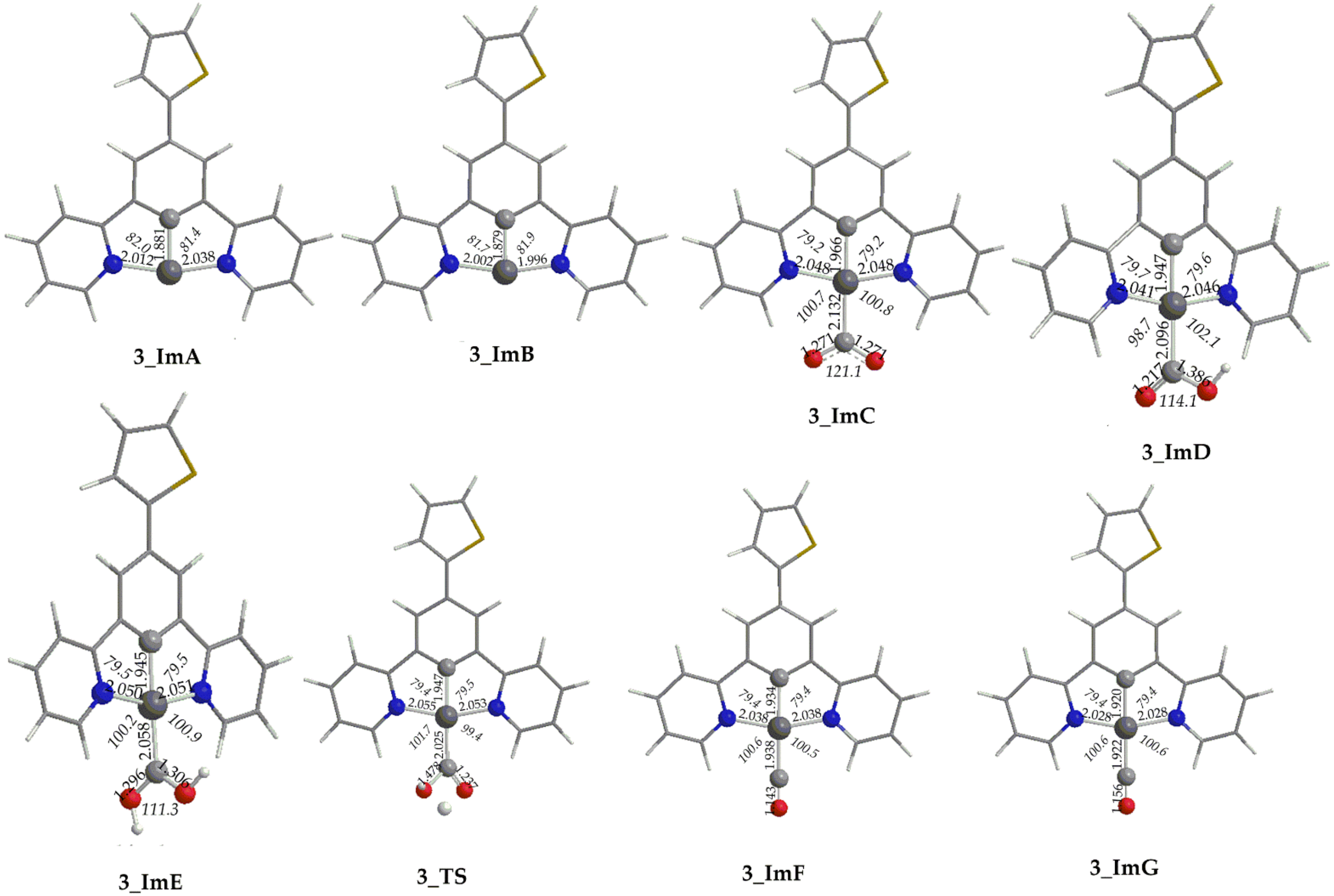


*Figure S10*: Optimized geometries of all species involved in the catalytic cycle of $CO_2$ to CO conversion by **3** calculated at the PBE0-GD3BJ/Def2-TZVP level, in DCM solvent.

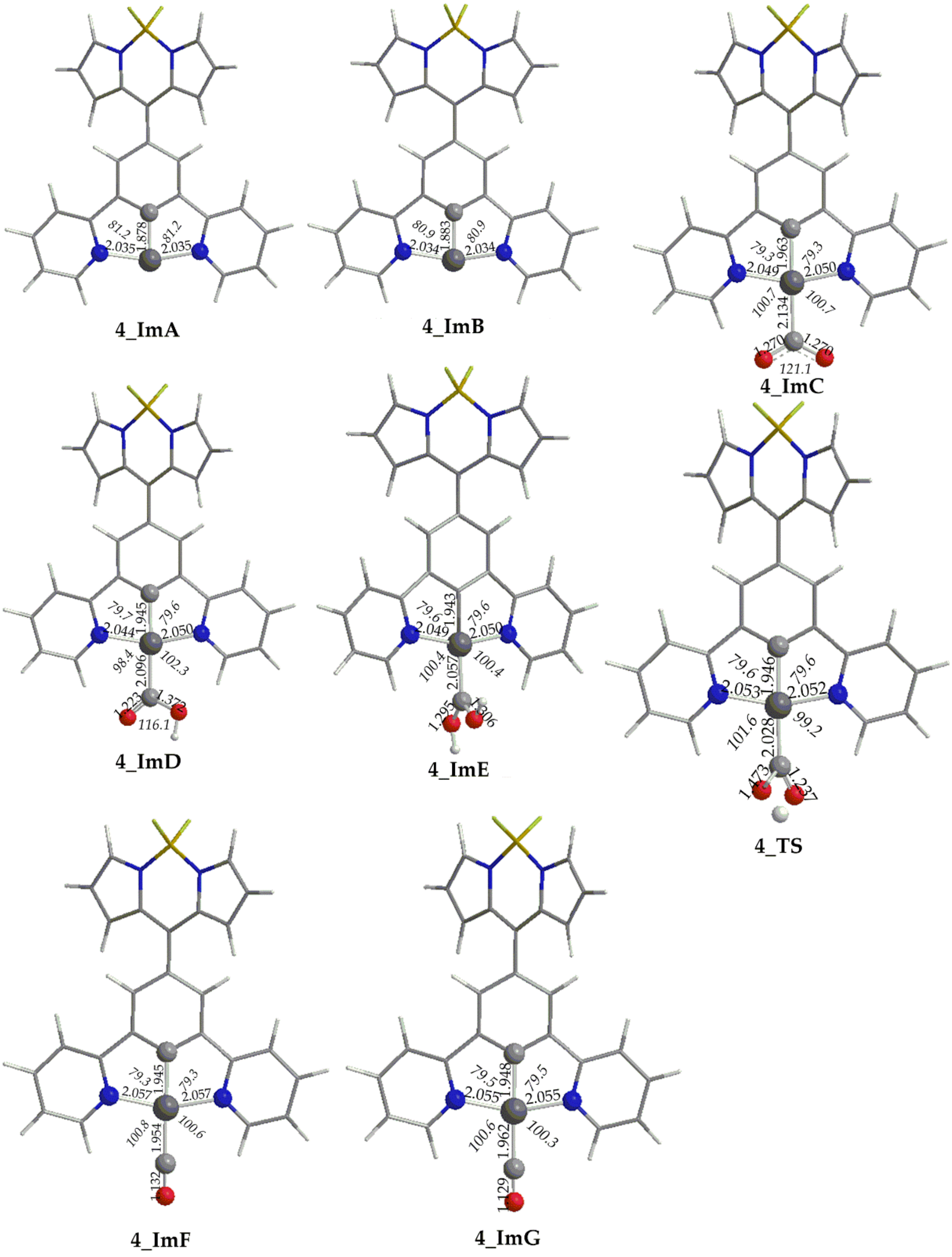


*Figure S11*: Optimized geometries of all species involved in the catalytic cycle of $CO_2$ to CO conversion by **4** calculated at the PBE0-GD3BJ/Def2-TZVP level, in DCM solvent.

*Table S1*: Cartesian Coordinates and Energetic Data of the optimized geometries of all species calculated at the PBE0-GD3BJ/Def2-TZVP level, in DCM solvent.

**1_S0**

| | | | |
|---|---|---|---|
| Pt | -0.000002000 | -0.670014000 | 0.000206000 |
| Cl | 0.000035000 | -3.103144000 | -0.000060000 |
| C | -1.216915000 | 3.293769000 | 0.000144000 |
| C | -1.222268000 | 1.900985000 | -0.000010000 |
| C | -0.000006000 | 1.232065000 | 0.000025000 |
| C | 1.222254000 | 1.900988000 | -0.000009000 |
| C | 1.216895000 | 3.293772000 | 0.000145000 |
| C | -0.000011000 | 3.972104000 | 0.000237000 |
| C | -2.357371000 | 0.983221000 | -0.000085000 |
| C | 2.357361000 | 0.983230000 | -0.000084000 |
| C | 3.695650000 | 1.345677000 | -0.000377000 |
| C | 4.674794000 | 0.368754000 | -0.000481000 |
| C | 4.300118000 | -0.965686000 | -0.000301000 |
| C | 2.954546000 | -1.277455000 | -0.000076000 |
| N | -2.007180000 | -0.338722000 | 0.000004000 |
| N | 2.007180000 | -0.338716000 | 0.000006000 |
| C | -2.954537000 | -1.277470000 | -0.000077000 |
| C | -4.300111000 | -0.965714000 | -0.000300000 |
| C | -4.674798000 | 0.368723000 | -0.000479000 |
| C | -3.695664000 | 1.345656000 | -0.000377000 |
| H | -2.141015000 | 3.861630000 | 0.000171000 |
| H | 2.140992000 | 3.861638000 | 0.000174000 |
| H | -0.000012000 | 5.055237000 | 0.000357000 |
| H | 3.960727000 | 2.394703000 | -0.000534000 |
| H | 5.721590000 | 0.647140000 | -0.000725000 |
| H | 5.032830000 | -1.761291000 | -0.000349000 |
| H | 2.595763000 | -2.299300000 | 0.000008000 |
| H | -2.595745000 | -2.299313000 | 0.000005000 |
| H | -5.032817000 | -1.761325000 | -0.000346000 |
| H | -5.721597000 | 0.647099000 | -0.000723000 |
| H | -3.960750000 | 2.394679000 | -0.000534000 |

Sum of electronic and zero-point Energies= -1304.604327
Sum of electronic and thermal Energies= -1304.588750
Sum of electronic and thermal Enthalpies= -1304.587806
Sum of electronic and thermal Free Energies= -1304.648026

**1_T1**

| | | | |
|---|---|---|---|
| Pt | 0.000000000 | 0.657338000 | 0.000047000 |
| Cl | 0.000002000 | 3.058244000 | -0.000277000 |
| C | -1.241109000 | -3.289794000 | -0.000018000 |
| C | -1.254378000 | -1.878162000 | -0.000001000 |
| C | 0.000000000 | -1.215806000 | 0.000032000 |
| C | 1.254371000 | -1.878163000 | 0.000024000 |
| C | 1.241105000 | -3.289791000 | 0.000020000 |
| C | -0.000005000 | -3.937323000 | -0.000005000 |
| C | -2.361274000 | -0.975761000 | 0.000004000 |
| C | 2.361273000 | -0.975761000 | 0.000017000 |

| | | | |
|---|---|---|---|
| C | 3.714111000 | -1.336736000 | -0.000001000 |
| C | 4.687063000 | -0.361944000 | -0.000011000 |
| C | 4.317180000 | 0.982018000 | -0.000004000 |
| C | 2.967624000 | 1.294322000 | 0.000017000 |
| N | -2.023488000 | 0.363059000 | 0.000033000 |
| N | 2.023491000 | 0.363056000 | 0.000029000 |
| C | -2.967620000 | 1.294326000 | 0.000039000 |
| C | -4.317176000 | 0.982026000 | 0.000017000 |
| C | -4.687064000 | -0.361940000 | -0.000012000 |
| C | -3.714116000 | -1.336733000 | -0.000018000 |
| H | -2.152228000 | -3.873339000 | -0.000032000 |
| H | 2.152224000 | -3.873335000 | 0.000021000 |
| H | -0.000005000 | -5.022874000 | -0.000020000 |
| H | 3.979364000 | -2.385761000 | -0.000007000 |
| H | 5.733905000 | -0.641232000 | -0.000026000 |
| H | 5.050645000 | 1.776096000 | -0.000014000 |
| H | 2.615627000 | 2.318647000 | 0.000021000 |
| H | -2.615622000 | 2.318652000 | 0.000057000 |
| H | -5.050638000 | 1.776106000 | 0.000021000 |
| H | -5.733906000 | -0.641226000 | -0.000031000 |
| H | -3.979374000 | -2.385757000 | -0.000041000 |

Sum of electronic and zero-point Energies= -1304.503025
Sum of electronic and thermal Energies= -1304.486879
Sum of electronic and thermal Enthalpies= -1304.485935
Sum of electronic and thermal Free Energies= -1304.548536

**1_OER**

| | | | |
|---|---|---|---|
| Pt | 0.007126000 | -0.670378000 | 0.000015000 |
| Cl | -0.026102000 | -3.130343000 | -0.000155000 |
| C | -1.242505000 | 3.302119000 | -0.000020000 |
| C | -1.220132000 | 1.896244000 | 0.000057000 |
| C | -0.001200000 | 1.237349000 | 0.000040000 |
| C | 1.245372000 | 1.902467000 | -0.000056000 |
| C | 1.203311000 | 3.313332000 | -0.000151000 |
| C | -0.019519000 | 3.977130000 | -0.000124000 |
| C | -2.352983000 | 0.981036000 | 0.000085000 |
| C | 2.352392000 | 1.008772000 | -0.000032000 |
| C | 3.714501000 | 1.351762000 | 0.000054000 |
| C | 4.691471000 | 0.391407000 | 0.000119000 |
| C | 4.308156000 | -0.976979000 | 0.000078000 |
| C | 2.972714000 | -1.283366000 | 0.000001000 |
| N | -2.010808000 | -0.344750000 | 0.000114000 |
| N | 1.998054000 | -0.359024000 | -0.000042000 |
| C | -2.966158000 | -1.274360000 | 0.000072000 |
| C | -4.313424000 | -0.956235000 | 0.000005000 |
| C | -4.679799000 | 0.379272000 | -0.000002000 |
| C | -3.691570000 | 1.350430000 | 0.000047000 |
| H | -2.172300000 | 3.859376000 | 0.000037000 |
| H | 2.118939000 | 3.896766000 | -0.000256000 |
| H | -0.020760000 | 5.062263000 | -0.000192000 |
| H | 3.979415000 | 2.403533000 | 0.000091000 |

| | | | |
|---|---|---|---|
| H | 5.738229000 | 0.671839000 | 0.000219000 |
| H | 5.040584000 | -1.773351000 | 0.000078000 |
| H | 2.625325000 | -2.310528000 | -0.000059000 |
| H | -2.613592000 | -2.298775000 | 0.000103000 |
| H | -5.050200000 | -1.748495000 | -0.000023000 |
| H | -5.725051000 | 0.664920000 | -0.000065000 |
| H | -3.947188000 | 2.401932000 | 0.000028000 |

Sum of electronic and zero-point Energies= -1304.694515
Sum of electronic and thermal Energies= -1304.678452
Sum of electronic and thermal Enthalpies= -1304.677508
Sum of electronic and thermal Free Energies= -1304.739261

**1_ImA**

| | | | |
|---|---|---|---|
| Pt | -0.008118000 | -0.912835000 | 0.000215000 |
| C | 1.251216000 | 3.011425000 | -0.000284000 |
| C | 1.233263000 | 1.614549000 | -0.000180000 |
| C | 0.005447000 | 0.969977000 | -0.000110000 |
| C | -1.242436000 | 1.625895000 | -0.000145000 |
| C | -1.191691000 | 3.030544000 | -0.000242000 |
| C | 0.030082000 | 3.692564000 | -0.000323000 |
| C | 2.365227000 | 0.694899000 | -0.000161000 |
| C | -2.356881000 | 0.734721000 | -0.000125000 |
| C | -3.713316000 | 1.074961000 | -0.000264000 |
| C | -4.689709000 | 0.109882000 | -0.000228000 |
| C | -4.297431000 | -1.256065000 | -0.000065000 |
| C | -2.965382000 | -1.559341000 | 0.000040000 |
| N | 2.004832000 | -0.625731000 | -0.000001000 |
| N | -1.990800000 | -0.626322000 | 0.000038000 |
| C | 2.957089000 | -1.564215000 | 0.000104000 |
| C | 4.303615000 | -1.256509000 | -0.000018000 |
| C | 4.684363000 | 0.076823000 | -0.000235000 |
| C | 3.702021000 | 1.055180000 | -0.000280000 |
| H | 2.182366000 | 3.565875000 | -0.000363000 |
| H | -2.106840000 | 3.613936000 | -0.000297000 |
| H | 0.034811000 | 4.776688000 | -0.000436000 |
| H | -3.981272000 | 2.125734000 | -0.000368000 |
| H | -5.737192000 | 0.384407000 | -0.000301000 |
| H | -5.026704000 | -2.055298000 | -0.000012000 |
| H | -2.625019000 | -2.589342000 | 0.000203000 |
| H | 2.612061000 | -2.590685000 | 0.000312000 |
| H | 5.033161000 | -2.055325000 | 0.000064000 |
| H | 5.731517000 | 0.353023000 | -0.000318000 |
| H | 3.965259000 | 2.104797000 | -0.000407000 |

Sum of electronic and zero-point Energies= -844.435839
Sum of electronic and thermal Energies= -844.421708
Sum of electronic and thermal Enthalpies= -844.420764
Sum of electronic and thermal Free Energies= -844.478161

**1_ImB**

| | | | |
|---|---|---|---|
| Pt | 0.000029000 | -0.911260000 | -0.004057000 |
| C | 1.224388000 | 3.013361000 | -0.009246000 |

| | | | |
|---|---|---|---|
| C | 1.249637000 | 1.621537000 | 0.014324000 |
| C | -0.000023000 | 0.969855000 | 0.043637000 |
| C | -1.249541000 | 1.621483000 | 0.014141000 |
| C | -1.224622000 | 3.013264000 | -0.009516000 |
| C | -0.000106000 | 3.689533000 | -0.015570000 |
| C | 2.371438000 | 0.724040000 | 0.001859000 |
| C | -2.371474000 | 0.723865000 | 0.001738000 |
| C | -3.715040000 | 1.063172000 | 0.007561000 |
| C | -4.709061000 | 0.099857000 | 0.008954000 |
| C | -4.298252000 | -1.256397000 | 0.006804000 |
| C | -2.969522000 | -1.567119000 | -0.003056000 |
| N | 1.974533000 | -0.632678000 | -0.009679000 |
| N | -1.974566000 | -0.632691000 | -0.009601000 |
| C | 2.969509000 | -1.567084000 | -0.003217000 |
| C | 4.298209000 | -1.256434000 | 0.006651000 |
| C | 4.709051000 | 0.099945000 | 0.008949000 |
| C | 3.715133000 | 1.063223000 | 0.007690000 |
| H | 2.147069000 | 3.585566000 | -0.028707000 |
| H | -2.147381000 | 3.585321000 | -0.029047000 |
| H | -0.000120000 | 4.773825000 | -0.036157000 |
| H | -3.972394000 | 2.117415000 | 0.013812000 |
| H | -5.756609000 | 0.371585000 | 0.013224000 |
| H | -5.022492000 | -2.062564000 | 0.010327000 |
| H | -2.636331000 | -2.599189000 | -0.009719000 |
| H | 2.636307000 | -2.599160000 | -0.009920000 |
| H | 5.022438000 | -2.062609000 | 0.010091000 |
| H | 5.756610000 | 0.371643000 | 0.013247000 |
| H | 3.972561000 | 2.117456000 | 0.014042000 |

Sum of electronic and zero-point Energies= -844.505298
Sum of electronic and thermal Energies= -844.490467
Sum of electronic and thermal Enthalpies= -844.489523
Sum of electronic and thermal Free Energies= -844.547806

**1_ImC**

| | | | |
|---|---|---|---|
| Pt | -0.000011000 | -0.621522000 | 0.000247000 |
| C | -1.219628000 | 3.413271000 | 0.004744000 |
| C | -1.217188000 | 2.019200000 | 0.005182000 |
| C | 0.000155000 | 1.347899000 | 0.000671000 |
| C | 1.217618000 | 2.018967000 | -0.005262000 |
| C | 1.220300000 | 3.413036000 | -0.007368000 |
| C | 0.000397000 | 4.090453000 | -0.001876000 |
| C | -2.350801000 | 1.090354000 | 0.007559000 |
| C | 2.351082000 | 1.089928000 | -0.007011000 |
| C | 3.686850000 | 1.463380000 | -0.013874000 |
| C | 4.678748000 | 0.498227000 | -0.013320000 |
| C | 4.315194000 | -0.837862000 | -0.003212000 |
| C | 2.970969000 | -1.168135000 | 0.004384000 |
| N | -2.011890000 | -0.239188000 | 0.000547000 |
| N | 2.011976000 | -0.239570000 | 0.000367000 |
| C | -2.970996000 | -1.167637000 | -0.002695000 |
| C | -4.315172000 | -0.837196000 | 0.005482000 |

| | | | |
|---|---|---|---|
| C | -4.678539000 | 0.498950000 | 0.015200000 |
| C | -3.686516000 | 1.463983000 | 0.014800000 |
| C | -0.000593000 | -2.756475000 | -0.001691000 |
| O | -1.098875000 | -3.383458000 | -0.129862000 |
| O | 1.097284000 | -3.384423000 | 0.125313000 |
| H | -2.141394000 | 3.987092000 | 0.009004000 |
| H | 2.142168000 | 3.986682000 | -0.012986000 |
| H | 0.000496000 | 5.174315000 | -0.002905000 |
| H | 3.939404000 | 2.515904000 | -0.019537000 |
| H | 5.722705000 | 0.789259000 | -0.019364000 |
| H | 5.057290000 | -1.625780000 | -0.000265000 |
| H | 2.578615000 | -2.189268000 | 0.024233000 |
| H | -2.578822000 | -2.188826000 | -0.022828000 |
| H | -5.057370000 | -1.625019000 | 0.003282000 |
| H | -5.722454000 | 0.790116000 | 0.021689000 |
| H | -3.938930000 | 2.516543000 | 0.020124000 |

Sum of electronic and zero-point Energies= -1033.021665
Sum of electronic and thermal Energies= -1033.004495
Sum of electronic and thermal Enthalpies= -1033.003551
Sum of electronic and thermal Free Energies= -1033.067920

**1_ImD**

| | | | |
|---|---|---|---|
| Pt | -0.002515000 | -0.594341000 | 0.015933000 |
| C | -1.224016000 | 3.415176000 | -0.078108000 |
| C | -1.221613000 | 2.022263000 | -0.033615000 |
| C | -0.003117000 | 1.355530000 | 0.006262000 |
| C | 1.213069000 | 2.026280000 | -0.002891000 |
| C | 1.212410000 | 3.419451000 | -0.045166000 |
| C | -0.006613000 | 4.094116000 | -0.081399000 |
| C | -2.355565000 | 1.098457000 | -0.021759000 |
| C | 2.348735000 | 1.105559000 | 0.028269000 |
| C | 3.681447000 | 1.486809000 | 0.054384000 |
| C | 4.678014000 | 0.528926000 | 0.093369000 |
| C | 4.321928000 | -0.809040000 | 0.102131000 |
| C | 2.981082000 | -1.141874000 | 0.069449000 |
| N | -2.013425000 | -0.228065000 | 0.015210000 |
| N | 2.012201000 | -0.223813000 | 0.034105000 |
| C | -2.972797000 | -1.154864000 | 0.053601000 |
| C | -4.316327000 | -0.830443000 | 0.041771000 |
| C | -4.680732000 | 0.504506000 | -0.009050000 |
| C | -3.690906000 | 1.470350000 | -0.037320000 |
| C | -0.070195000 | -2.687647000 | -0.015461000 |
| O | -0.881230000 | -3.420058000 | 0.535155000 |
| H | -2.147290000 | 3.983965000 | -0.111634000 |
| H | 2.134288000 | 3.991362000 | -0.053953000 |
| H | -0.008031000 | 5.176976000 | -0.116050000 |
| H | 3.927425000 | 2.540512000 | 0.047380000 |
| H | 5.719779000 | 0.824848000 | 0.116566000 |
| H | 5.064280000 | -1.595203000 | 0.131468000 |
| H | 2.650642000 | -2.170823000 | 0.054640000 |
| H | -2.624717000 | -2.178853000 | 0.111372000 |

| | | | |
|---|---|---|---|
| H | -5.054730000 | -1.620314000 | 0.073105000 |
| H | -5.724863000 | 0.792798000 | -0.022007000 |
| H | -3.944920000 | 2.521690000 | -0.068956000 |
| O | 0.896117000 | -3.316845000 | -0.761608000 |
| H | 0.724929000 | -4.271350000 | -0.685061000 |

Sum of electronic and zero-point Energies= -1033.514038
Sum of electronic and thermal Energies= -1033.496508
Sum of electronic and thermal Enthalpies= -1033.495564
Sum of electronic and thermal Free Energies= -1033.560842

**1_ImE**

| | | | |
|---|---|---|---|
| Pt | 0.001981000 | -0.581907000 | -0.021147000 |
| C | -1.238066000 | 3.417733000 | 0.051756000 |
| C | -1.230216000 | 2.024812000 | 0.008083000 |
| C | -0.009539000 | 1.365337000 | -0.011679000 |
| C | 1.203659000 | 2.038365000 | 0.017371000 |
| C | 1.195583000 | 3.431211000 | 0.061730000 |
| C | -0.025091000 | 4.101210000 | 0.077057000 |
| C | -2.361818000 | 1.102055000 | -0.015709000 |
| C | 2.345715000 | 1.128359000 | 0.001120000 |
| C | 3.676979000 | 1.513464000 | 0.002802000 |
| C | 4.675691000 | 0.557900000 | -0.024781000 |
| C | 4.324708000 | -0.781572000 | -0.056239000 |
| C | 2.985696000 | -1.115892000 | -0.053793000 |
| N | -2.016259000 | -0.222361000 | -0.035490000 |
| N | 2.014603000 | -0.199528000 | -0.022335000 |
| C | -2.978351000 | -1.148316000 | -0.072231000 |
| C | -4.320674000 | -0.828019000 | -0.085294000 |
| C | -4.685749000 | 0.507756000 | -0.058313000 |
| C | -3.697036000 | 1.473326000 | -0.023990000 |
| C | 0.025949000 | -2.634322000 | 0.071016000 |
| H | -2.164754000 | 3.980512000 | 0.069428000 |
| H | 2.115802000 | 4.004195000 | 0.087217000 |
| H | -0.031242000 | 5.183679000 | 0.112643000 |
| H | 3.920534000 | 2.567280000 | 0.023208000 |
| H | 5.716842000 | 0.855926000 | -0.023954000 |
| H | 5.069003000 | -1.565619000 | -0.081872000 |
| H | 2.672717000 | -2.149929000 | -0.080877000 |
| H | -2.655741000 | -2.179576000 | -0.093159000 |
| H | -5.056469000 | -1.619856000 | -0.116225000 |
| H | -5.729869000 | 0.795095000 | -0.066145000 |
| H | -3.951437000 | 2.524615000 | -0.006438000 |
| O | 0.228864000 | -3.404532000 | -0.951651000 |
| H | 0.215551000 | -4.348614000 | -0.703715000 |
| O | -0.142176000 | -3.332042000 | 1.162858000 |
| H | -0.281998000 | -2.733586000 | 1.911069000 |

Sum of electronic and zero-point Energies= -1033.933006
Sum of electronic and thermal Energies= -1033.915247
Sum of electronic and thermal Enthalpies= -1033.914303
Sum of electronic and thermal Free Energies= -1033.980394

**1_TS**

| | | | |
|---|---|---|---|
| Pt | -0.000088000 | -0.573257000 | -0.024247000 |

| | | | |
|---|---|---|---|
| C | -1.166343000 | 3.448673000 | 0.045651000 |
| C | -1.184111000 | 2.055531000 | 0.018602000 |
| C | 0.024510000 | 1.375425000 | 0.019240000 |
| C | 1.250121000 | 2.024261000 | 0.043826000 |
| C | 1.267725000 | 3.417331000 | 0.073054000 |
| C | 0.059219000 | 4.109143000 | 0.073643000 |
| C | -2.333566000 | 1.154616000 | -0.018680000 |
| C | 2.376017000 | 1.093623000 | 0.025946000 |
| C | 3.713292000 | 1.454713000 | 0.037685000 |
| C | 4.694642000 | 0.480689000 | 0.009307000 |
| C | 4.319433000 | -0.851326000 | -0.034570000 |
| C | 2.974199000 | -1.162045000 | -0.044452000 |
| N | -2.014906000 | -0.176278000 | -0.040716000 |
| N | 2.021577000 | -0.227464000 | -0.011715000 |
| C | -2.994583000 | -1.082642000 | -0.082447000 |
| C | -4.330262000 | -0.734164000 | -0.103591000 |
| C | -4.668352000 | 0.608273000 | -0.079752000 |
| C | -3.660096000 | 1.553605000 | -0.036705000 |
| C | 0.014225000 | -2.596822000 | -0.065491000 |
| H | -2.082168000 | 4.028990000 | 0.044498000 |
| H | 2.198067000 | 3.973790000 | 0.092897000 |
| H | 0.072920000 | 5.191935000 | 0.094473000 |
| H | 3.976043000 | 2.503612000 | 0.068122000 |
| H | 5.741007000 | 0.759649000 | 0.019607000 |
| H | 5.049507000 | -1.648614000 | -0.060669000 |
| H | 2.641552000 | -2.189305000 | -0.085009000 |
| H | -2.692970000 | -2.119833000 | -0.096412000 |
| H | -5.081729000 | -1.510955000 | -0.139171000 |
| H | -5.706224000 | 0.917065000 | -0.095713000 |
| H | -3.892959000 | 2.609867000 | -0.019257000 |
| O | 0.566212000 | -3.500589000 | -0.705763000 |
| H | -0.072648000 | -4.200565000 | 0.200054000 |
| O | -0.772016000 | -3.450953000 | 0.849429000 |
| H | -0.680198000 | -3.256385000 | 1.797920000 |

Sum of electronic and zero-point Energies= -1033.872555
Sum of electronic and thermal Energies= -1033.854850
Sum of electronic and thermal Enthalpies= -1033.853906
Sum of electronic and thermal Free Energies= -1033.919398

**1_ImF**

| | | | |
|---|---|---|---|
| Pt | -0.000002000 | 0.733407000 | -0.000034000 |
| C | -1.217006000 | -3.271562000 | -0.000135000 |
| C | -1.218174000 | -1.878367000 | -0.000100000 |
| C | 0.000005000 | -1.217760000 | -0.000101000 |
| C | 1.218184000 | -1.878364000 | -0.000097000 |
| C | 1.217020000 | -3.271559000 | -0.000131000 |
| C | 0.000008000 | -3.947116000 | -0.000156000 |
| C | -2.357789000 | -0.965148000 | -0.000027000 |
| C | 2.357794000 | -0.965141000 | -0.000025000 |
| C | 3.688916000 | -1.344923000 | 0.000047000 |
| C | 4.682848000 | -0.382887000 | 0.000142000 |

| | | | |
|---|---|---|---|
| C | 4.325742000 | 0.954574000 | 0.000149000 |
| C | 2.985214000 | 1.283531000 | 0.000071000 |
| N | -2.021367000 | 0.359558000 | -0.000008000 |
| N | 2.021366000 | 0.359563000 | -0.000004000 |
| C | -2.985219000 | 1.283522000 | 0.000073000 |
| C | -4.325745000 | 0.954559000 | 0.000164000 |
| C | -4.682845000 | -0.382904000 | 0.000158000 |
| C | -3.688909000 | -1.344935000 | 0.000054000 |
| C | 0.000008000 | 2.693404000 | 0.000074000 |
| O | -0.000027000 | 3.822722000 | 0.000142000 |
| H | -2.140467000 | -3.839042000 | -0.000140000 |
| H | 2.140484000 | -3.839036000 | -0.000131000 |
| H | 0.000009000 | -5.030070000 | -0.000181000 |
| H | 3.937793000 | -2.397499000 | 0.000031000 |
| H | 5.725386000 | -0.675610000 | 0.000207000 |
| H | 5.065942000 | 1.742747000 | 0.000212000 |
| H | 2.669784000 | 2.317030000 | 0.000057000 |
| H | -2.669794000 | 2.317023000 | 0.000051000 |
| H | -5.065949000 | 1.742728000 | 0.000233000 |
| H | -5.725382000 | -0.675631000 | 0.000232000 |
| H | -3.937781000 | -2.397512000 | 0.000038000 |

Sum of electronic and zero-point Energies= -957.588276
Sum of electronic and thermal Energies= -957.572016
Sum of electronic and thermal Enthalpies= -957.571072
Sum of electronic and thermal Free Energies= -957.633427

**1_ImG**

| | | | |
|---|---|---|---|
| Pt | -0.000002000 | 0.733407000 | -0.000034000 |
| C | -1.217006000 | -3.271562000 | -0.000135000 |
| C | -1.218174000 | -1.878367000 | -0.000100000 |
| C | 0.000005000 | -1.217760000 | -0.000101000 |
| C | 1.218184000 | -1.878364000 | -0.000097000 |
| C | 1.217020000 | -3.271559000 | -0.000131000 |
| C | 0.000008000 | -3.947116000 | -0.000156000 |
| C | -2.357789000 | -0.965148000 | -0.000027000 |
| C | 2.357794000 | -0.965141000 | -0.000025000 |
| C | 3.688916000 | -1.344923000 | 0.000047000 |
| C | 4.682848000 | -0.382887000 | 0.000142000 |
| C | 4.325742000 | 0.954574000 | 0.000149000 |
| C | 2.985214000 | 1.283531000 | 0.000071000 |
| N | -2.021367000 | 0.359558000 | -0.000008000 |
| N | 2.021366000 | 0.359563000 | -0.000004000 |
| C | -2.985219000 | 1.283522000 | 0.000073000 |
| C | -4.325745000 | 0.954559000 | 0.000164000 |
| C | -4.682845000 | -0.382904000 | 0.000158000 |
| C | -3.688909000 | -1.344935000 | 0.000054000 |
| C | 0.000008000 | 2.693404000 | 0.000074000 |
| O | -0.000027000 | 3.822722000 | 0.000142000 |
| H | -2.140467000 | -3.839042000 | -0.000140000 |
| H | 2.140484000 | -3.839036000 | -0.000131000 |
| H | 0.000009000 | -5.030070000 | -0.000181000 |
| H | 3.937793000 | -2.397499000 | 0.000031000 |

| | | | |
|---|---|---|---|
| H | 5.725386000 | -0.675610000 | 0.000207000 |
| H | 5.065942000 | 1.742747000 | 0.000212000 |
| H | 2.669784000 | 2.317030000 | 0.000057000 |
| H | -2.669794000 | 2.317023000 | 0.000051000 |
| H | -5.065949000 | 1.742728000 | 0.000233000 |
| H | -5.725382000 | -0.675631000 | 0.000232000 |
| H | -3.937781000 | -2.397512000 | 0.000038000 |

Sum of electronic and zero-point Energies= -957.588276
Sum of electronic and thermal Energies= -957.572016
Sum of electronic and thermal Enthalpies= -957.571072
Sum of electronic and thermal Free Energies= -957.633427

**2_S$_0$**

| | | | |
|---|---|---|---|
| Pt | 2.555756000 | 0.004648000 | 0.004283000 |
| Cl | 4.989695000 | 0.011858000 | 0.008013000 |
| C | 3.166581000 | -2.949107000 | -0.091595000 |
| C | 2.856709000 | -4.294199000 | -0.144483000 |
| C | 1.522834000 | -4.670177000 | -0.165662000 |
| C | 0.544519000 | -3.692896000 | -0.133085000 |
| C | 0.904899000 | -2.355156000 | -0.080958000 |
| N | 2.226791000 | -2.003483000 | -0.060569000 |
| C | -0.014375000 | -1.221301000 | -0.043952000 |
| C | -1.404550000 | -1.220038000 | -0.057165000 |
| C | -2.114430000 | -0.011278000 | -0.018105000 |
| C | -1.413306000 | 1.202324000 | 0.028229000 |
| C | -0.023100000 | 1.213002000 | 0.029411000 |
| C | 0.655074000 | -0.001856000 | -0.003733000 |
| C | 3.145608000 | 2.962182000 | 0.106868000 |
| C | 2.826229000 | 4.305130000 | 0.157645000 |
| C | 1.489717000 | 4.672134000 | 0.166680000 |
| C | 0.518333000 | 3.688318000 | 0.124551000 |
| C | 0.888139000 | 2.353048000 | 0.075165000 |
| N | 2.212534000 | 2.010274000 | 0.066711000 |
| C | -3.587449000 | -0.017549000 | -0.026016000 |
| C | -4.318454000 | -1.020200000 | 0.612713000 |
| C | -5.699856000 | -1.035639000 | 0.612617000 |
| C | -6.437218000 | -0.036006000 | -0.047968000 |
| C | -5.702904000 | 0.985385000 | -0.678012000 |
| C | -4.321502000 | 0.982378000 | -0.665495000 |
| N | -7.806232000 | -0.058487000 | -0.080928000 |
| C | -8.521647000 | 1.090579000 | -0.575619000 |
| C | -8.519083000 | -1.013144000 | 0.729433000 |
| H | 4.187973000 | -2.589432000 | -0.073448000 |
| H | 3.653288000 | -5.025466000 | -0.168715000 |
| H | 1.245943000 | -5.716548000 | -0.207508000 |
| H | -0.504249000 | -3.958719000 | -0.148629000 |
| H | -1.959799000 | -2.149627000 | -0.118814000 |
| H | -1.975195000 | 2.128262000 | 0.083928000 |
| H | 4.169532000 | 2.609445000 | 0.097663000 |
| H | 3.617629000 | 5.041705000 | 0.189618000 |
| H | 1.205425000 | 5.716596000 | 0.206536000 |

| | | | |
|---|---|---|---|
| H | -0.532304000 | 3.947109000 | 0.130608000 |
| H | -3.795471000 | -1.800915000 | 1.154618000 |
| H | -6.208913000 | -1.828175000 | 1.143655000 |
| H | -6.214401000 | 1.786124000 | -1.194281000 |
| H | -3.800889000 | 1.776758000 | -1.189495000 |
| H | -9.589601000 | 0.885021000 | -0.541599000 |
| H | -8.258910000 | 1.303372000 | -1.615516000 |
| H | -8.325980000 | 1.995673000 | 0.015377000 |
| H | -9.586180000 | -0.909605000 | 0.543336000 |
| H | -8.341673000 | -0.871859000 | 1.804468000 |
| H | -8.236227000 | -2.037589000 | 0.472594000 |

Sum of electronic and zero-point Energies= -1669.193769
Sum of electronic and thermal Energies= -1669.168855
Sum of electronic and thermal Enthalpies= -1669.167910
Sum of electronic and thermal Free Energies= -1669.249838

**2_T$_1$**

| | | | |
|---|---|---|---|
| Pt | -2.537275000 | -0.002265000 | 0.001245000 |
| Cl | -4.970295000 | 0.020567000 | 0.005175000 |
| C | -3.168339000 | -2.958241000 | 0.065805000 |
| C | -2.867392000 | -4.299510000 | 0.094693000 |
| C | -1.510235000 | -4.685994000 | 0.097177000 |
| C | -0.538451000 | -3.723090000 | 0.070912000 |
| C | -0.879981000 | -2.357468000 | 0.042879000 |
| N | -2.234579000 | -2.002050000 | 0.040738000 |
| C | 0.015410000 | -1.261100000 | 0.013120000 |
| C | 1.412942000 | -1.233097000 | 0.007412000 |
| C | 2.116614000 | -0.017239000 | -0.007594000 |
| C | 1.409657000 | 1.236547000 | -0.016684000 |
| C | 0.018275000 | 1.223253000 | -0.022211000 |
| C | -0.655030000 | 0.007160000 | -0.005460000 |
| C | -3.146991000 | 2.972172000 | -0.059958000 |
| C | -2.826694000 | 4.319563000 | -0.090392000 |
| C | -1.491740000 | 4.684022000 | -0.097801000 |
| C | -0.519151000 | 3.697279000 | -0.074971000 |
| C | -0.892409000 | 2.362043000 | -0.046544000 |
| N | -2.216248000 | 2.022429000 | -0.038846000 |
| C | 3.557258000 | -0.011761000 | -0.012151000 |
| C | 4.312067000 | -1.161819000 | -0.339527000 |
| C | 5.677607000 | -1.165853000 | -0.340621000 |
| C | 6.410225000 | -0.000210000 | 0.002656000 |
| C | 5.667365000 | 1.159802000 | 0.330383000 |
| C | 4.299571000 | 1.146579000 | 0.311973000 |
| N | 7.755320000 | 0.002008000 | 0.011125000 |
| C | 8.484987000 | 1.206651000 | 0.344790000 |
| C | 8.495876000 | -1.197675000 | -0.317532000 |
| H | -4.191631000 | -2.601739000 | 0.062109000 |
| H | -3.666385000 | -5.027863000 | 0.114887000 |
| H | -1.239044000 | -5.734802000 | 0.119594000 |
| H | 0.509581000 | -3.997682000 | 0.071516000 |
| H | 1.967344000 | -2.161288000 | 0.056002000 |

| | | | |
|---|---|---|---|
| H | 1.956570000 | 2.165801000 | -0.077677000 |
| H | -4.171453000 | 2.620586000 | -0.051882000 |
| H | -3.617817000 | 5.056918000 | -0.107436000 |
| H | -1.206069000 | 5.728780000 | -0.121108000 |
| H | 0.531753000 | 3.954545000 | -0.078808000 |
| H | 3.803839000 | -2.069938000 | -0.634954000 |
| H | 6.197667000 | -2.071099000 | -0.620027000 |
| H | 6.177530000 | 2.071490000 | 0.606996000 |
| H | 3.782490000 | 2.053212000 | 0.595021000 |
| H | 9.550168000 | 1.004261000 | 0.281816000 |
| H | 8.256215000 | 1.538767000 | 1.361139000 |
| H | 8.246281000 | 2.017327000 | -0.348706000 |
| H | 9.558626000 | -0.993114000 | -0.226385000 |
| H | 8.293393000 | -1.520362000 | -1.342524000 |
| H | 8.243304000 | -2.015558000 | 0.362100000 |

Sum of electronic and zero-point Energies= -1669.117446
Sum of electronic and thermal Energies= -1669.092022
Sum of electronic and thermal Enthalpies= -1669.091077
Sum of electronic and thermal Free Energies= -1669.175280

**2_OER**

| | | | |
|---|---|---|---|
| C | -1.413608000 | -1.236917000 | -0.071364000 |
| C | -0.009677000 | -1.214586000 | -0.055275000 |
| C | 0.652329000 | -0.000033000 | -0.012709000 |
| C | -0.018498000 | 1.241249000 | 0.019490000 |
| C | -1.426982000 | 1.197981000 | 0.013099000 |
| C | -2.114702000 | -0.019669000 | -0.035126000 |
| Pt | 2.558115000 | 0.010060000 | 0.008637000 |
| C | 0.903948000 | -2.348174000 | -0.086841000 |
| C | 0.871342000 | 2.350374000 | 0.068014000 |
| C | 0.523845000 | 3.711090000 | 0.108249000 |
| C | 1.481338000 | 4.689545000 | 0.154460000 |
| C | 2.850392000 | 4.311047000 | 0.160344000 |
| C | 3.160951000 | 2.976625000 | 0.118914000 |
| N | 2.230556000 | -2.008355000 | -0.062599000 |
| N | 2.240084000 | 2.000878000 | 0.073564000 |
| C | 3.158964000 | -2.963749000 | -0.088441000 |
| C | 2.839722000 | -4.310147000 | -0.139423000 |
| C | 1.503909000 | -4.674224000 | -0.163578000 |
| C | 0.533445000 | -3.685756000 | -0.136546000 |
| Cl | 5.017669000 | -0.020891000 | 0.018915000 |
| H | -1.961333000 | -2.169224000 | -0.143421000 |
| H | -2.002311000 | 2.116225000 | 0.074280000 |
| H | -0.528591000 | 3.973108000 | 0.101666000 |
| H | 1.197516000 | 5.734978000 | 0.185397000 |
| H | 3.644087000 | 5.045423000 | 0.196345000 |
| H | 4.189089000 | 2.632182000 | 0.120855000 |
| H | 4.183705000 | -2.612754000 | -0.067143000 |
| H | 3.631148000 | -5.047528000 | -0.159089000 |
| H | 1.217331000 | -5.718508000 | -0.203105000 |
| H | -0.518281000 | -3.939979000 | -0.153321000 |

| | | | |
|---|---|---|---|
| C | -3.590614000 | -0.026486000 | -0.044226000 |
| C | -4.329781000 | 0.975397000 | -0.675054000 |
| C | -4.324107000 | -1.032442000 | 0.586670000 |
| C | -5.712712000 | 0.979258000 | -0.683387000 |
| H | -3.808811000 | 1.774288000 | -1.191587000 |
| C | -5.707243000 | -1.047871000 | 0.589392000 |
| H | -3.798166000 | -1.817501000 | 1.119276000 |
| C | -6.446151000 | -0.045313000 | -0.061187000 |
| H | -6.224011000 | 1.785687000 | -1.191371000 |
| H | -6.214109000 | -1.845629000 | 1.115156000 |
| N | -7.822353000 | -0.070349000 | -0.096358000 |
| C | -8.524654000 | -0.991230000 | 0.760743000 |
| H | -9.594161000 | -0.897755000 | 0.580358000 |
| H | -8.242968000 | -2.024227000 | 0.542101000 |
| H | -8.337318000 | -0.808152000 | 1.828790000 |
| C | -8.529751000 | 1.109031000 | -0.526869000 |
| H | -8.262850000 | 1.376379000 | -1.552309000 |
| H | -9.599256000 | 0.906797000 | -0.509045000 |
| H | -8.332278000 | 1.981447000 | 0.112598000 |

Sum of electronic and zero-point Energies= -1669.283354
Sum of electronic and thermal Energies= -1669.258062
Sum of electronic and thermal Enthalpies= -1669.257118
Sum of electronic and thermal Free Energies= -1669.340235

**2_ImA**

| | | | |
|---|---|---|---|
| C | -1.072833000 | -1.239221000 | -0.085220000 |
| C | 0.321233000 | -1.224175000 | -0.066286000 |
| C | 0.971907000 | -0.001882000 | -0.022982000 |
| C | 0.311971000 | 1.241398000 | 0.009232000 |
| C | -1.089390000 | 1.192213000 | 0.000870000 |
| C | -1.779338000 | -0.022782000 | -0.048817000 |
| Pt | 2.852750000 | 0.009156000 | 0.017564000 |
| C | 1.237731000 | -2.359200000 | -0.092778000 |
| C | 1.201558000 | 2.357217000 | 0.064410000 |
| C | 0.859267000 | 3.711947000 | 0.103579000 |
| C | 1.822777000 | 4.689300000 | 0.153287000 |
| C | 3.188819000 | 4.298205000 | 0.162629000 |
| C | 3.494328000 | 2.967077000 | 0.123933000 |
| N | 2.559505000 | -2.002346000 | -0.055325000 |
| N | 2.563009000 | 1.991763000 | 0.076876000 |
| C | 3.495570000 | -2.956969000 | -0.075802000 |
| C | 3.184920000 | -4.301621000 | -0.133953000 |
| C | 1.851111000 | -4.678539000 | -0.171380000 |
| C | 0.874969000 | -3.693936000 | -0.149586000 |
| H | -1.619142000 | -2.172602000 | -0.152367000 |
| H | -1.662582000 | 2.111556000 | 0.060452000 |
| H | -0.191876000 | 3.978280000 | 0.093394000 |
| H | 1.546730000 | 5.735931000 | 0.183810000 |
| H | 3.986816000 | 5.027937000 | 0.200167000 |
| H | 4.524759000 | 2.628229000 | 0.130447000 |
| H | 4.522642000 | -2.615232000 | -0.044061000 |
| H | 3.981966000 | -5.033020000 | -0.148948000 |

| | | | |
|---|---|---|---|
| H | 1.573073000 | -5.724198000 | -0.217132000 |
| H | -0.175240000 | -3.953528000 | -0.177274000 |
| C | -3.253976000 | -0.030170000 | -0.054823000 |
| C | -3.992740000 | 0.971732000 | -0.686019000 |
| C | -3.984386000 | -1.033851000 | 0.583052000 |
| C | -5.374861000 | 0.978694000 | -0.686600000 |
| H | -3.473823000 | 1.767373000 | -1.209687000 |
| C | -5.366787000 | -1.045734000 | 0.594097000 |
| H | -3.458454000 | -1.818554000 | 1.116288000 |
| C | -6.107063000 | -0.041898000 | -0.054676000 |
| H | -5.887678000 | 1.783710000 | -1.195055000 |
| H | -5.872931000 | -1.840146000 | 1.125345000 |
| N | -7.480248000 | -0.060763000 | -0.077490000 |
| C | -8.183600000 | -0.990695000 | 0.769282000 |
| H | -7.908271000 | -2.022100000 | 0.534735000 |
| H | -7.989934000 | -0.822828000 | 1.838233000 |
| H | -9.253228000 | -0.888739000 | 0.595759000 |
| C | -8.190414000 | 1.110074000 | -0.527111000 |
| H | -7.933034000 | 1.355242000 | -1.560719000 |
| H | -9.259759000 | 0.910256000 | -0.494004000 |
| H | -7.985071000 | 1.994553000 | 0.092190000 |

Sum of electronic and zero-point Energies= -1209.024924
Sum of electronic and thermal Energies= -1209.001530
Sum of electronic and thermal Enthalpies= -1209.000586
Sum of electronic and thermal Free Energies= -1209.079581

**2_ImB**

| | | | |
|---|---|---|---|
| Pt | -2.498408000 | 0.008229000 | 0.000377000 |
| C | 1.524848000 | -1.229505000 | -0.148204000 |
| C | 0.133454000 | -1.218709000 | -0.139739000 |
| C | -0.534090000 | -0.001501000 | -0.096083000 |
| C | 0.149744000 | 1.206515000 | -0.045679000 |
| C | 1.541232000 | 1.198666000 | -0.038702000 |
| C | 2.235916000 | -0.020190000 | -0.093262000 |
| C | -0.800940000 | -2.348458000 | -0.157704000 |
| C | -0.769671000 | 2.346428000 | 0.025301000 |
| C | -0.387282000 | 3.679019000 | 0.065150000 |
| C | -1.345578000 | 4.675866000 | 0.125107000 |
| C | -2.684098000 | 4.321091000 | 0.141074000 |
| C | -3.023538000 | 2.979756000 | 0.100234000 |
| N | -2.127332000 | -2.004189000 | -0.083253000 |
| N | -2.101616000 | 2.016048000 | 0.048202000 |
| C | -3.061259000 | -2.957672000 | -0.085056000 |
| C | -2.740681000 | -4.301569000 | -0.170554000 |
| C | -1.408221000 | -4.670167000 | -0.249907000 |
| C | -0.436818000 | -3.684247000 | -0.238806000 |
| C | -4.622784000 | 0.014887000 | 0.171682000 |
| O | -5.230857000 | -1.070094000 | 0.435077000 |
| O | -5.261709000 | 1.102664000 | 0.013414000 |
| H | 2.084260000 | -2.157999000 | -0.207993000 |
| H | 2.112740000 | 2.118460000 | 0.038471000 |

| | | | |
|---|---|---|---|
| H | 0.666634000 | 3.925205000 | 0.045987000 |
| H | -1.047476000 | 5.717368000 | 0.156107000 |
| H | -3.466527000 | 5.067698000 | 0.184256000 |
| H | -4.047429000 | 2.595656000 | 0.098373000 |
| H | -4.076399000 | -2.565632000 | 0.014704000 |
| H | -3.532587000 | -5.039360000 | -0.171183000 |
| H | -1.124703000 | -5.714002000 | -0.317495000 |
| H | 0.613123000 | -3.941362000 | -0.295537000 |
| C | 3.710090000 | -0.031310000 | -0.073935000 |
| C | 4.425418000 | -1.030437000 | 0.587579000 |
| C | 4.462275000 | 0.964141000 | -0.698852000 |
| C | 5.807155000 | -1.046041000 | 0.623749000 |
| H | 3.887564000 | -1.807734000 | 1.119717000 |
| C | 5.844501000 | 0.967257000 | -0.673976000 |
| H | 3.954856000 | 1.757288000 | -1.237554000 |
| C | 6.561825000 | -0.050129000 | -0.020477000 |
| H | 6.300886000 | -1.836292000 | 1.172508000 |
| H | 6.368822000 | 1.767058000 | -1.178871000 |
| N | 7.935683000 | -0.073440000 | -0.017309000 |
| C | 8.619026000 | -0.994762000 | 0.854998000 |
| H | 9.692201000 | -0.899123000 | 0.700495000 |
| H | 8.405652000 | -0.812358000 | 1.917854000 |
| H | 8.344233000 | -2.028044000 | 0.628565000 |
| C | 8.657116000 | 1.093548000 | -0.458741000 |
| H | 9.725158000 | 0.890806000 | -0.405408000 |
| H | 8.419291000 | 1.334715000 | -1.497894000 |
| H | 8.443054000 | 1.981394000 | 0.152865000 |

Sum of electronic and zero-point Energies= -1397.610547
Sum of electronic and thermal Energies= -1397.584142
Sum of electronic and thermal Enthalpies= -1397.583198
Sum of electronic and thermal Free Energies= -1397.668898

**2_ImC**

| | | | |
|---|---|---|---|
| Pt | 2.467511000 | 0.003451000 | 0.002980000 |
| C | -1.544424000 | 1.216788000 | 0.040696000 |
| C | -0.153413000 | 1.216314000 | 0.049298000 |
| C | 0.520857000 | 0.003086000 | 0.072440000 |
| C | -0.154269000 | -1.208956000 | 0.093692000 |
| C | -1.545394000 | -1.209157000 | 0.095577000 |
| C | -2.249214000 | 0.003810000 | 0.062772000 |
| C | 0.766040000 | 2.353030000 | -0.005892000 |
| C | 0.764781000 | -2.346697000 | 0.102789000 |
| C | 0.382807000 | -3.678851000 | 0.141658000 |
| C | 1.340029000 | -4.676853000 | 0.145094000 |
| C | 2.678084000 | -4.322606000 | 0.110969000 |
| C | 3.011803000 | -2.981968000 | 0.073484000 |
| N | 2.093627000 | 2.013918000 | -0.046512000 |
| N | 2.094357000 | -2.012024000 | 0.067318000 |
| C | 3.016362000 | 2.975234000 | -0.116808000 |
| C | 2.687806000 | 4.317728000 | -0.136332000 |
| C | 1.351982000 | 4.678928000 | -0.086246000 |

| | | | |
|---|---|---|---|
| C | 0.389782000 | 3.687024000 | -0.024555000 |
| C | 4.557044000 | 0.071058000 | -0.151206000 |
| O | 5.225811000 | 0.855145000 | -0.810757000 |
| H | -2.105514000 | 2.143500000 | -0.015919000 |
| H | -2.107084000 | -2.136405000 | 0.133251000 |
| H | -0.670971000 | -3.923082000 | 0.169696000 |
| H | 1.043541000 | -5.718286000 | 0.175519000 |
| H | 3.463708000 | -5.066123000 | 0.114288000 |
| H | 4.040965000 | -2.651078000 | 0.059792000 |
| H | 4.040727000 | 2.631219000 | -0.179681000 |
| H | 3.475010000 | 5.057512000 | -0.192151000 |
| H | 1.060258000 | 5.722114000 | -0.098678000 |
| H | -0.662209000 | 3.938094000 | 0.009968000 |
| O | 5.263574000 | -0.861430000 | 0.569605000 |
| H | 6.204496000 | -0.696138000 | 0.386170000 |
| C | -3.722227000 | 0.005256000 | 0.029601000 |
| C | -4.438386000 | -0.983928000 | -0.646078000 |
| C | -4.470854000 | 0.996250000 | 0.665956000 |
| C | -5.818825000 | -0.986376000 | -0.697530000 |
| H | -3.902937000 | -1.768418000 | -1.169962000 |
| C | -5.851954000 | 1.011907000 | 0.627783000 |
| H | -3.962756000 | 1.768316000 | 1.233882000 |
| C | -6.570377000 | 0.025238000 | -0.072166000 |
| H | -6.315559000 | -1.777519000 | -1.242280000 |
| H | -6.376029000 | 1.795459000 | 1.157630000 |
| N | -7.937464000 | 0.050976000 | -0.147372000 |
| C | -8.674756000 | 0.997200000 | 0.650510000 |
| H | -9.735264000 | 0.898941000 | 0.427527000 |
| H | -8.533905000 | 0.841858000 | 1.728970000 |
| H | -8.380995000 | 2.024159000 | 0.416499000 |
| C | -8.639084000 | -1.083658000 | -0.692103000 |
| H | -9.707237000 | -0.876482000 | -0.686209000 |
| H | -8.344434000 | -1.272032000 | -1.728321000 |
| H | -8.463525000 | -2.003370000 | -0.117688000 |

Sum of electronic and zero-point Energies= -1398.103380
Sum of electronic and thermal Energies= -1398.076587
Sum of electronic and thermal Enthalpies= -1398.075643
Sum of electronic and thermal Free Energies= -1398.161875

**2_ImD**

| | | | |
|---|---|---|---|
| Pt | 2.450459000 | 0.002523000 | -0.009534000 |
| C | -1.552920000 | -1.219575000 | -0.126465000 |
| C | -0.162171000 | -1.215522000 | -0.120408000 |
| C | 0.506371000 | -0.001877000 | -0.080703000 |
| C | -0.170345000 | 1.207301000 | -0.036906000 |
| C | -1.561151000 | 1.201919000 | -0.028727000 |
| C | -2.262824000 | -0.011378000 | -0.074846000 |
| C | 0.754912000 | -2.351924000 | -0.150652000 |
| C | 0.739440000 | 2.348562000 | 0.023125000 |
| C | 0.355064000 | 3.679547000 | 0.058846000 |
| C | 1.311142000 | 4.677000000 | 0.107310000 |

| | | | |
|---|---|---|---|
| C | 2.650773000 | 4.325264000 | 0.115894000 |
| C | 2.984433000 | 2.986453000 | 0.080401000 |
| N | 2.080832000 | -2.014060000 | -0.102346000 |
| N | 2.067560000 | 2.016375000 | 0.039040000 |
| C | 3.002926000 | -2.979805000 | -0.134593000 |
| C | 2.677331000 | -4.318819000 | -0.211458000 |
| C | 1.339947000 | -4.676277000 | -0.256204000 |
| C | 0.378323000 | -3.683588000 | -0.225146000 |
| C | 4.500374000 | 0.014214000 | 0.162139000 |
| H | -2.108049000 | -2.149102000 | -0.184225000 |
| H | -2.122137000 | 2.127065000 | 0.041643000 |
| H | -0.698799000 | 3.923577000 | 0.046187000 |
| H | 1.013491000 | 5.717886000 | 0.135898000 |
| H | 3.435352000 | 5.068672000 | 0.149893000 |
| H | 4.018693000 | 2.672861000 | 0.082438000 |
| H | 4.035566000 | -2.662913000 | -0.099002000 |
| H | 3.466379000 | -5.057867000 | -0.235696000 |
| H | 1.048423000 | -5.717527000 | -0.316696000 |
| H | -0.674054000 | -3.931542000 | -0.262089000 |
| O | 5.310821000 | 0.222931000 | -0.827868000 |
| H | 6.244492000 | 0.200687000 | -0.543857000 |
| O | 5.154812000 | -0.169642000 | 1.278292000 |
| H | 4.526204000 | -0.315119000 | 2.000209000 |
| C | -3.734785000 | -0.018071000 | -0.055042000 |
| C | -4.481891000 | 0.989598000 | -0.666990000 |
| C | -4.450571000 | -1.027058000 | 0.591439000 |
| C | -5.862543000 | 0.994639000 | -0.645162000 |
| H | -3.973320000 | 1.787902000 | -1.196819000 |
| C | -5.830721000 | -1.039606000 | 0.627696000 |
| H | -3.915806000 | -1.813932000 | 1.112449000 |
| C | -6.582672000 | -0.030276000 | -0.002668000 |
| H | -6.386114000 | 1.799782000 | -1.141973000 |
| H | -6.328122000 | -1.836000000 | 1.163862000 |
| N | -7.949052000 | -0.045954000 | 0.006321000 |
| C | -8.645735000 | -1.023507000 | 0.803654000 |
| H | -8.435410000 | -0.918675000 | 1.876431000 |
| H | -9.716811000 | -0.905951000 | 0.653618000 |
| H | -8.378355000 | -2.041054000 | 0.504908000 |
| C | -8.678234000 | 1.095936000 | -0.485200000 |
| H | -8.448820000 | 1.291483000 | -1.536638000 |
| H | -9.744742000 | 0.894267000 | -0.412179000 |
| H | -8.461128000 | 2.008982000 | 0.084790000 |

Sum of electronic and zero-point Energies= -1398.523090
Sum of electronic and thermal Energies= -1398.496043
Sum of electronic and thermal Enthalpies= -1398.495099
Sum of electronic and thermal Free Energies= -1398.582381

**2_ImE**

| | | | |
|---|---|---|---|
| Pt | 2.450459000 | 0.002523000 | -0.009534000 |
| C | -1.552920000 | -1.219575000 | -0.126465000 |
| C | -0.162171000 | -1.215522000 | -0.120408000 |

| | | | |
|---|---|---|---|
| C | 0.506371000 | -0.001877000 | -0.080703000 |
| C | -0.170345000 | 1.207301000 | -0.036906000 |
| C | -1.561151000 | 1.201919000 | -0.028727000 |
| C | -2.262824000 | -0.011378000 | -0.074846000 |
| C | 0.754912000 | -2.351924000 | -0.150652000 |
| C | 0.739440000 | 2.348562000 | 0.023125000 |
| C | 0.355064000 | 3.679547000 | 0.058846000 |
| C | 1.311142000 | 4.677000000 | 0.107310000 |
| C | 2.650773000 | 4.325264000 | 0.115894000 |
| C | 2.984433000 | 2.986453000 | 0.080401000 |
| N | 2.080832000 | -2.014060000 | -0.102346000 |
| N | 2.067560000 | 2.016375000 | 0.039040000 |
| C | 3.002926000 | -2.979805000 | -0.134593000 |
| C | 2.677331000 | -4.318819000 | -0.211458000 |
| C | 1.339947000 | -4.676277000 | -0.256204000 |
| C | 0.378323000 | -3.683588000 | -0.225146000 |
| C | 4.500374000 | 0.014214000 | 0.162139000 |
| H | -2.108049000 | -2.149102000 | -0.184225000 |
| H | -2.122137000 | 2.127065000 | 0.041643000 |
| H | -0.698799000 | 3.923577000 | 0.046187000 |
| H | 1.013491000 | 5.717886000 | 0.135898000 |
| H | 3.435352000 | 5.068672000 | 0.149893000 |
| H | 4.018693000 | 2.672861000 | 0.082438000 |
| H | 4.035566000 | -2.662913000 | -0.099002000 |
| H | 3.466379000 | -5.057867000 | -0.235696000 |
| H | 1.048423000 | -5.717527000 | -0.316696000 |
| H | -0.674054000 | -3.931542000 | -0.262089000 |
| O | 5.310821000 | 0.222931000 | -0.827868000 |
| H | 6.244492000 | 0.200687000 | -0.543857000 |
| O | 5.154812000 | -0.169642000 | 1.278292000 |
| H | 4.526204000 | -0.315119000 | 2.000209000 |
| C | -3.734785000 | -0.018071000 | -0.055042000 |
| C | -4.481891000 | 0.989598000 | -0.666990000 |
| C | -4.450571000 | -1.027058000 | 0.591439000 |
| C | -5.862543000 | 0.994639000 | -0.645162000 |
| H | -3.973320000 | 1.787902000 | -1.196819000 |
| C | -5.830721000 | -1.039606000 | 0.627696000 |
| H | -3.915806000 | -1.813932000 | 1.112449000 |
| C | -6.582672000 | -0.030276000 | -0.002668000 |
| H | -6.386114000 | 1.799782000 | -1.141973000 |
| H | -6.328122000 | -1.836000000 | 1.163862000 |
| N | -7.949052000 | -0.045954000 | 0.006321000 |
| C | -8.645735000 | -1.023507000 | 0.803654000 |
| H | -8.435410000 | -0.918675000 | 1.876431000 |
| H | -9.716811000 | -0.905951000 | 0.653618000 |
| H | -8.378355000 | -2.041054000 | 0.504908000 |
| C | -8.678234000 | 1.095936000 | -0.485200000 |
| H | -8.448820000 | 1.291483000 | -1.536638000 |
| H | -9.744742000 | 0.894267000 | -0.412179000 |
| H | -8.461128000 | 2.008982000 | 0.084790000 |

Sum of electronic and zero-point Energies= -1398.523090

Sum of electronic and thermal Energies= -1398.496043
Sum of electronic and thermal Enthalpies= -1398.495099
Sum of electronic and thermal Free Energies= -1398.582381

**2_TS**

| | | | |
|---|---|---|---|
| Pt | 2.443224000 | 0.015641000 | -0.022944000 |
| C | -1.555836000 | -1.222922000 | 0.089016000 |
| C | -0.165469000 | -1.214047000 | 0.068349000 |
| C | 0.496972000 | 0.002395000 | 0.036275000 |
| C | -0.182138000 | 1.210233000 | 0.018225000 |
| C | -1.572472000 | 1.200657000 | 0.027854000 |
| C | -2.269685000 | -0.015874000 | 0.063769000 |
| C | 0.755116000 | -2.348878000 | 0.072442000 |
| C | 0.722681000 | 2.356159000 | -0.040813000 |
| C | 0.331818000 | 3.684567000 | -0.076340000 |
| C | 1.283465000 | 4.685955000 | -0.141907000 |
| C | 2.623550000 | 4.339399000 | -0.173578000 |
| C | 2.964437000 | 3.001880000 | -0.135333000 |
| N | 2.080139000 | -2.008059000 | 0.026464000 |
| N | 2.051762000 | 2.030289000 | -0.067707000 |
| C | 3.003474000 | -2.972413000 | 0.017839000 |
| C | 2.678937000 | -4.313879000 | 0.056652000 |
| C | 1.343054000 | -4.674164000 | 0.107062000 |
| C | 0.380075000 | -3.681798000 | 0.114019000 |
| C | 4.464254000 | 0.073654000 | -0.106498000 |
| H | -2.107520000 | -2.154797000 | 0.139760000 |
| H | -2.137007000 | 2.124862000 | -0.021085000 |
| H | -0.722861000 | 3.924205000 | -0.053554000 |
| H | 0.981012000 | 5.725469000 | -0.169664000 |
| H | 3.404298000 | 5.085683000 | -0.227238000 |
| H | 3.998884000 | 2.691122000 | -0.165577000 |
| H | 4.034810000 | -2.652980000 | -0.018825000 |
| H | 3.468747000 | -5.052445000 | 0.046505000 |
| H | 1.052879000 | -5.717032000 | 0.139596000 |
| H | -0.671740000 | -3.931869000 | 0.151146000 |
| O | 5.340282000 | 0.641028000 | -0.772232000 |
| H | 6.075658000 | 0.024464000 | 0.118823000 |
| O | 5.360466000 | -0.687681000 | 0.792928000 |
| H | 5.189194000 | -0.586773000 | 1.744932000 |
| C | -3.741164000 | -0.025989000 | 0.061237000 |
| C | -4.462473000 | -1.029507000 | -0.587975000 |
| C | -4.482850000 | 0.974020000 | 0.692466000 |
| C | -5.842680000 | -1.043339000 | -0.609742000 |
| H | -3.932499000 | -1.809855000 | -1.123438000 |
| C | -5.863376000 | 0.977120000 | 0.686123000 |
| H | -3.969741000 | 1.767084000 | 1.225715000 |
| C | -6.589055000 | -0.041414000 | 0.039286000 |
| H | -6.345039000 | -1.834316000 | -1.149264000 |
| H | -6.382750000 | 1.775594000 | 1.197862000 |
| N | -7.954683000 | -0.057577000 | 0.043077000 |
| C | -8.659694000 | -1.031565000 | -0.751349000 |

| | | | |
|---|---|---|---|
| H | -9.729119000 | -0.914311000 | -0.590135000 |
| H | -8.460083000 | -0.921409000 | -1.825500000 |
| H | -8.389434000 | -2.050638000 | -0.460245000 |
| C | -8.680499000 | 1.074259000 | 0.562117000 |
| H | -9.747400000 | 0.873282000 | 0.494042000 |
| H | -8.442284000 | 1.250778000 | 1.615065000 |
| H | -8.468589000 | 1.997501000 | 0.007075000 |

Sum of electronic and zero-point Energies= -1398.462909
Sum of electronic and thermal Energies= -1398.435864
Sum of electronic and thermal Enthalpies= -1398.434919
Sum of electronic and thermal Free Energies= -1398.522087

**2_ImF**

| | | | |
|---|---|---|---|
| Pt | 2.633646000 | 0.000976000 | 0.001030000 |
| C | -1.370752000 | -1.213919000 | -0.027677000 |
| C | 0.018952000 | -1.214259000 | -0.017706000 |
| C | 0.685482000 | -0.001095000 | 0.019539000 |
| C | 0.016986000 | 1.211108000 | 0.053023000 |
| C | -1.372715000 | 1.208753000 | 0.056956000 |
| C | -2.078093000 | -0.003059000 | 0.012091000 |
| C | 0.930009000 | -2.355337000 | -0.062346000 |
| C | 0.926151000 | 2.353956000 | 0.089819000 |
| C | 0.542891000 | 3.682925000 | 0.145033000 |
| C | 1.502099000 | 4.679047000 | 0.178995000 |
| C | 2.840571000 | 4.326468000 | 0.155372000 |
| C | 3.173204000 | 2.987950000 | 0.098858000 |
| N | 2.255566000 | -2.020768000 | -0.055637000 |
| N | 2.252149000 | 2.022058000 | 0.066680000 |
| C | 3.178052000 | -2.985117000 | -0.093243000 |
| C | 2.847340000 | -4.324471000 | -0.140964000 |
| C | 1.509388000 | -4.679641000 | -0.149681000 |
| C | 0.548718000 | -3.685187000 | -0.109051000 |
| C | 4.591106000 | 0.004313000 | -0.042920000 |
| O | 5.720507000 | 0.006816000 | -0.078794000 |
| H | -1.925921000 | -2.142490000 | -0.091201000 |
| H | -1.929368000 | 2.136831000 | 0.113933000 |
| H | -0.510520000 | 3.927906000 | 0.162927000 |
| H | 1.206417000 | 5.719801000 | 0.223988000 |
| H | 3.626632000 | 5.068503000 | 0.180369000 |
| H | 4.207443000 | 2.675357000 | 0.079394000 |
| H | 4.211935000 | -2.670907000 | -0.085214000 |
| H | 3.634507000 | -5.065154000 | -0.170819000 |
| H | 1.215237000 | -5.721112000 | -0.187590000 |
| H | -0.504349000 | -3.932199000 | -0.113840000 |
| C | -3.548054000 | -0.004622000 | 0.001473000 |
| C | -4.283061000 | -1.023399000 | 0.611307000 |
| C | -4.275603000 | 1.013423000 | -0.618496000 |
| C | -5.662788000 | -1.034789000 | 0.606238000 |
| H | -3.765194000 | -1.819853000 | 1.134675000 |
| C | -5.655143000 | 1.018954000 | -0.640133000 |
| H | -3.751412000 | 1.816422000 | -1.125245000 |

| | | | |
|---|---|---|---|
| C | -6.394757000 | -0.013694000 | -0.031009000 |
| H | -6.177824000 | -1.839282000 | 1.112836000 |
| H | -6.163803000 | 1.828220000 | -1.145630000 |
| N | -7.756909000 | -0.024775000 | -0.058550000 |
| C | -8.482876000 | -1.056070000 | 0.638278000 |
| H | -8.301651000 | -1.033245000 | 1.720213000 |
| H | -9.548310000 | -0.916629000 | 0.470263000 |
| H | -8.213532000 | -2.051672000 | 0.271661000 |
| C | -8.474285000 | 1.090131000 | -0.623298000 |
| H | -8.226131000 | 1.234074000 | -1.679899000 |
| H | -9.542377000 | 0.896829000 | -0.555838000 |
| H | -8.262807000 | 2.028098000 | -0.095183000 |

Sum of electronic and zero-point Energies= -1322.179305
Sum of electronic and thermal Energies= -1322.153563
Sum of electronic and thermal Enthalpies= -1322.152618
Sum of electronic and thermal Free Energies= -1322.237784

**2_ImG**

| | | | |
|---|---|---|---|
| Pt | 2.633150000 | 0.003301000 | 0.002004000 |
| C | -1.365246000 | -1.220977000 | -0.021754000 |
| C | 0.024169000 | -1.227285000 | -0.011200000 |
| C | 0.697640000 | -0.002747000 | 0.029909000 |
| C | 0.017780000 | 1.218468000 | 0.063241000 |
| C | -1.371557000 | 1.205472000 | 0.061499000 |
| C | -2.073268000 | -0.009427000 | 0.015096000 |
| C | 0.926205000 | -2.355415000 | -0.058742000 |
| C | 0.913842000 | 2.351883000 | 0.098502000 |
| C | 0.539976000 | 3.688156000 | 0.152181000 |
| C | 1.491707000 | 4.686185000 | 0.183392000 |
| C | 2.841237000 | 4.319824000 | 0.158528000 |
| C | 3.172996000 | 2.990292000 | 0.104573000 |
| N | 2.264525000 | -1.999513000 | -0.056844000 |
| N | 2.253907000 | 2.003889000 | 0.073742000 |
| C | 3.188749000 | -2.980876000 | -0.096198000 |
| C | 2.863917000 | -4.312486000 | -0.139536000 |
| C | 1.516274000 | -4.686710000 | -0.143133000 |
| C | 0.559383000 | -3.694047000 | -0.101512000 |
| C | 4.569970000 | 0.011901000 | -0.055690000 |
| O | 5.712503000 | 0.018403000 | -0.104164000 |
| H | -1.922514000 | -2.149318000 | -0.085834000 |
| H | -1.933584000 | 2.131657000 | 0.113389000 |
| H | -0.515385000 | 3.929778000 | 0.170525000 |
| H | 1.199202000 | 5.727557000 | 0.226848000 |
| H | 3.630250000 | 5.059719000 | 0.181242000 |
| H | 4.208058000 | 2.677923000 | 0.085038000 |
| H | 4.222214000 | -2.662691000 | -0.092060000 |
| H | 3.656786000 | -5.047966000 | -0.170072000 |
| H | 1.229203000 | -5.729941000 | -0.177202000 |
| H | -0.494701000 | -3.941821000 | -0.101287000 |
| C | -3.545984000 | -0.014060000 | -0.004847000 |
| C | -4.285837000 | -1.021519000 | 0.616668000 |

| | | | |
|---|---|---|---|
| C | -4.273934000 | 0.990745000 | -0.644547000 |
| C | -5.667414000 | -1.037542000 | 0.598661000 |
| H | -3.769697000 | -1.806824000 | 1.158490000 |
| C | -5.655258000 | 0.993168000 | -0.674722000 |
| H | -3.747611000 | 1.789823000 | -1.155455000 |
| C | -6.397082000 | -0.034133000 | -0.064145000 |
| H | -6.182568000 | -1.834800000 | 1.116720000 |
| H | -6.160232000 | 1.798316000 | -1.190655000 |
| N | -7.766725000 | -0.059289000 | -0.118562000 |
| C | -8.488579000 | -1.013471000 | 0.684194000 |
| H | -8.326535000 | -0.869972000 | 1.761552000 |
| H | -9.553285000 | -0.912789000 | 0.482893000 |
| H | -8.200134000 | -2.037748000 | 0.433335000 |
| C | -8.475647000 | 1.096502000 | -0.606633000 |
| H | -8.194712000 | 1.326630000 | -1.637925000 |
| H | -9.543632000 | 0.888042000 | -0.595784000 |
| H | -8.293094000 | 1.992736000 | 0.002202000 |

Sum of electronic and zero-point Energies= -1322.300221
Sum of electronic and thermal Energies= -1322.274498
Sum of electronic and thermal Enthalpies= -1322.273554
Sum of electronic and thermal Free Energies= -1322.358274

**3_S$_0$**

| | | | |
|---|---|---|---|
| Pt | 1.771945000 | 0.051636000 | -0.000450000 |
| Cl | 4.197847000 | 0.169380000 | -0.016104000 |
| C | -2.118259000 | -1.373410000 | 0.023732000 |
| C | -0.731999000 | -1.302709000 | 0.012082000 |
| C | -0.124578000 | -0.049257000 | 0.022593000 |
| C | -0.861538000 | 1.131570000 | 0.049361000 |
| C | -2.248041000 | 1.052076000 | 0.076091000 |
| C | -2.882669000 | -0.197986000 | 0.062886000 |
| C | 0.242364000 | -2.390041000 | -0.025138000 |
| C | -0.007509000 | 2.316247000 | 0.057509000 |
| C | -0.442475000 | 3.632002000 | 0.084523000 |
| C | 0.480026000 | 4.662853000 | 0.089195000 |
| C | 1.832373000 | 4.361239000 | 0.066218000 |
| C | 2.217067000 | 3.034710000 | 0.039306000 |
| N | 1.544071000 | -1.971479000 | -0.033598000 |
| N | 1.331579000 | 2.037889000 | 0.034762000 |
| C | 2.530045000 | -2.868465000 | -0.067778000 |
| C | 2.287469000 | -4.228120000 | -0.095650000 |
| C | 0.974530000 | -4.671417000 | -0.087289000 |
| C | -0.051545000 | -3.744317000 | -0.051383000 |
| C | -4.340532000 | -0.294046000 | 0.093827000 |
| C | -5.117549000 | -1.316150000 | 0.577475000 |
| C | -6.505354000 | -1.076955000 | 0.442675000 |
| C | -6.775481000 | 0.124717000 | -0.143130000 |
| S | -5.340323000 | 0.960252000 | -0.536044000 |
| H | -2.629356000 | -2.328613000 | -0.014342000 |
| H | -2.856494000 | 1.949073000 | 0.125509000 |
| H | -1.504322000 | 3.839999000 | 0.101645000 |

| | | | |
|---|---|---|---|
| H | 0.145201000 | 5.692745000 | 0.110633000 |
| H | 2.587066000 | 5.136048000 | 0.069005000 |
| H | 3.256655000 | 2.732120000 | 0.021189000 |
| H | 3.531739000 | -2.456873000 | -0.072303000 |
| H | 3.119640000 | -4.918496000 | -0.123360000 |
| H | 0.750551000 | -5.730956000 | -0.108685000 |
| H | -1.085678000 | -4.063092000 | -0.044162000 |
| H | -4.703307000 | -2.202308000 | 1.040454000 |
| H | -7.272780000 | -1.763863000 | 0.774384000 |
| H | -7.736526000 | 0.563000000 | -0.365183000 |

Sum of electronic and zero-point Energies= -1856.139329
Sum of electronic and thermal Energies= -1856.119150
Sum of electronic and thermal Enthalpies= -1856.118206
Sum of electronic and thermal Free Energies= -1856.190014

**3_T1**

| | | | |
|---|---|---|---|
| Pt | 1.750354000 | 0.060195000 | 0.000010000 |
| Cl | 4.171399000 | 0.215428000 | -0.000204000 |
| C | -2.098575000 | -1.434322000 | 0.000025000 |
| C | -0.712527000 | -1.366695000 | 0.000017000 |
| C | -0.121674000 | -0.044495000 | 0.000015000 |
| C | -0.875853000 | 1.128481000 | 0.000020000 |
| C | -2.253450000 | 1.051376000 | 0.000027000 |
| C | -2.874194000 | -0.265345000 | 0.000031000 |
| C | 0.254155000 | -2.404828000 | 0.000008000 |
| C | -0.034076000 | 2.324651000 | 0.000021000 |
| C | -0.488445000 | 3.631653000 | 0.000026000 |
| C | 0.423993000 | 4.675994000 | 0.000024000 |
| C | 1.777142000 | 4.389809000 | 0.000018000 |
| C | 2.179232000 | 3.064488000 | 0.000013000 |
| N | 1.574678000 | -1.956956000 | 0.000003000 |
| N | 1.305662000 | 2.061051000 | 0.000015000 |
| C | 2.571910000 | -2.846794000 | -0.000008000 |
| C | 2.357362000 | -4.205752000 | -0.000015000 |
| C | 1.034485000 | -4.683260000 | -0.000011000 |
| C | -0.000776000 | -3.785578000 | 0.000000000 |
| C | -4.295046000 | -0.361186000 | 0.000041000 |
| C | -5.105536000 | -1.497109000 | 0.000117000 |
| C | -6.469352000 | -1.204187000 | 0.000088000 |
| C | -6.720225000 | 0.150224000 | -0.000020000 |
| S | -5.288964000 | 1.061312000 | -0.000027000 |
| H | -2.599834000 | -2.394028000 | 0.000018000 |
| H | -2.876636000 | 1.935752000 | 0.000039000 |
| H | -1.553074000 | 3.824898000 | 0.000030000 |
| H | 0.077055000 | 5.702029000 | 0.000028000 |
| H | 2.523204000 | 5.172965000 | 0.000017000 |
| H | 3.222882000 | 2.775619000 | 0.000009000 |
| H | 3.568764000 | -2.423121000 | -0.000010000 |
| H | 3.203427000 | -4.879207000 | -0.000024000 |
| H | 0.835042000 | -5.747839000 | -0.000018000 |
| H | -1.028395000 | -4.127476000 | 0.000002000 |

| | | | |
|---|---|---|---|
| H | -4.713983000 | -2.504553000 | 0.000195000 |
| H | -7.251065000 | -1.951519000 | 0.000132000 |
| H | -7.678998000 | 0.646571000 | -0.000072000 |

Sum of electronic and zero-point Energies= -1856.059350
Sum of electronic and thermal Energies= -1856.038631
Sum of electronic and thermal Enthalpies= -1856.037687
Sum of electronic and thermal Free Energies= -1856.111682

**3_OER**

| | | | |
|---|---|---|---|
| Pt | 1.773218000 | 0.051827000 | -0.002161000 |
| Cl | 4.222815000 | 0.221379000 | -0.017390000 |
| C | -2.130524000 | -1.388306000 | 0.028235000 |
| C | -0.718365000 | -1.336593000 | 0.014835000 |
| C | -0.128993000 | -0.054387000 | 0.025894000 |
| C | -0.861649000 | 1.119096000 | 0.058476000 |
| C | -2.270031000 | 1.052342000 | 0.090896000 |
| C | -2.884192000 | -0.210703000 | 0.074109000 |
| C | 0.238186000 | -2.385370000 | -0.023193000 |
| C | -0.021822000 | 2.301499000 | 0.064176000 |
| C | -0.472999000 | 3.618312000 | 0.090961000 |
| C | 0.435141000 | 4.662114000 | 0.092217000 |
| C | 1.792402000 | 4.383132000 | 0.066213000 |
| C | 2.192101000 | 3.056886000 | 0.039132000 |
| N | 1.578158000 | -1.961484000 | -0.039059000 |
| N | 1.325317000 | 2.048196000 | 0.037965000 |
| C | 2.556918000 | -2.871542000 | -0.075983000 |
| C | 2.330369000 | -4.227012000 | -0.099487000 |
| C | 0.990757000 | -4.682095000 | -0.082570000 |
| C | -0.027460000 | -3.768255000 | -0.044612000 |
| C | -4.343177000 | -0.309822000 | 0.102745000 |
| C | -5.133303000 | -1.316336000 | 0.598673000 |
| C | -6.519971000 | -1.072588000 | 0.447672000 |
| C | -6.781069000 | 0.119456000 | -0.161516000 |
| S | -5.334153000 | 0.937895000 | -0.559143000 |
| H | -2.651581000 | -2.338256000 | -0.022944000 |
| H | -2.878462000 | 1.946164000 | 0.166529000 |
| H | -1.538065000 | 3.809421000 | 0.108443000 |
| H | 0.084512000 | 5.687487000 | 0.113056000 |
| H | 2.536533000 | 5.168211000 | 0.066256000 |
| H | 3.236106000 | 2.768172000 | 0.018398000 |
| H | 3.560587000 | -2.461506000 | -0.086316000 |
| H | 3.166395000 | -4.912530000 | -0.129774000 |
| H | 0.771119000 | -5.743595000 | -0.099017000 |
| H | -1.060668000 | -4.095897000 | -0.029834000 |
| H | -4.725029000 | -2.195063000 | 1.080558000 |
| H | -7.293287000 | -1.749828000 | 0.787050000 |
| H | -7.738787000 | 0.558971000 | -0.395495000 |

Sum of electronic and zero-point Energies= -1856.231641
Sum of electronic and thermal Energies= -1856.210950
Sum of electronic and thermal Enthalpies= -1856.210006
Sum of electronic and thermal Free Energies= -1856.283253

**3_ImA**

| | | | |
|---|---|---|---|
| Pt | 2.043041000 | 0.107711000 | -0.010213000 |
| C | -1.787959000 | -1.401671000 | 0.036825000 |
| C | -0.385368000 | -1.333666000 | 0.024075000 |
| C | 0.167952000 | -0.035387000 | 0.035963000 |
| C | -0.579319000 | 1.127932000 | 0.064949000 |
| C | -1.973052000 | 1.031796000 | 0.094322000 |
| C | -2.568800000 | -0.243796000 | 0.079042000 |
| C | 0.589704000 | -2.369312000 | -0.018999000 |
| C | 0.239794000 | 2.335383000 | 0.067347000 |
| C | -0.235139000 | 3.636023000 | 0.097988000 |
| C | 0.656196000 | 4.698172000 | 0.096372000 |
| C | 2.015711000 | 4.436328000 | 0.063129000 |
| C | 2.438093000 | 3.119721000 | 0.031799000 |
| N | 1.916183000 | -1.899786000 | -0.043129000 |
| N | 1.585390000 | 2.093458000 | 0.033520000 |
| C | 2.923889000 | -2.791291000 | -0.079188000 |
| C | 2.730867000 | -4.145083000 | -0.096414000 |
| C | 1.400797000 | -4.643142000 | -0.074987000 |
| C | 0.359936000 | -3.752487000 | -0.036389000 |
| C | -4.024869000 | -0.374282000 | 0.102445000 |
| C | -4.788748000 | -1.410914000 | 0.577278000 |
| C | -6.179999000 | -1.200802000 | 0.424411000 |
| C | -6.469182000 | -0.004772000 | -0.164057000 |
| S | -5.044645000 | 0.860260000 | -0.538377000 |
| H | -2.286852000 | -2.363464000 | -0.009679000 |
| H | -2.595452000 | 1.916749000 | 0.155085000 |
| H | -1.303157000 | 3.808344000 | 0.122130000 |
| H | 0.290731000 | 5.717448000 | 0.120569000 |
| H | 2.747937000 | 5.232560000 | 0.060406000 |
| H | 3.490050000 | 2.864495000 | 0.003876000 |
| H | 3.922354000 | -2.367580000 | -0.094779000 |
| H | 3.585148000 | -4.807950000 | -0.125974000 |
| H | 1.211976000 | -5.709576000 | -0.087762000 |
| H | -0.664892000 | -4.105971000 | -0.017109000 |
| H | -4.361296000 | -2.288511000 | 1.044424000 |
| H | -6.936694000 | -1.904288000 | 0.746687000 |
| H | -7.436762000 | 0.413901000 | -0.395411000 |

Sum of electronic and zero-point Energies= -1395.972832
Sum of electronic and thermal Energies= -1395.954090
Sum of electronic and thermal Enthalpies= -1395.953146
Sum of electronic and thermal Free Energies= -1396.022122

**3_ImB**

| | | | |
|---|---|---|---|
| Pt | 2.039919000 | 0.110743000 | -0.006814000 |
| C | -1.769113000 | -1.424375000 | 0.041950000 |
| C | -0.387543000 | -1.332957000 | 0.058078000 |
| C | 0.171248000 | -0.041228000 | 0.117032000 |
| C | -0.583352000 | 1.147559000 | 0.082988000 |
| C | -1.962883000 | 1.016855000 | 0.071255000 |
| C | -2.566976000 | -0.258741000 | 0.064742000 |

| | | | |
|---|---|---|---|
| C | 0.594269000 | -2.384469000 | -0.004728000 |
| C | 0.221088000 | 2.340849000 | 0.052658000 |
| C | -0.228406000 | 3.651310000 | 0.073724000 |
| C | 0.652005000 | 4.718979000 | 0.057166000 |
| C | 2.034370000 | 4.421976000 | 0.020824000 |
| C | 2.452131000 | 3.121999000 | -0.005303000 |
| N | 1.910683000 | -1.887032000 | -0.045713000 |
| N | 1.601342000 | 2.057571000 | 0.004752000 |
| C | 2.919376000 | -2.802556000 | -0.079969000 |
| C | 2.712420000 | -4.152526000 | -0.083328000 |
| C | 1.394644000 | -4.664205000 | -0.053073000 |
| C | 0.356641000 | -3.749356000 | -0.011359000 |
| C | -4.015602000 | -0.390128000 | 0.061738000 |
| C | -4.788340000 | -1.498507000 | 0.331501000 |
| C | -6.178789000 | -1.261661000 | 0.225996000 |
| C | -6.473985000 | 0.023416000 | -0.122304000 |
| S | -5.045635000 | 0.945565000 | -0.330548000 |
| H | -2.253040000 | -2.393156000 | -0.017141000 |
| H | -2.595035000 | 1.900042000 | 0.077012000 |
| H | -1.299342000 | 3.822126000 | 0.106870000 |
| H | 0.296167000 | 5.740897000 | 0.073258000 |
| H | 2.777873000 | 5.210014000 | 0.007550000 |
| H | 3.506813000 | 2.873380000 | -0.042807000 |
| H | 3.921233000 | -2.389197000 | -0.110715000 |
| H | 3.570898000 | -4.812853000 | -0.114130000 |
| H | 1.204593000 | -5.729572000 | -0.060211000 |
| H | -0.673704000 | -4.087413000 | 0.020824000 |
| H | -4.367778000 | -2.452653000 | 0.620037000 |
| H | -6.933667000 | -2.015440000 | 0.411230000 |
| H | -7.441905000 | 0.479635000 | -0.262295000 |

Sum of electronic and zero-point Energies= -1396.045100
Sum of electronic and thermal Energies= -1396.025630
Sum of electronic and thermal Enthalpies= -1396.024685
Sum of electronic and thermal Free Energies= -1396.094852

**3_ImD**

| | | | |
|---|---|---|---|
| Pt | -1.719763000 | -0.040176000 | 0.012387000 |
| C | 2.244780000 | 1.374329000 | 0.067796000 |
| C | 0.857295000 | 1.302448000 | 0.080970000 |
| C | 0.241730000 | 0.056193000 | 0.101626000 |
| C | 0.974931000 | -1.124049000 | 0.105040000 |
| C | 2.363487000 | -1.059015000 | 0.106128000 |
| C | 3.002023000 | 0.191231000 | 0.087686000 |
| C | -0.124097000 | 2.391537000 | 0.037507000 |
| C | 0.104498000 | -2.304198000 | 0.099836000 |
| C | 0.543800000 | -3.618916000 | 0.130856000 |
| C | -0.371656000 | -4.657184000 | 0.128297000 |
| C | -1.723602000 | -4.360080000 | 0.095810000 |
| C | -2.119833000 | -3.034047000 | 0.063719000 |
| N | -1.434255000 | 1.987411000 | -0.009198000 |
| N | -1.239554000 | -2.030904000 | 0.062285000 |

| | | | |
|---|---|---|---|
| C | -2.407667000 | 2.899450000 | -0.055575000 |
| C | -2.143212000 | 4.258266000 | -0.050182000 |
| C | -0.827559000 | 4.686684000 | -0.000634000 |
| C | 0.184526000 | 3.743177000 | 0.040066000 |
| C | 4.461280000 | 0.278800000 | 0.077176000 |
| C | 5.262583000 | 1.297652000 | 0.528007000 |
| C | 6.644130000 | 1.049378000 | 0.346291000 |
| C | 6.887861000 | -0.156471000 | -0.242252000 |
| S | 5.433390000 | -0.984876000 | -0.580655000 |
| C | -3.842332000 | -0.143970000 | -0.152767000 |
| O | -4.499431000 | 0.907291000 | -0.432391000 |
| O | -4.430068000 | -1.256296000 | 0.027214000 |
| H | 2.766192000 | 2.324426000 | 0.015553000 |
| H | 2.974307000 | -1.956726000 | 0.131119000 |
| H | 1.607154000 | -3.819585000 | 0.159525000 |
| H | -0.029894000 | -5.685273000 | 0.153817000 |
| H | -2.473929000 | -5.140134000 | 0.095287000 |
| H | -3.159177000 | -2.693763000 | 0.043921000 |
| H | -3.405976000 | 2.459105000 | -0.124463000 |
| H | -2.965544000 | 4.961002000 | -0.087366000 |
| H | -0.588059000 | 5.743582000 | 0.004540000 |
| H | 1.222751000 | 4.047163000 | 0.077041000 |
| H | 4.869780000 | 2.189720000 | 0.998184000 |
| H | 7.426310000 | 1.733827000 | 0.647835000 |
| H | 7.838202000 | -0.602404000 | -0.493308000 |

Sum of electronic and zero-point Energies= -1584.558603
Sum of electronic and thermal Energies= -1584.536934
Sum of electronic and thermal Enthalpies= -1584.535990
Sum of electronic and thermal Free Energies= -1584.611493

**3_ImD**

| | | | |
|---|---|---|---|
| Pt | -1.692301000 | -0.037339000 | -0.025982000 |
| C | 2.248484000 | 1.374977000 | -0.034583000 |
| C | 0.861339000 | 1.304996000 | -0.060835000 |
| C | 0.250078000 | 0.058406000 | -0.110613000 |
| C | 0.980936000 | -1.121367000 | -0.127380000 |
| C | 2.369446000 | -1.054181000 | -0.118255000 |
| C | 3.007059000 | 0.194329000 | -0.072255000 |
| C | -0.116499000 | 2.393812000 | -0.016126000 |
| C | 0.115859000 | -2.301373000 | -0.149607000 |
| C | 0.556973000 | -3.613907000 | -0.216606000 |
| C | -0.354788000 | -4.653078000 | -0.250194000 |
| C | -1.708131000 | -4.361016000 | -0.216116000 |
| C | -2.100055000 | -3.038643000 | -0.142530000 |
| N | -1.424725000 | 1.985580000 | -0.003841000 |
| N | -1.227622000 | -2.028217000 | -0.105942000 |
| C | -2.399528000 | 2.896965000 | 0.005384000 |
| C | -2.143585000 | 4.254708000 | 0.021980000 |
| C | -0.827260000 | 4.685016000 | 0.026572000 |
| C | 0.188061000 | 3.745704000 | 0.003660000 |
| C | -3.780666000 | -0.081529000 | 0.146928000 |

| | | | |
|---|---|---|---|
| O | -4.599536000 | 0.459991000 | -0.571658000 |
| O | -4.333050000 | -0.779673000 | 1.208857000 |
| C | 4.466169000 | 0.282982000 | -0.053902000 |
| C | 5.264654000 | 1.310921000 | -0.487781000 |
| C | 6.645837000 | 1.061789000 | -0.308526000 |
| C | 6.889528000 | -0.153136000 | 0.261418000 |
| S | 5.437613000 | -0.989720000 | 0.584354000 |
| H | 2.765812000 | 2.325054000 | 0.038854000 |
| H | 2.977886000 | -1.952074000 | -0.155413000 |
| H | 1.620500000 | -3.811263000 | -0.247910000 |
| H | -0.011894000 | -5.678966000 | -0.305526000 |
| H | -2.459757000 | -5.138187000 | -0.244220000 |
| H | -3.145497000 | -2.764238000 | -0.108562000 |
| H | -3.407709000 | 2.504295000 | -0.012280000 |
| H | -2.970021000 | 4.952397000 | 0.030788000 |
| H | -0.591789000 | 5.742164000 | 0.042092000 |
| H | 1.225493000 | 4.053251000 | -0.004301000 |
| H | -3.600819000 | -1.107199000 | 1.744248000 |
| H | 4.871443000 | 2.209575000 | -0.944909000 |
| H | 7.427653000 | 1.751737000 | -0.597583000 |
| H | 7.839978000 | -0.600935000 | 0.508778000 |

Sum of electronic and zero-point Energies= -1585.047561
Sum of electronic and thermal Energies= -1585.025450
Sum of electronic and thermal Enthalpies= -1585.024506
Sum of electronic and thermal Free Energies= -1585.100969

**3_ImE**

| | | | |
|---|---|---|---|
| Pt | -1.675216000 | -0.040517000 | -0.019315000 |
| C | -2.367851000 | 2.911063000 | 0.027356000 |
| C | -2.106034000 | 4.266248000 | 0.041285000 |
| C | -0.787434000 | 4.689077000 | 0.029886000 |
| C | 0.220914000 | 3.743420000 | 0.000409000 |
| C | -0.092503000 | 2.394022000 | -0.014325000 |
| N | -1.400320000 | 1.990910000 | 0.004083000 |
| C | 0.876900000 | 1.301384000 | -0.055221000 |
| C | 2.264255000 | 1.371572000 | -0.042772000 |
| C | 3.023893000 | 0.193133000 | -0.076017000 |
| C | 2.384278000 | -1.053626000 | -0.109055000 |
| C | 0.996113000 | -1.121848000 | -0.104797000 |
| C | 0.265784000 | 0.056380000 | -0.085036000 |
| C | -2.072191000 | -3.048163000 | -0.099795000 |
| C | -1.676761000 | -4.369686000 | -0.149175000 |
| C | -0.322673000 | -4.658194000 | -0.180556000 |
| C | 0.586242000 | -3.616333000 | -0.164812000 |
| C | 0.139881000 | -2.305587000 | -0.117617000 |
| N | -1.201681000 | -2.035515000 | -0.081567000 |
| C | -3.724065000 | -0.132013000 | 0.146155000 |
| O | -4.538475000 | 0.049503000 | -0.845569000 |
| O | -4.373048000 | -0.359424000 | 1.256817000 |
| H | -3.383817000 | 2.542925000 | 0.031797000 |
| H | -2.929293000 | 4.967108000 | 0.060727000 |
| H | -0.545858000 | 5.744661000 | 0.041279000 |

| | | | |
|---|---|---|---|
| H | 1.260050000 | 4.043873000 | -0.014360000 |
| H | 2.778164000 | 2.323902000 | 0.015861000 |
| H | 2.988618000 | -1.953517000 | -0.147908000 |
| H | -3.120077000 | -2.784758000 | -0.075315000 |
| H | -2.426367000 | -5.148978000 | -0.162718000 |
| H | 0.022826000 | -5.683805000 | -0.218907000 |
| H | 1.650186000 | -3.810799000 | -0.191937000 |
| H | -5.471617000 | -0.018839000 | -0.567105000 |
| H | -3.742387000 | -0.480998000 | 1.981377000 |
| C | 4.482594000 | 0.281387000 | -0.067884000 |
| C | 5.276155000 | 1.311469000 | -0.505663000 |
| S | 5.458107000 | -0.992784000 | 0.559191000 |
| C | 6.658135000 | 1.062390000 | -0.337598000 |
| H | 4.879775000 | 2.211309000 | -0.957654000 |
| C | 6.905610000 | -0.153996000 | 0.228153000 |
| H | 7.437800000 | 1.753090000 | -0.630093000 |
| H | 7.857892000 | -0.601327000 | 0.469332000 |

Sum of electronic and zero-point Energies= -1585.468868
Sum of electronic and thermal Energies= -1585.446609
Sum of electronic and thermal Enthalpies= -1585.445665
Sum of electronic and thermal Free Energies= -1585.522811

**3_TS**

| | | | |
|---|---|---|---|
| Pt | -1.667380000 | -0.050996000 | -0.016522000 |
| C | 2.267321000 | 1.377094000 | 0.032380000 |
| C | 0.880274000 | 1.301505000 | 0.022999000 |
| C | 0.275311000 | 0.054342000 | 0.046423000 |
| C | 1.007818000 | -1.121436000 | 0.084962000 |
| C | 2.395110000 | -1.047889000 | 0.113542000 |
| C | 3.030371000 | 0.201400000 | 0.084325000 |
| C | -0.092169000 | 2.391251000 | -0.037667000 |
| C | 0.156846000 | -2.309636000 | 0.086581000 |
| C | 0.608530000 | -3.618108000 | 0.126628000 |
| C | -0.296721000 | -4.663920000 | 0.115828000 |
| C | -1.650681000 | -4.380824000 | 0.060363000 |
| C | -2.052253000 | -3.060184000 | 0.021169000 |
| N | -1.399201000 | 1.986049000 | -0.062247000 |
| N | -1.184949000 | -2.045883000 | 0.036800000 |
| C | -2.366706000 | 2.904180000 | -0.122251000 |
| C | -2.105131000 | 4.259399000 | -0.160425000 |
| C | -0.787888000 | 4.684186000 | -0.134072000 |
| C | 0.220662000 | 3.739935000 | -0.071660000 |
| C | -3.684634000 | -0.201612000 | -0.109149000 |
| H | 2.778427000 | 2.331052000 | -0.021970000 |
| H | 3.001712000 | -1.945224000 | 0.169270000 |
| H | 1.672777000 | -3.808742000 | 0.165712000 |
| H | 0.052598000 | -5.688417000 | 0.148811000 |
| H | -2.396669000 | -5.163589000 | 0.047206000 |
| H | -3.099601000 | -2.799405000 | -0.029502000 |
| H | -3.381891000 | 2.535405000 | -0.137208000 |
| H | -2.928653000 | 4.958439000 | -0.210378000 |

| | | | |
|---|---|---|---|
| H | -0.546799000 | 5.739558000 | -0.162207000 |
| H | 1.259343000 | 4.041416000 | -0.049713000 |
| O | -4.528419000 | -0.820622000 | -0.769475000 |
| H | -5.298805000 | -0.214173000 | 0.100649000 |
| O | -4.618237000 | 0.541403000 | 0.763406000 |
| H | -4.451643000 | 0.474235000 | 1.719331000 |
| C | 4.488427000 | 0.294253000 | 0.100765000 |
| C | 5.271047000 | 1.332096000 | 0.540292000 |
| S | 5.478512000 | -0.984986000 | -0.492179000 |
| C | 6.656275000 | 1.084772000 | 0.400970000 |
| H | 4.864421000 | 2.236461000 | 0.973699000 |
| C | 6.917040000 | -0.137950000 | -0.145114000 |
| H | 7.428695000 | 1.781168000 | 0.699027000 |
| H | 7.874742000 | -0.585302000 | -0.363782000 |

Sum of electronic and zero-point Energies= -1585.408450
Sum of electronic and thermal Energies= -1585.386198
Sum of electronic and thermal Enthalpies= -1585.385254
Sum of electronic and thermal Free Energies= -1585.461943

**3_ImF**

| | | | |
|---|---|---|---|
| C | -2.079743000 | -1.385511000 | 0.037621000 |
| C | -0.694560000 | -1.313748000 | 0.032689000 |
| C | -0.087643000 | -0.052891000 | 0.048059000 |
| C | -0.831782000 | 1.131539000 | 0.068414000 |
| C | -2.216615000 | 1.044711000 | 0.085478000 |
| C | -2.848156000 | -0.209364000 | 0.069434000 |
| C | 0.267578000 | -2.392445000 | -0.006922000 |
| C | 0.001848000 | 2.312242000 | 0.071261000 |
| C | -0.443388000 | 3.627234000 | 0.101042000 |
| C | 0.454312000 | 4.674455000 | 0.100570000 |
| C | 1.820732000 | 4.380371000 | 0.068758000 |
| C | 2.222835000 | 3.069392000 | 0.039183000 |
| N | 1.582919000 | -1.964116000 | -0.025825000 |
| N | 1.357363000 | 2.035846000 | 0.039278000 |
| C | 2.559418000 | -2.892876000 | -0.060048000 |
| C | 2.307603000 | -4.241157000 | -0.078109000 |
| C | 0.983338000 | -4.688137000 | -0.060057000 |
| C | -0.026615000 | -3.749201000 | -0.023722000 |
| C | -4.304810000 | -0.306827000 | 0.081017000 |
| C | -5.091219000 | -1.352854000 | 0.497004000 |
| C | -6.476954000 | -1.104350000 | 0.357722000 |
| C | -6.739835000 | 0.128242000 | -0.163569000 |
| S | -5.298189000 | 0.982608000 | -0.490887000 |
| H | -2.587914000 | -2.342142000 | -0.005169000 |
| H | -2.827729000 | 1.940511000 | 0.125816000 |
| H | -1.509810000 | 3.813560000 | 0.125175000 |
| H | 0.107036000 | 5.699476000 | 0.124735000 |
| H | 2.569089000 | 5.161641000 | 0.066871000 |
| H | 3.272786000 | 2.812128000 | 0.014301000 |
| H | 3.573619000 | -2.518210000 | -0.072996000 |
| H | 3.139137000 | -4.932666000 | -0.105792000 |

| | | | |
|---|---|---|---|
| H | 0.753687000 | -5.745938000 | -0.073517000 |
| H | -1.065175000 | -4.054879000 | -0.007120000 |
| H | -4.685890000 | -2.265336000 | 0.914179000 |
| H | -7.248787000 | -1.808061000 | 0.640766000 |
| H | -7.697637000 | 0.580116000 | -0.371881000 |
| Pt | 1.842863000 | 0.056907000 | 0.001000000 |
| C | 3.776746000 | 0.169923000 | -0.064276000 |
| O | 4.916849000 | 0.238087000 | -0.112830000 |

Sum of electronic and zero-point Energies= -1509.247660
Sum of electronic and thermal Energies= -1509.226758
Sum of electronic and thermal Enthalpies= -1509.225814
Sum of electronic and thermal Free Energies= -1509.299882

**3_ImG**

| | | | |
|---|---|---|---|
| C | -2.067246000 | -1.400595000 | 0.051502000 |
| C | -0.685079000 | -1.328595000 | 0.057965000 |
| C | -0.070788000 | -0.055502000 | 0.090921000 |
| C | -0.833980000 | 1.134248000 | 0.085148000 |
| C | -2.215105000 | 1.035770000 | 0.084135000 |
| C | -2.845585000 | -0.224485000 | 0.071828000 |
| C | 0.268137000 | -2.398891000 | 0.008018000 |
| C | -0.018078000 | 2.312787000 | 0.070194000 |
| C | -0.460978000 | 3.631775000 | 0.099032000 |
| C | 0.425235000 | 4.685670000 | 0.081443000 |
| C | 1.800541000 | 4.385559000 | 0.033709000 |
| C | 2.207417000 | 3.081441000 | 0.006981000 |
| N | 1.591603000 | -1.949561000 | -0.030736000 |
| N | 1.350319000 | 2.028280000 | 0.022606000 |
| C | 2.569982000 | -2.890454000 | -0.074133000 |
| C | 2.323980000 | -4.234520000 | -0.082703000 |
| C | 0.995476000 | -4.700103000 | -0.043490000 |
| C | -0.011691000 | -3.761847000 | 0.002771000 |
| C | -4.297410000 | -0.327120000 | 0.062191000 |
| C | -5.093640000 | -1.410912000 | 0.356447000 |
| C | -6.478222000 | -1.149411000 | 0.228827000 |
| C | -6.742380000 | 0.130290000 | -0.160946000 |
| S | -5.294359000 | 1.016088000 | -0.381205000 |
| H | -2.569840000 | -2.360630000 | 0.002054000 |
| H | -2.830517000 | 1.930625000 | 0.101897000 |
| H | -1.529649000 | 3.809759000 | 0.136676000 |
| H | 0.076165000 | 5.710063000 | 0.105116000 |
| H | 2.549217000 | 5.167553000 | 0.016753000 |
| H | 3.259096000 | 2.829432000 | -0.029366000 |
| H | 3.583326000 | -2.512143000 | -0.103005000 |
| H | 3.161757000 | -4.919391000 | -0.120174000 |
| H | 0.772983000 | -5.759470000 | -0.047888000 |
| H | -1.050808000 | -4.068487000 | 0.036964000 |
| H | -4.694840000 | -2.364148000 | 0.677217000 |
| H | -7.250567000 | -1.881456000 | 0.428042000 |
| H | -7.699143000 | 0.601722000 | -0.325387000 |
| Pt | 1.844337000 | 0.061758000 | 0.009919000 |
| C | 3.760404000 | 0.180167000 | -0.074890000 |

| | | | |
|---|---|---|---|
| O | 4.911863000 | 0.252420000 | -0.153260000 |

Sum of electronic and zero-point Energies= -1509.337601
Sum of electronic and thermal Energies= -1509.316530
Sum of electronic and thermal Enthalpies= -1509.315586
Sum of electronic and thermal Free Energies= -1509.389467

**4_$S_0$**

| | | | |
|---|---|---|---|
| Pt | 2.918305000 | -0.000030000 | -0.000001000 |
| Cl | 5.343221000 | -0.000062000 | 0.000046000 |
| C | -1.035720000 | 1.203009000 | -0.199953000 |
| C | 0.351040000 | 1.204385000 | -0.207993000 |
| C | 1.022231000 | -0.000011000 | -0.000055000 |
| C | 0.351005000 | -1.204377000 | 0.207886000 |
| C | -1.035765000 | -1.202969000 | 0.199805000 |
| C | -1.728233000 | 0.000028000 | -0.000088000 |
| C | 1.267715000 | 2.323375000 | -0.412612000 |
| C | 1.267643000 | -2.323384000 | 0.412579000 |
| C | 0.903225000 | -3.639456000 | 0.645920000 |
| C | 1.880749000 | -4.603006000 | 0.820561000 |
| C | 3.214537000 | -4.232845000 | 0.758858000 |
| C | 3.527460000 | -2.907951000 | 0.523567000 |
| N | 2.588731000 | 1.976233000 | -0.354486000 |
| N | 2.588674000 | -1.976279000 | 0.354493000 |
| C | 3.527549000 | 2.907890000 | -0.523472000 |
| C | 3.214670000 | 4.232806000 | -0.758700000 |
| C | 1.880894000 | 4.603007000 | -0.820425000 |
| C | 0.903339000 | 3.639471000 | -0.645880000 |
| H | -1.603752000 | 2.108710000 | -0.376702000 |
| H | -1.603824000 | -2.108651000 | 0.376537000 |
| H | -0.145805000 | -3.901441000 | 0.688929000 |
| H | 1.602519000 | -5.633728000 | 1.002935000 |
| H | 4.010031000 | -4.953898000 | 0.889604000 |
| H | 4.549303000 | -2.554623000 | 0.463231000 |
| H | 4.549381000 | 2.554535000 | -0.463111000 |
| H | 4.010189000 | 4.953842000 | -0.889383000 |
| H | 1.602697000 | 5.633749000 | -1.002733000 |
| H | -0.145683000 | 3.901486000 | -0.688910000 |
| F | -7.012763000 | 0.728838000 | -0.870297000 |
| F | -7.011893000 | -0.729603000 | 0.871306000 |
| B | -6.209310000 | -0.000196000 | 0.000257000 |
| N | -5.279378000 | 0.944243000 | 0.797498000 |
| N | -5.279393000 | -0.944179000 | -0.797521000 |
| C | -3.440458000 | 1.854947000 | 1.739053000 |
| C | -4.559004000 | 2.464795000 | 2.280478000 |
| C | -5.672588000 | 1.869922000 | 1.676832000 |
| C | -5.672651000 | -1.869550000 | -1.677164000 |
| C | -4.559101000 | -2.464168000 | -2.281109000 |
| C | -3.440527000 | -1.854501000 | -1.739537000 |
| C | -3.193641000 | 0.000044000 | -0.000041000 |
| C | -3.896844000 | 0.907510000 | 0.799332000 |
| C | -3.896867000 | -0.907423000 | -0.799436000 |

| | | | |
|---|---|---|---|
| H | -2.408588000 | 2.042512000 | 1.989839000 |
| H | -4.586233000 | 3.238513000 | 3.031222000 |
| H | -6.721838000 | 2.060430000 | 1.848021000 |
| H | -6.721904000 | -2.060057000 | -1.848321000 |
| H | -4.586355000 | -3.237626000 | -3.032121000 |
| H | -2.408656000 | -2.041906000 | -1.990453000 |

Sum of electronic and zero-point Energies= -1984.212650
Sum of electronic and thermal Energies= -1984.186059
Sum of electronic and thermal Enthalpies= -1984.185115
Sum of electronic and thermal Free Energies= -1984.271268

**4_T$_1$**

| | | | |
|---|---|---|---|
| Pt | 2.932722000 | -0.000115000 | 0.000056000 |
| Cl | 5.361752000 | -0.000396000 | 0.000040000 |
| C | -1.027941000 | 1.195099000 | -0.219822000 |
| C | 0.361547000 | 1.201206000 | -0.212963000 |
| C | 1.033190000 | 0.000098000 | -0.000009000 |
| C | 0.361262000 | -1.200864000 | 0.212934000 |
| C | -1.028209000 | -1.194418000 | 0.219786000 |
| C | -1.727679000 | 0.000436000 | -0.000017000 |
| C | 1.277859000 | 2.320034000 | -0.416470000 |
| C | 1.277317000 | -2.319889000 | 0.416542000 |
| C | 0.912437000 | -3.636188000 | 0.651434000 |
| C | 1.888479000 | -4.600906000 | 0.826212000 |
| C | 3.223037000 | -4.232882000 | 0.763032000 |
| C | 3.536985000 | -2.908600000 | 0.526107000 |
| N | 2.600144000 | 1.975197000 | -0.356754000 |
| N | 2.599680000 | -1.975341000 | 0.356919000 |
| C | 3.537663000 | 2.908256000 | -0.525857000 |
| C | 3.224022000 | 4.232612000 | -0.762772000 |
| C | 1.889548000 | 4.600927000 | -0.826053000 |
| C | 0.913285000 | 3.636416000 | -0.651380000 |
| H | -1.592100000 | 2.101485000 | -0.411294000 |
| H | -1.592624000 | -2.100675000 | 0.411184000 |
| H | -0.137040000 | -3.896348000 | 0.695284000 |
| H | 1.608911000 | -5.631117000 | 1.009822000 |
| H | 4.017705000 | -4.954872000 | 0.893765000 |
| H | 4.559297000 | -2.556644000 | 0.464467000 |
| H | 4.559892000 | 2.556074000 | -0.464155000 |
| H | 4.018855000 | 4.954433000 | -0.893429000 |
| H | 1.610221000 | 5.631201000 | -1.009673000 |
| H | -0.136130000 | 3.896805000 | -0.695320000 |
| F | -7.039176000 | -0.749636000 | 0.852934000 |
| F | -7.041810000 | 0.747033000 | -0.851041000 |
| B | -6.228952000 | -0.000571000 | 0.000298000 |
| N | -5.297689000 | -0.917557000 | -0.810602000 |
| N | -5.298073000 | 0.918057000 | 0.809804000 |
| C | -3.469880000 | -1.867866000 | -1.758807000 |
| C | -4.583528000 | -2.462806000 | -2.299398000 |
| C | -5.705114000 | -1.860231000 | -1.703045000 |
| C | -5.706019000 | 1.860876000 | 1.701833000 |

| | | | |
|---|---|---|---|
| C | -4.584784000 | 2.464004000 | 2.298277000 |
| C | -3.470807000 | 1.869216000 | 1.758188000 |
| C | -3.196761000 | 0.000445000 | -0.000035000 |
| C | -3.930927000 | -0.889470000 | -0.807845000 |
| C | -3.931276000 | 0.890372000 | 0.807419000 |
| H | -2.435883000 | -2.061460000 | -1.993374000 |
| H | -4.615605000 | -3.239456000 | -3.047186000 |
| H | -6.754712000 | -2.045731000 | -1.872072000 |
| H | -6.755719000 | 2.046214000 | 1.870407000 |
| H | -4.617282000 | 3.240923000 | 3.045767000 |
| H | -2.436950000 | 2.063352000 | 1.992918000 |

Sum of electronic and zero-point Energies= -1984.154943
Sum of electronic and thermal Energies= -1984.127910
Sum of electronic and thermal Enthalpies= -1984.126966
Sum of electronic and thermal Free Energies= -1984.215214

**4_OER**

| | | | |
|---|---|---|---|
| Pt | 2.941361000 | 0.000052000 | -0.000002000 |
| Cl | 5.377815000 | 0.000306000 | 0.000652000 |
| C | 3.540262000 | 2.913853000 | -0.511518000 |
| C | 3.224762000 | 4.238886000 | -0.740625000 |
| C | 1.888910000 | 4.604681000 | -0.800790000 |
| C | 0.914843000 | 3.637669000 | -0.631366000 |
| C | 1.280993000 | 2.319402000 | -0.404566000 |
| N | 2.604815000 | 1.977625000 | -0.347464000 |
| C | 0.366119000 | 1.199544000 | -0.206704000 |
| C | -1.023611000 | 1.193902000 | -0.211989000 |
| C | -1.733837000 | -0.000287000 | -0.000130000 |
| C | -1.023472000 | -1.194368000 | 0.211662000 |
| C | 0.366288000 | -1.199829000 | 0.206361000 |
| C | 1.040380000 | -0.000117000 | -0.000205000 |
| C | 3.540648000 | -2.913711000 | 0.511162000 |
| C | 3.225329000 | -4.238812000 | 0.740137000 |
| C | 1.889529000 | -4.604802000 | 0.800216000 |
| C | 0.915330000 | -3.637910000 | 0.630844000 |
| C | 1.281298000 | -2.319579000 | 0.404141000 |
| N | 2.605080000 | -1.977602000 | 0.347133000 |
| F | -7.046105000 | 0.657809000 | -0.922965000 |
| F | -7.045793000 | -0.657208000 | 0.923552000 |
| B | -6.205541000 | 0.000275000 | 0.000118000 |
| N | -5.299618000 | 1.000744000 | 0.715899000 |
| N | -5.299991000 | -1.000332000 | -0.715942000 |
| C | -3.489366000 | 2.013694000 | 1.575431000 |
| C | -4.637946000 | 2.656836000 | 2.068310000 |
| C | -5.728980000 | 2.009084000 | 1.523136000 |
| C | -5.729879000 | -2.008824000 | -1.522729000 |
| C | -4.639183000 | -2.657237000 | -2.067747000 |
| C | -3.490269000 | -2.014445000 | -1.575174000 |
| C | -3.197125000 | -0.000252000 | -0.000023000 |
| C | -3.920191000 | 0.982658000 | 0.726794000 |
| C | -3.920550000 | -0.982933000 | -0.726867000 |

| | | | |
|---|---|---|---|
| H | 4.563191000 | 2.563103000 | -0.451901000 |
| H | 4.018174000 | 4.963031000 | -0.867293000 |
| H | 1.607334000 | 5.635595000 | -0.977896000 |
| H | -0.135468000 | 3.895119000 | -0.672007000 |
| H | -1.586889000 | 2.101093000 | -0.397123000 |
| H | -1.586583000 | -2.101598000 | 0.397051000 |
| H | 4.563530000 | -2.562808000 | 0.451655000 |
| H | 4.018840000 | -4.962850000 | 0.866792000 |
| H | 1.608090000 | -5.635772000 | 0.977214000 |
| H | -0.134945000 | -3.895517000 | 0.671424000 |
| H | -2.463887000 | 2.247581000 | 1.814543000 |
| H | -4.672135000 | 3.496364000 | 2.746741000 |
| H | -6.783706000 | 2.192680000 | 1.660739000 |
| H | -6.784709000 | -2.191820000 | -1.660320000 |
| H | -4.673736000 | -3.496923000 | -2.745960000 |
| H | -2.464974000 | -2.249009000 | -1.814372000 |

Sum of electronic and zero-point Energies= -1984.339860
Sum of electronic and thermal Energies= -1984.313013
Sum of electronic and thermal Enthalpies= -1984.312069
Sum of electronic and thermal Free Energies= -1984.399234

**4_ImA**

| | | | |
|---|---|---|---|
| Pt | 3.227529000 | -0.000018000 | 0.000015000 |
| C | 3.853387000 | 2.914218000 | -0.507742000 |
| C | 3.542023000 | 4.240387000 | -0.733916000 |
| C | 2.206642000 | 4.608220000 | -0.792322000 |
| C | 1.229497000 | 3.643462000 | -0.624315000 |
| C | 1.595008000 | 2.325729000 | -0.400713000 |
| N | 2.914758000 | 1.981163000 | -0.345338000 |
| C | 0.684932000 | 1.203419000 | -0.203302000 |
| C | -0.704188000 | 1.194154000 | -0.206971000 |
| C | -1.416137000 | -0.000034000 | -0.000109000 |
| C | -0.704226000 | -1.194182000 | 0.206729000 |
| C | 0.684934000 | -1.203447000 | 0.203144000 |
| C | 1.349547000 | -0.000029000 | -0.000047000 |
| C | 3.853311000 | -2.914333000 | 0.507889000 |
| C | 3.541884000 | -4.240487000 | 0.734088000 |
| C | 2.206487000 | -4.608272000 | 0.792379000 |
| C | 1.229390000 | -3.643486000 | 0.624244000 |
| C | 1.594970000 | -2.325771000 | 0.400630000 |
| N | 2.914739000 | -1.981252000 | 0.345365000 |
| F | -6.722658000 | 0.639565000 | -0.934846000 |
| F | -6.720837000 | -0.640398000 | 0.936634000 |
| B | -5.884317000 | -0.000065000 | 0.000300000 |
| N | -4.977211000 | 1.014643000 | 0.696828000 |
| N | -4.977335000 | -1.014084000 | -0.697381000 |
| C | -3.168230000 | 2.039940000 | 1.543476000 |
| C | -4.316587000 | 2.690400000 | 2.025035000 |
| C | -5.407001000 | 2.034285000 | 1.488018000 |
| C | -5.407326000 | -2.033644000 | -1.488540000 |
| C | -4.317036000 | -2.690033000 | -2.025490000 |

| | | | |
|---|---|---|---|
| C | -3.168544000 | -2.039847000 | -1.543870000 |
| C | -2.876182000 | 0.000019000 | -0.000155000 |
| C | -3.598442000 | 0.996754000 | 0.709060000 |
| C | -3.598544000 | -0.996565000 | -0.709472000 |
| H | 4.879533000 | 2.573555000 | -0.451624000 |
| H | 4.336527000 | 4.963181000 | -0.859554000 |
| H | 1.927675000 | 5.640015000 | -0.967042000 |
| H | 0.180062000 | 3.903860000 | -0.663835000 |
| H | -1.263797000 | 2.103126000 | -0.391397000 |
| H | -1.263787000 | -2.103190000 | 0.391113000 |
| H | 4.879478000 | -2.573719000 | 0.451853000 |
| H | 4.336355000 | -4.963298000 | 0.859833000 |
| H | 1.927466000 | -5.640050000 | 0.967118000 |
| H | 0.179945000 | -3.903853000 | 0.663679000 |
| H | -2.143853000 | 2.276839000 | 1.784233000 |
| H | -4.351563000 | 3.539560000 | 2.691136000 |
| H | -6.461798000 | 2.219630000 | 1.622308000 |
| H | -6.462162000 | -2.218667000 | -1.622954000 |
| H | -4.352150000 | -3.539115000 | -2.691677000 |
| H | -2.144253000 | -2.277066000 | -1.784644000 |

Sum of electronic and zero-point Energies= -1524.070256
Sum of electronic and thermal Energies= -1524.045258
Sum of electronic and thermal Enthalpies= -1524.044314
Sum of electronic and thermal Free Energies= -1524.127411

**4_ImB**

| | | | |
|---|---|---|---|
| Pt | 3.254146000 | -0.000103000 | 0.000211000 |
| C | 3.865738000 | 2.941499000 | -0.391206000 |
| C | 3.548927000 | 4.271854000 | -0.567486000 |
| C | 2.209688000 | 4.638726000 | -0.618254000 |
| C | 1.239515000 | 3.660483000 | -0.490156000 |
| C | 1.606660000 | 2.334849000 | -0.317560000 |
| N | 2.933421000 | 1.989777000 | -0.269741000 |
| C | 0.699379000 | 1.206407000 | -0.160041000 |
| C | -0.685067000 | 1.206432000 | -0.158851000 |
| C | -1.434451000 | 0.000102000 | -0.000730000 |
| C | -0.685229000 | -1.206313000 | 0.157433000 |
| C | 0.699209000 | -1.206499000 | 0.158558000 |
| C | 1.371500000 | -0.000101000 | -0.000894000 |
| C | 3.865317000 | -2.941722000 | 0.392160000 |
| C | 3.548316000 | -4.272061000 | 0.568189000 |
| C | 2.209016000 | -4.638867000 | 0.617999000 |
| C | 1.238989000 | -3.660577000 | 0.489203000 |
| C | 1.606315000 | -2.334960000 | 0.316846000 |
| N | 2.933141000 | -1.989951000 | 0.270017000 |
| F | -6.744206000 | 0.399785000 | -1.059295000 |
| F | -6.743694000 | -0.399532000 | 1.060616000 |
| B | -5.887130000 | 0.000111000 | 0.000454000 |
| N | -4.992281000 | 1.144315000 | 0.435163000 |
| N | -4.992525000 | -1.144108000 | -0.434716000 |
| C | -3.221771000 | 2.348118000 | 1.071882000 |

| | | | |
|---|---|---|---|
| C | -4.394542000 | 3.065593000 | 1.400951000 |
| C | -5.461431000 | 2.301601000 | 0.996932000 |
| C | -5.461915000 | -2.301387000 | -0.996287000 |
| C | -4.395207000 | -3.065324000 | -1.400899000 |
| C | -3.222316000 | -2.347676000 | -1.072681000 |
| C | -2.864402000 | 0.000186000 | -0.000455000 |
| C | -3.611506000 | 1.143129000 | 0.464980000 |
| C | -3.611780000 | -1.142756000 | -0.465493000 |
| H | 4.894410000 | 2.606656000 | -0.342526000 |
| H | 4.341652000 | 5.001950000 | -0.661022000 |
| H | 1.926412000 | 5.675068000 | -0.755146000 |
| H | 0.187328000 | 3.912458000 | -0.520813000 |
| H | -1.223843000 | 2.125682000 | -0.341663000 |
| H | -1.224121000 | -2.125460000 | 0.340446000 |
| H | 4.894044000 | -2.606929000 | 0.344263000 |
| H | 4.340937000 | -5.002198000 | 0.662282000 |
| H | 1.925586000 | -5.675194000 | 0.754676000 |
| H | 0.186769000 | -3.912511000 | 0.519078000 |
| H | -2.215211000 | 2.658549000 | 1.297795000 |
| H | -4.447197000 | 4.035030000 | 1.875971000 |
| H | -6.522491000 | 2.484428000 | 1.070868000 |
| H | -6.523000000 | -2.484334000 | -1.069585000 |
| H | -4.448105000 | -4.034814000 | -1.875785000 |
| H | -2.215827000 | -2.657804000 | -1.299361000 |

Sum of electronic and zero-point Energies= -1524.159229
Sum of electronic and thermal Energies= -1524.133946
Sum of electronic and thermal Enthalpies= -1524.133002
Sum of electronic and thermal Free Energies= -1524.216832

**4_ImC**

| | | | |
|---|---|---|---|
| Pt | -2.870260000 | 0.006551000 | -0.011173000 |
| C | -3.423379000 | -2.921367000 | -0.518930000 |
| C | -3.094560000 | -4.240938000 | -0.778856000 |
| C | -1.760353000 | -4.594297000 | -0.883489000 |
| C | -0.793121000 | -3.616564000 | -0.724036000 |
| C | -1.166624000 | -2.306628000 | -0.469680000 |
| N | -2.493442000 | -1.975803000 | -0.370319000 |
| C | -0.237404000 | -1.186965000 | -0.284135000 |
| C | 1.148974000 | -1.198772000 | -0.261956000 |
| C | 1.844791000 | 0.005252000 | -0.053871000 |
| C | 1.154070000 | 1.214730000 | 0.138122000 |
| C | -0.232808000 | 1.213435000 | 0.128052000 |
| C | -0.909044000 | 0.016776000 | -0.091031000 |
| C | -3.413161000 | 2.936852000 | 0.491523000 |
| C | -3.079007000 | 4.263300000 | 0.705574000 |
| C | -1.743087000 | 4.624070000 | 0.740339000 |
| C | -0.779489000 | 3.646817000 | 0.557916000 |
| C | -1.157954000 | 2.331052000 | 0.344071000 |
| N | -2.486379000 | 1.992634000 | 0.316965000 |
| F | 7.141663000 | -0.728489000 | -0.819405000 |
| F | 7.117410000 | 0.662527000 | 0.975592000 |

| | | | |
|---|---|---|---|
| B | 6.323548000 | -0.024528000 | 0.060801000 |
| N | 5.369174000 | -0.987412000 | 0.801053000 |
| N | 5.422121000 | 0.957564000 | -0.719869000 |
| C | 3.503932000 | -1.908324000 | 1.674469000 |
| C | 4.606215000 | -2.540654000 | 2.226549000 |
| C | 5.736531000 | -1.938543000 | 1.665961000 |
| C | 5.847133000 | 1.909973000 | -1.556530000 |
| C | 4.756110000 | 2.529196000 | -2.173521000 |
| C | 3.618010000 | 1.906352000 | -1.687002000 |
| C | 3.305736000 | -0.003325000 | -0.017808000 |
| C | 3.987287000 | -0.941564000 | 0.770059000 |
| C | 4.040176000 | 0.927999000 | -0.764979000 |
| H | -4.442240000 | -2.544056000 | -0.393544000 |
| H | -3.883450000 | -4.973022000 | -0.893470000 |
| H | -1.471522000 | -5.618931000 | -1.085839000 |
| H | 0.258558000 | -3.861894000 | -0.798341000 |
| H | 1.720809000 | -2.104845000 | -0.428575000 |
| H | 1.728479000 | 2.113287000 | 0.334464000 |
| H | -4.434933000 | 2.547160000 | 0.450650000 |
| H | -3.865208000 | 4.994472000 | 0.842411000 |
| H | -1.450218000 | 5.654156000 | 0.905878000 |
| H | 0.273251000 | 3.898058000 | 0.576472000 |
| H | 2.465442000 | -2.092789000 | 1.898154000 |
| H | 4.609658000 | -3.332979000 | 2.958212000 |
| H | 6.780407000 | -2.138115000 | 1.858120000 |
| H | 6.902185000 | 2.098616000 | -1.689596000 |
| H | 4.808813000 | 3.326994000 | -2.897294000 |
| H | 2.595183000 | 2.105666000 | -1.963941000 |
| C | -4.997485000 | -0.018710000 | 0.155553000 |
| O | -5.626934000 | 1.075564000 | 0.293754000 |
| O | -5.612265000 | -1.129342000 | 0.118624000 |

Sum of electronic and zero-point Energies= -1712.633109
Sum of electronic and thermal Energies= -1712.605009
Sum of electronic and thermal Enthalpies= -1712.604065
Sum of electronic and thermal Free Energies= -1712.693767

**4_ImD**

| | | | |
|---|---|---|---|
| Pt | -2.837339000 | 0.011578000 | -0.016152000 |
| C | -3.399197000 | -2.917363000 | -0.578126000 |
| C | -3.071856000 | -4.230902000 | -0.857442000 |
| C | -1.736307000 | -4.581264000 | -0.954133000 |
| C | -0.773153000 | -3.605355000 | -0.770945000 |
| C | -1.150194000 | -2.300362000 | -0.499040000 |
| N | -2.476576000 | -1.968701000 | -0.402227000 |
| C | -0.225828000 | -1.184316000 | -0.294688000 |
| C | 1.162022000 | -1.195857000 | -0.272959000 |
| C | 1.856099000 | 0.002095000 | -0.048186000 |
| C | 1.168513000 | 1.207052000 | 0.159566000 |
| C | -0.219381000 | 1.209358000 | 0.148776000 |
| C | -0.893657000 | 0.016096000 | -0.084482000 |
| C | -3.388387000 | 2.942166000 | 0.480112000 |

| | | | |
|---|---|---|---|
| C | -3.057243000 | 4.264702000 | 0.709385000 |
| C | -1.720511000 | 4.618613000 | 0.773857000 |
| C | -0.757630000 | 3.640481000 | 0.598071000 |
| C | -1.137168000 | 2.328309000 | 0.368209000 |
| N | -2.464435000 | 1.992840000 | 0.320993000 |
| F | 7.153104000 | -0.754810000 | -0.791583000 |
| F | 7.125774000 | 0.692693000 | 0.958898000 |
| B | 6.336544000 | -0.024145000 | 0.065523000 |
| N | 5.380929000 | -0.965497000 | 0.835418000 |
| N | 5.432961000 | 0.932925000 | -0.746863000 |
| C | 3.512639000 | -1.872621000 | 1.720967000 |
| C | 4.613075000 | -2.489725000 | 2.290571000 |
| C | 5.745539000 | -1.896957000 | 1.720969000 |
| C | 5.853642000 | 1.866082000 | -1.605299000 |
| C | 4.759234000 | 2.473019000 | -2.231664000 |
| C | 3.623816000 | 1.863044000 | -1.726678000 |
| C | 3.321841000 | -0.006795000 | -0.013227000 |
| C | 3.998980000 | -0.922642000 | 0.798998000 |
| C | 4.050733000 | 0.903090000 | -0.785942000 |
| H | -4.425860000 | -2.597271000 | -0.471240000 |
| H | -3.861368000 | -4.957930000 | -0.991813000 |
| H | -1.445490000 | -5.602136000 | -1.169530000 |
| H | 0.279200000 | -3.847537000 | -0.841034000 |
| H | 1.730635000 | -2.101884000 | -0.447165000 |
| H | 1.741226000 | 2.104822000 | 0.361424000 |
| H | -4.415191000 | 2.606268000 | 0.400983000 |
| H | -3.844705000 | 4.996027000 | 0.832510000 |
| H | -1.427913000 | 5.645874000 | 0.953786000 |
| H | 0.295127000 | 3.888445000 | 0.633641000 |
| H | 2.473281000 | -2.056383000 | 1.941787000 |
| H | 4.616031000 | -3.266959000 | 3.038152000 |
| H | 6.788774000 | -2.092946000 | 1.920354000 |
| H | 6.907915000 | 2.053671000 | -1.746055000 |
| H | 4.810079000 | 3.255090000 | -2.972430000 |
| H | 2.599514000 | 2.059064000 | -2.000977000 |
| C | -4.926950000 | 0.062348000 | 0.137640000 |
| O | -5.681746000 | 0.956944000 | -0.217283000 |
| O | -5.516991000 | -1.026136000 | 0.728339000 |
| H | -6.473744000 | -0.850722000 | 0.738466000 |

Sum of electronic and zero-point Energies= -1713.122645
Sum of electronic and thermal Energies= -1713.094206
Sum of electronic and thermal Enthalpies= -1713.093262
Sum of electronic and thermal Free Energies= -1713.183541

**4_ImE**

| | | | |
|---|---|---|---|
| Pt | -2.819620000 | 0.008363000 | -0.015733000 |
| C | -3.380126000 | -2.919393000 | -0.574858000 |
| C | -3.056196000 | -4.236484000 | -0.831824000 |
| C | -1.719946000 | -4.590996000 | -0.908489000 |
| C | -0.755306000 | -3.616160000 | -0.730155000 |
| C | -1.131099000 | -2.307457000 | -0.477152000 |

| | | | |
|---|---|---|---|
| N | -2.455429000 | -1.972448000 | -0.397536000 |
| C | -0.212557000 | -1.188323000 | -0.278295000 |
| C | 1.176173000 | -1.197518000 | -0.261687000 |
| C | 1.869270000 | 0.000604000 | -0.049551000 |
| C | 1.183186000 | 1.205338000 | 0.147338000 |
| C | -0.205721000 | 1.207718000 | 0.142058000 |
| C | -0.877325000 | 0.012653000 | -0.073998000 |
| C | -3.362670000 | 2.948733000 | 0.491268000 |
| C | -3.030707000 | 4.271405000 | 0.705380000 |
| C | -1.692364000 | 4.623928000 | 0.746778000 |
| C | -0.733986000 | 3.642862000 | 0.569261000 |
| C | -1.117753000 | 2.329590000 | 0.354675000 |
| N | -2.444127000 | 1.994218000 | 0.321547000 |
| F | 7.167896000 | -0.787253000 | -0.760654000 |
| F | 7.133268000 | 0.737995000 | 0.923142000 |
| B | 6.350344000 | -0.020633000 | 0.061152000 |
| N | 5.393943000 | -0.929567000 | 0.871241000 |
| N | 5.444786000 | 0.897599000 | -0.795542000 |
| C | 3.523946000 | -1.806360000 | 1.786301000 |
| C | 4.623256000 | -2.400166000 | 2.380055000 |
| C | 5.757044000 | -1.827255000 | 1.790486000 |
| C | 5.862375000 | 1.796404000 | -1.690084000 |
| C | 4.765430000 | 2.380585000 | -2.335845000 |
| C | 3.631951000 | 1.793005000 | -1.803387000 |
| C | 3.338163000 | -0.007935000 | -0.017332000 |
| C | 4.012061000 | -0.889292000 | 0.831310000 |
| C | 4.062400000 | 0.868032000 | -0.828578000 |
| H | -4.412069000 | -2.605388000 | -0.509698000 |
| H | -3.846716000 | -4.961907000 | -0.966685000 |
| H | -1.430604000 | -5.615501000 | -1.106775000 |
| H | 0.296545000 | -3.862647000 | -0.788141000 |
| H | 1.742560000 | -2.106078000 | -0.427835000 |
| H | 1.754239000 | 2.107139000 | 0.333509000 |
| H | -4.396697000 | 2.637213000 | 0.451599000 |
| H | -3.816820000 | 5.002470000 | 0.835457000 |
| H | -1.396724000 | 5.652299000 | 0.913152000 |
| H | 0.319320000 | 3.888885000 | 0.592276000 |
| H | 2.484206000 | -1.985102000 | 2.009928000 |
| H | 4.626298000 | -3.149898000 | 3.155158000 |
| H | 6.799905000 | -2.016669000 | 1.998182000 |
| H | 6.916090000 | 1.979206000 | -1.841138000 |
| H | 4.815071000 | 3.133783000 | -3.105964000 |
| H | 2.606579000 | 1.981187000 | -2.079647000 |
| C | -4.869996000 | -0.015745000 | 0.144740000 |
| O | -5.674714000 | 0.047630000 | -0.868261000 |
| H | -6.610617000 | 0.017660000 | -0.592184000 |
| O | -5.526749000 | -0.102905000 | 1.269731000 |
| H | -4.901896000 | -0.155535000 | 2.007602000 |

Sum of electronic and zero-point Energies= -1713.539472
Sum of electronic and thermal Energies= -1713.510690
Sum of electronic and thermal Enthalpies= -1713.509746

Sum of electronic and thermal Free Energies= -1713.602145

**4_TS**

| | | | |
|---|---|---|---|
| Pt | -2.814460000 | 0.001152000 | -0.037093000 |
| C | 1.186398000 | 1.205725000 | 0.134648000 |
| C | -0.202632000 | 1.205898000 | 0.123321000 |
| C | -0.869459000 | 0.007266000 | -0.082121000 |
| C | -0.204125000 | -1.195347000 | -0.268718000 |
| C | 1.184658000 | -1.202641000 | -0.243879000 |
| C | 1.874074000 | -0.000697000 | -0.043806000 |
| C | -1.116684000 | 2.330190000 | 0.317292000 |
| C | -1.119760000 | -2.317793000 | -0.464814000 |
| C | -0.740306000 | -3.627316000 | -0.704556000 |
| C | -1.703157000 | -4.603870000 | -0.886820000 |
| C | -3.039775000 | -4.248954000 | -0.829413000 |
| C | -3.368138000 | -2.929986000 | -0.584693000 |
| N | -2.442895000 | 1.995071000 | 0.282069000 |
| N | -2.444835000 | -1.983904000 | -0.401222000 |
| C | -3.361778000 | 2.951660000 | 0.433591000 |
| C | -3.029469000 | 4.277359000 | 0.630889000 |
| C | -1.691634000 | 4.629622000 | 0.675346000 |
| C | -0.732794000 | 3.645238000 | 0.516852000 |
| C | -4.838159000 | -0.060418000 | 0.077464000 |
| O | -5.782835000 | -0.537574000 | -0.562985000 |
| O | -5.618632000 | 0.545689000 | 1.170466000 |
| F | 7.176255000 | -0.798865000 | -0.722301000 |
| F | 7.133325000 | 0.767562000 | 0.923158000 |
| B | 6.354944000 | -0.013826000 | 0.077760000 |
| N | 5.397608000 | -0.904569000 | 0.907225000 |
| N | 5.450100000 | 0.881274000 | -0.804212000 |
| C | 3.525959000 | -1.763900000 | 1.836149000 |
| C | 4.624308000 | -2.342392000 | 2.446216000 |
| C | 5.759142000 | -1.781011000 | 1.847180000 |
| C | 5.868039000 | 1.758674000 | -1.719415000 |
| C | 4.771341000 | 2.327309000 | -2.379723000 |
| C | 3.637647000 | 1.752554000 | -1.834309000 |
| C | 3.343546000 | -0.006600000 | -0.007493000 |
| C | 4.015771000 | -0.867296000 | 0.862625000 |
| C | 4.067739000 | 0.850674000 | -0.837759000 |
| H | 1.755702000 | 2.110667000 | 0.310499000 |
| H | 1.753299000 | -2.112314000 | -0.395250000 |
| H | 0.312162000 | -3.873742000 | -0.749866000 |
| H | -1.411187000 | -5.629689000 | -1.074146000 |
| H | -3.828439000 | -4.975366000 | -0.969754000 |
| H | -4.400139000 | -2.613212000 | -0.537328000 |
| H | -4.395047000 | 2.638831000 | 0.396846000 |
| H | -3.815891000 | 5.010688000 | 0.745415000 |
| H | -1.395988000 | 5.659953000 | 0.829069000 |
| H | 0.320548000 | 3.890694000 | 0.542513000 |
| H | -6.412985000 | -0.068237000 | 0.489977000 |
| H | -5.337040000 | 0.323419000 | 2.074530000 |

| | | | |
|---|---|---|---|
| H | 2.485733000 | -1.939706000 | 2.060008000 |
| H | 4.626285000 | -3.074830000 | 3.237677000 |
| H | 6.801637000 | -1.964757000 | 2.061738000 |
| H | 6.921797000 | 1.938460000 | -1.873804000 |
| H | 4.821540000 | 3.062078000 | -3.167402000 |
| H | 2.612349000 | 1.934351000 | -2.115201000 |

Sum of electronic and zero-point Energies= -1713.478764
Sum of electronic and thermal Energies= -1713.449976
Sum of electronic and thermal Enthalpies= -1713.449031
Sum of electronic and thermal Free Energies= -1713.541211

**4_ImF**

| | | | |
|---|---|---|---|
| Pt | 2.995273000 | -0.004279000 | -0.000926000 |
| C | 3.550677000 | 2.930798000 | -0.538261000 |
| C | 3.223190000 | 4.249129000 | -0.785019000 |
| C | 1.886885000 | 4.601038000 | -0.860353000 |
| C | 0.922675000 | 3.623546000 | -0.688251000 |
| C | 1.301765000 | 2.315603000 | -0.444494000 |
| N | 2.625327000 | 1.983350000 | -0.370563000 |
| C | 0.387933000 | 1.192543000 | -0.246982000 |
| C | -1.001283000 | 1.197567000 | -0.238291000 |
| C | -1.690742000 | -0.001716000 | -0.029447000 |
| C | -1.005435000 | -1.204712000 | 0.171613000 |
| C | 0.383894000 | -1.205628000 | 0.172262000 |
| C | 1.047514000 | -0.008034000 | -0.039310000 |
| C | 3.541035000 | -2.945595000 | 0.511806000 |
| C | 3.209136000 | -4.267452000 | 0.732576000 |
| C | 1.871727000 | -4.620067000 | 0.779182000 |
| C | 0.910817000 | -3.640312000 | 0.601643000 |
| C | 1.294234000 | -2.329440000 | 0.381642000 |
| N | 2.618912000 | -1.995496000 | 0.341094000 |
| F | -6.979904000 | 0.802816000 | -0.775945000 |
| F | -6.963987000 | -0.768793000 | 0.865246000 |
| B | -6.172816000 | 0.013621000 | 0.033680000 |
| N | -5.225314000 | 0.900336000 | 0.879729000 |
| N | -5.256492000 | -0.881042000 | -0.838024000 |
| C | -3.364210000 | 1.760323000 | 1.830698000 |
| C | -4.469373000 | 2.338494000 | 2.427517000 |
| C | -5.597468000 | 1.776999000 | 1.814833000 |
| C | -5.661944000 | -1.758709000 | -1.758129000 |
| C | -4.556239000 | -2.329088000 | -2.402691000 |
| C | -3.430068000 | -1.755448000 | -1.841826000 |
| C | -3.162031000 | 0.003687000 | -0.011120000 |
| C | -3.843043000 | 0.862986000 | 0.851783000 |
| C | -3.873776000 | -0.851897000 | -0.852246000 |
| H | 4.583570000 | 2.620528000 | -0.471568000 |
| H | 4.012493000 | 4.976932000 | -0.913129000 |
| H | 1.595817000 | 5.626315000 | -1.051456000 |
| H | -0.129391000 | 3.869246000 | -0.742905000 |
| H | -1.567962000 | 2.105027000 | -0.407592000 |
| H | -1.574904000 | -2.108540000 | 0.350966000 |

| | | | |
|---|---|---|---|
| H | 4.575014000 | -2.634774000 | 0.468768000 |
| H | 3.996045000 | -4.997266000 | 0.863934000 |
| H | 1.577284000 | -5.647850000 | 0.950647000 |
| H | -0.142050000 | -3.886960000 | 0.630731000 |
| H | -2.326653000 | 1.937023000 | 2.066228000 |
| H | -4.480897000 | 3.071260000 | 3.218554000 |
| H | -6.642326000 | 1.962096000 | 2.016381000 |
| H | -6.713502000 | -1.938591000 | -1.926809000 |
| H | -4.596216000 | -3.064805000 | -3.190039000 |
| H | -2.401047000 | -1.939583000 | -2.107336000 |
| C | 4.954676000 | 0.015089000 | 0.097979000 |
| O | 6.080282000 | 0.032639000 | 0.179555000 |

Sum of electronic and zero-point Energies= -1637.193285
Sum of electronic and thermal Energies= -1637.166064
Sum of electronic and thermal Enthalpies= -1637.165120
Sum of electronic and thermal Free Energies= -1637.253109

**4_ImG**

| | | | |
|---|---|---|---|
| Pt | 3.017090000 | -0.004105000 | 0.000017000 |
| C | 3.563480000 | 2.939607000 | -0.507961000 |
| C | 3.234461000 | 4.259421000 | -0.739625000 |
| C | 1.896150000 | 4.609672000 | -0.810580000 |
| C | 0.934641000 | 3.629079000 | -0.649160000 |
| C | 1.314280000 | 2.316904000 | -0.420719000 |
| N | 2.639940000 | 1.987400000 | -0.351129000 |
| C | 0.403820000 | 1.191885000 | -0.233423000 |
| C | -0.983049000 | 1.195655000 | -0.231722000 |
| C | -1.698713000 | -0.001230000 | -0.030402000 |
| C | -0.987330000 | -1.201910000 | 0.163226000 |
| C | 0.399547000 | -1.203887000 | 0.158741000 |
| C | 1.072867000 | -0.007526000 | -0.039611000 |
| C | 3.553120000 | -2.952773000 | 0.491554000 |
| C | 3.219411000 | -4.275096000 | 0.701211000 |
| C | 1.879931000 | -4.625789000 | 0.741426000 |
| C | 0.921978000 | -3.643544000 | 0.569439000 |
| C | 1.306220000 | -2.329302000 | 0.361513000 |
| N | 2.633030000 | -1.998570000 | 0.326528000 |
| F | -7.005504000 | 0.625115000 | -0.918832000 |
| F | -6.989380000 | -0.590921000 | 0.995305000 |
| B | -6.163796000 | 0.013143000 | 0.028800000 |
| N | -5.242221000 | 1.046031000 | 0.680843000 |
| N | -5.267060000 | -1.028354000 | -0.643965000 |
| C | -3.420573000 | 2.082539000 | 1.484238000 |
| C | -4.559067000 | 2.746190000 | 1.965177000 |
| C | -5.658279000 | 2.081312000 | 1.454669000 |
| C | -5.710807000 | -2.065673000 | -1.399405000 |
| C | -4.630396000 | -2.738537000 | -1.938883000 |
| C | -3.474955000 | -2.077531000 | -1.496134000 |
| C | -3.152731000 | 0.004071000 | -0.013817000 |
| C | -3.863560000 | 1.024318000 | 0.675154000 |
| C | -3.888773000 | -1.013335000 | -0.679631000 |

| | | | |
|---|---|---|---|
| H | 4.596754000 | 2.629344000 | -0.444324000 |
| H | 4.022394000 | 4.990195000 | -0.859382000 |
| H | 1.603227000 | 5.636669000 | -0.989948000 |
| H | -0.118568000 | 3.871375000 | -0.698762000 |
| H | -1.541444000 | 2.105896000 | -0.410648000 |
| H | -1.548512000 | -2.108610000 | 0.351427000 |
| H | 4.587503000 | -2.642156000 | 0.452568000 |
| H | 4.004758000 | -5.007384000 | 0.828463000 |
| H | 1.583424000 | -5.654590000 | 0.903762000 |
| H | -0.132028000 | -3.886424000 | 0.592239000 |
| H | -2.393737000 | 2.319696000 | 1.712822000 |
| H | -4.583378000 | 3.608194000 | 2.614748000 |
| H | -6.710743000 | 2.271312000 | 1.600287000 |
| H | -6.768027000 | -2.251579000 | -1.512207000 |
| H | -4.678265000 | -3.603935000 | -2.582592000 |
| H | -2.456300000 | -2.320841000 | -1.753244000 |
| C | 4.968592000 | 0.011629000 | 0.088780000 |
| O | 6.098184000 | 0.025891000 | 0.161370000 |

Sum of electronic and zero-point Energies= -1637.332801
Sum of electronic and thermal Energies= -1637.305428
Sum of electronic and thermal Enthalpies= -1637.304484
Sum of electronic and thermal Free Energies= -1637.393159

**TEOA in DCM**

| | | | |
|---|---|---|---|
| N | -0.001770000 | -0.007522000 | 0.098609000 |
| C | -0.107718000 | 1.382146000 | 0.501972000 |
| C | -1.157847000 | -0.793388000 | 0.485333000 |
| C | 1.250450000 | -0.617101000 | 0.505450000 |
| C | -2.298788000 | -0.638513000 | -0.504183000 |
| C | 0.587408000 | 2.299813000 | -0.479778000 |
| O | 0.526280000 | 3.615232000 | 0.038820000 |
| O | -3.444870000 | -1.369940000 | -0.115122000 |
| C | 1.696687000 | -1.688769000 | -0.473476000 |
| O | 2.859595000 | -2.364236000 | -0.036407000 |
| H | -1.162400000 | 1.663614000 | 0.544221000 |
| H | 0.303634000 | 1.548591000 | 1.511197000 |
| H | -1.507574000 | -0.527647000 | 1.499107000 |
| H | -0.879778000 | -1.849058000 | 0.520733000 |
| H | 1.189048000 | -1.049373000 | 1.517947000 |
| H | 2.021584000 | 0.158110000 | 0.542581000 |
| H | -1.989968000 | -1.035492000 | -1.473344000 |
| H | -2.539881000 | 0.422507000 | -0.647465000 |
| H | 0.084634000 | 2.228855000 | -1.451944000 |
| H | 1.629105000 | 1.983260000 | -0.620351000 |
| H | 0.944650000 | 4.208349000 | -0.589964000 |
| H | -3.781329000 | -0.990385000 | 0.701755000 |
| H | 1.842331000 | -1.234714000 | -1.461344000 |
| H | 0.924590000 | -2.454557000 | -0.577849000 |
| H | 3.584558000 | -1.732979000 | -0.015781000 |

Sum of electronic and zero-point Energies= -517.473018
Sum of electronic and thermal Energies= -517.460128

Sum of electronic and thermal Enthalpies= -517.459184
Sum of electronic and thermal Free Energies= -517.513101

**TEOA in Gas phase**

| | | | |
|---|---|---|---|
| N | 0.006421000 | -0.006356000 | 0.113021000 |
| C | -0.577225000 | 1.258365000 | 0.514296000 |
| C | -0.802342000 | -1.148539000 | 0.485915000 |
| C | 1.393488000 | -0.143699000 | 0.510620000 |
| C | -1.932899000 | -1.389882000 | -0.498901000 |
| C | -0.267490000 | 2.353846000 | -0.482344000 |
| O | -0.789652000 | 3.560535000 | 0.032147000 |
| O | -2.740021000 | -2.484328000 | -0.125672000 |
| C | 2.185176000 | -0.981219000 | -0.479090000 |
| O | 3.507151000 | -1.215997000 | -0.047341000 |
| H | -1.662882000 | 1.153366000 | 0.572684000 |
| H | -0.238968000 | 1.572805000 | 1.515213000 |
| H | -1.216396000 | -1.037020000 | 1.505982000 |
| H | -0.176636000 | -2.043483000 | 0.506233000 |
| H | 1.497201000 | -0.578939000 | 1.518619000 |
| H | 1.842087000 | 0.854323000 | 0.555730000 |
| H | -1.510740000 | -1.633793000 | -1.475527000 |
| H | -2.532106000 | -0.477647000 | -0.624657000 |
| H | -0.719668000 | 2.091596000 | -1.447329000 |
| H | 0.818682000 | 2.425082000 | -0.634947000 |
| H | -0.643013000 | 4.258402000 | -0.609150000 |
| H | -3.165704000 | -2.277000000 | 0.710081000 |
| H | 2.154891000 | -0.496743000 | -1.463743000 |
| H | 1.728690000 | -1.967085000 | -0.592370000 |
| H | 3.967542000 | -0.374274000 | -0.004009000 |

Sum of electronic and zero-point Energies= -517.460419
Sum of electronic and thermal Energies= -517.447528
Sum of electronic and thermal Enthalpies= -517.446583
Sum of electronic and thermal Free Energies= -517.500521

**TEOA$^{•+}$ in DCM**

| | | | |
|---|---|---|---|
| N | 0.028783000 | -0.030698000 | 0.770576000 |
| C | -0.804085000 | -1.203546000 | 0.742418000 |
| C | 1.451126000 | -0.170684000 | 0.787254000 |
| C | -0.566686000 | 1.269744000 | 0.805942000 |
| C | 2.028901000 | -0.672848000 | -0.569047000 |
| C | -1.662885000 | -1.312244000 | -0.520987000 |
| O | -2.449934000 | -2.454639000 | -0.320921000 |
| O | 3.417667000 | -0.724104000 | -0.487974000 |
| C | -0.378182000 | 2.063096000 | -0.520759000 |
| O | -0.918290000 | 3.337744000 | -0.376013000 |
| H | -0.177728000 | -2.088893000 | 0.844937000 |
| H | -1.478349000 | -1.149794000 | 1.606131000 |
| H | 1.707226000 | -0.906653000 | 1.556605000 |
| H | 1.906767000 | 0.786643000 | 1.035454000 |
| H | -0.081066000 | 1.847017000 | 1.598253000 |

| | | | |
|---|---|---|---|
| H | -1.628213000 | 1.167247000 | 1.029519000 |
| H | 1.774635000 | 0.043251000 | -1.351402000 |
| H | 1.589129000 | -1.640840000 | -0.826505000 |
| H | -1.018462000 | -1.409400000 | -1.402253000 |
| H | -2.277877000 | -0.412293000 | -0.638457000 |
| H | -2.996175000 | -2.593293000 | -1.099840000 |
| H | 3.687648000 | -1.536063000 | -0.047587000 |
| H | -0.829262000 | 1.509709000 | -1.349979000 |
| H | 0.687957000 | 2.183720000 | -0.720031000 |
| H | -1.872385000 | 3.301402000 | -0.498533000 |

Sum of electronic and zero-point Energies= -517.265783
Sum of electronic and thermal Energies= -517.252810
Sum of electronic and thermal Enthalpies= -517.251866
Sum of electronic and thermal Free Energies= -517.306319

**$TEOA^{•+}$ in Gas phase**

| | | | |
|---|---|---|---|
| N | -0.019779000 | -0.008423000 | 0.827623000 |
| C | -1.422294000 | -0.321357000 | 0.783832000 |
| C | 0.940466000 | -1.063592000 | 0.826028000 |
| C | 0.414871000 | 1.350821000 | 0.813550000 |
| C | 0.993826000 | -1.839301000 | -0.531336000 |
| C | -2.096372000 | 0.071698000 | -0.537105000 |
| O | -3.423269000 | -0.315243000 | -0.365740000 |
| O | 2.084142000 | -2.690152000 | -0.520104000 |
| C | 1.074793000 | 1.774152000 | -0.540172000 |
| O | 1.603358000 | 3.044658000 | -0.401059000 |
| H | -1.557617000 | -1.389395000 | 0.951012000 |
| H | -1.923743000 | 0.224555000 | 1.590884000 |
| H | 0.671617000 | -1.777294000 | 1.612061000 |
| H | 1.931377000 | -0.659112000 | 1.029258000 |
| H | 1.183578000 | 1.483805000 | 1.580706000 |
| H | -0.432188000 | 2.001051000 | 1.031512000 |
| H | 1.145944000 | -1.122481000 | -1.340746000 |
| H | 0.042996000 | -2.355641000 | -0.699653000 |
| H | -1.609667000 | -0.449095000 | -1.372552000 |
| H | -1.995260000 | 1.153424000 | -0.699239000 |
| H | -3.921344000 | -0.162554000 | -1.173884000 |
| H | 1.854006000 | -3.542930000 | -0.139298000 |
| H | 0.339531000 | 1.697420000 | -1.347730000 |
| H | 1.900954000 | 1.092939000 | -0.756034000 |
| H | 0.962681000 | 3.715637000 | -0.653221000 |

Sum of electronic and zero-point Energies= -517.188710
Sum of electronic and thermal Energies= -517.175571
Sum of electronic and thermal Enthalpies= -517.174627
Sum of electronic and thermal Free Energies= -517.229696

**TEA in DCM**

| | | | |
|---|---|---|---|
| N | -0.000368000 | -0.405317000 | -0.262038000 |
| C | 0.000234000 | 0.997041000 | -0.653396000 |
| H | 0.869909000 | 1.163877000 | -1.293043000 |

| | | | |
|---|---|---|---|
| H | -0.870009000 | 1.164913000 | -1.292014000 |
| C | 1.190998000 | -0.817386000 | 0.460256000 |
| H | 1.083570000 | -1.888852000 | 0.656982000 |
| H | 1.259727000 | -0.334373000 | 1.451956000 |
| C | -1.191863000 | -0.816176000 | 0.460642000 |
| H | -1.084926000 | -1.887489000 | 0.658443000 |
| H | -1.260420000 | -0.332198000 | 1.451866000 |
| C | -2.482100000 | -0.585556000 | -0.300021000 |
| H | -3.310484000 | -1.065773000 | 0.225130000 |
| H | -2.726734000 | 0.474446000 | -0.393567000 |
| H | -2.419848000 | -1.012000000 | -1.304255000 |
| C | 0.001506000 | 2.021713000 | 0.476135000 |
| H | 0.885364000 | 1.926203000 | 1.111281000 |
| H | 0.001304000 | 3.031750000 | 0.059742000 |
| H | -0.881283000 | 1.926620000 | 1.112844000 |
| C | 2.481420000 | -0.586767000 | -0.300056000 |
| H | 2.726425000 | 0.473184000 | -0.393070000 |
| H | 3.309487000 | -1.067513000 | 0.225106000 |
| H | 2.419320000 | -1.012791000 | -1.304487000 |

Sum of electronic and zero-point Energies= -291.965566
Sum of electronic and thermal Energies= -291.956250
Sum of electronic and thermal Enthalpies= -291.955306
Sum of electronic and thermal Free Energies= -291.999289

**TEA$^{\bullet+}$ in DCM**

| | | | |
|---|---|---|---|
| N | 0.021760000 | -0.308763000 | -0.045015000 |
| C | 0.011237000 | 1.008119000 | -0.621270000 |
| H | 0.935901000 | 1.153206000 | -1.172699000 |
| H | -0.824799000 | 1.060298000 | -1.320192000 |
| C | 1.234906000 | -1.060889000 | 0.176756000 |
| H | 1.117768000 | -1.988698000 | -0.401084000 |
| H | 1.205896000 | -1.376580000 | 1.227644000 |
| C | -1.196803000 | -0.886480000 | 0.480189000 |
| H | -1.020679000 | -1.958217000 | 0.580723000 |
| H | -1.303778000 | -0.492067000 | 1.501466000 |
| C | -2.441773000 | -0.603471000 | -0.331979000 |
| H | -3.262768000 | -1.168416000 | 0.109411000 |
| H | -2.721258000 | 0.449775000 | -0.318375000 |
| H | -2.320921000 | -0.931536000 | -1.365267000 |
| C | -0.145865000 | 2.080465000 | 0.460201000 |
| H | 0.680401000 | 2.046134000 | 1.170012000 |
| H | -0.144970000 | 3.052408000 | -0.032697000 |
| H | -1.085304000 | 1.965872000 | 1.000808000 |
| C | 2.531589000 | -0.370910000 | -0.157828000 |
| H | 2.657171000 | 0.560714000 | 0.395007000 |
| H | 3.343986000 | -1.038836000 | 0.129377000 |
| H | 2.631292000 | -0.173714000 | -1.225431000 |

Sum of electronic and zero-point Energies= -291.766698
Sum of electronic and thermal Energies= -291.757243
Sum of electronic and thermal Enthalpies= -291.756299
Sum of electronic and thermal Free Energies= -291.801226

**TEA in Gas phase**

| | | | |
|---|---|---|---|
| N | -0.000336000 | -0.400712000 | -0.248921000 |
| C | 0.000333000 | 0.995808000 | -0.650728000 |
| H | 0.869327000 | 1.157339000 | -1.292679000 |
| H | -0.868981000 | 1.158383000 | -1.291986000 |
| C | 1.191753000 | -0.816534000 | 0.464711000 |
| H | 1.081836000 | -1.888104000 | 0.660177000 |
| H | 1.271545000 | -0.336642000 | 1.458049000 |
| C | -1.192612000 | -0.815256000 | 0.465109000 |
| H | -1.083171000 | -1.886650000 | 0.661765000 |
| H | -1.272256000 | -0.334282000 | 1.457917000 |
| C | -2.477322000 | -0.590293000 | -0.306721000 |
| H | -3.308182000 | -1.080189000 | 0.205402000 |
| H | -2.730507000 | 0.468194000 | -0.395588000 |
| H | -2.397952000 | -1.008837000 | -1.312700000 |
| C | 0.001353000 | 2.028947000 | 0.471703000 |
| H | 0.884071000 | 1.936711000 | 1.108968000 |
| H | 0.001834000 | 3.037564000 | 0.051764000 |
| H | -0.881200000 | 1.937852000 | 1.109383000 |
| C | 2.476669000 | -0.591382000 | -0.306686000 |
| H | 2.730283000 | 0.467073000 | -0.394652000 |
| H | 3.307181000 | -1.082009000 | 0.205307000 |
| H | 2.397481000 | -1.009156000 | -1.313004000 |

Sum of electronic and zero-point Energies= -291.963549
Sum of electronic and thermal Energies= -291.954252
Sum of electronic and thermal Enthalpies= -291.953308
Sum of electronic and thermal Free Energies= -291.997187

**$TEA^{\bullet+}$ in Gas phase**

| | | | |
|---|---|---|---|
| N | -0.019939000 | -0.307224000 | -0.041331000 |
| C | -0.014023000 | 1.009623000 | -0.620281000 |
| H | 0.821855000 | 1.063832000 | -1.320230000 |
| H | -0.941290000 | 1.150469000 | -1.169745000 |
| C | 1.204444000 | -0.880040000 | 0.482503000 |
| H | 1.027876000 | -1.950729000 | 0.602284000 |
| H | 1.322392000 | -0.470071000 | 1.497480000 |
| C | -1.236670000 | -1.054108000 | 0.198915000 |
| H | -1.111411000 | -2.006878000 | -0.336223000 |
| H | -1.218556000 | -1.326419000 | 1.263857000 |
| C | -2.534368000 | -0.385681000 | -0.176364000 |
| H | -3.348530000 | -1.049264000 | 0.117241000 |
| H | -2.679966000 | 0.560949000 | 0.345699000 |
| H | -2.622827000 | -0.222726000 | -1.251234000 |
| C | 0.140720000 | 2.086171000 | 0.460390000 |
| H | 1.090442000 | 1.990729000 | 0.987202000 |
| H | 0.116420000 | 3.059725000 | -0.029671000 |
| H | -0.674788000 | 2.042201000 | 1.182769000 |
| C | 2.445662000 | -0.610870000 | -0.342474000 |
| H | 2.727374000 | 0.442028000 | -0.347044000 |
| H | 3.271788000 | -1.167158000 | 0.101145000 |
| H | 2.324210000 | -0.956691000 | -1.370348000 |

| | |
|---|---|
| Sum of electronic and zero-point Energies= | -291.698548 |
| Sum of electronic and thermal Energies= | -291.688926 |
| Sum of electronic and thermal Enthalpies= | -291.687982 |
| Sum of electronic and thermal Free Energies= | -291.733909 |
